# Supplementary material for: Dynamics of the blood plasma proteome during hyperacute HIV-1 infection
Source: Nat Commun. 2024 Dec 5;15:10593. doi: 10.1038/s41467-024-54848-0 (PMC11618498; doi:10.1038/s41467-024-54848-0)
Supplement: Supplementary file 1 — Supplementary Information [file 41467_2024_54848_MOESM1_ESM.pdf]

SUPPLEMENTARY INFORMATION

Dynamics of the blood plasma proteome during hyperacute HIV-1 infection

Jamirah Nazziwa[1,2], Eva Freyhult[3], Mun-Gwan Hong[4], Emil Johansson[1,2], Filip Årman[5], Jonathan Hare[6,7], Kamini Gounder[8,9,10], Melinda Rezeli[5,11], Tirthankar Mohanty[12], Sven Kjellström[5], Anatoli Kamali[7], Etienne Karita[13], William Kilembe[14], Matt A Price[7,15], Pontiano Kaleebu[16], Susan Allen[13,14,17], Eric Hunter[13,14,17], Thumbi Ndung'u[8,9,10,18], Jill Gilmour[19], Sarah L. Rowland-Jones[20], Eduard Sanders[21,22,23], Amin S. Hassan[1,22,24§], and Joakim Esbjörnsson[1,2,20§*]

[1]Department of Translational Medicine, Lund University, Sweden

[2]Lund University Virus Centre, Lund University, Sweden

[3]National Bioinformatics Infrastructure Sweden, Science for Life Laboratory, Department of Cell and Molecular Biology, Uppsala University, Uppsala, Sweden

[4]National Bioinformatics Infrastructure Sweden, Science for Life Laboratory, Department of Biochemistry and Biophysics, Stockholm University, Stockholm, Sweden

[5]BioMS−Swedish National Infrastructure for Biological Mass Spectrometry, Lund University, Lund, Sweden

[6]IAVI Human Immunology Laboratory, Imperial College, London, United Kingdom

[7]IAVI, New York, New York, USA, and Nairobi, Kenya

[8]Africa Health Research Institute, Durban, South Africa

[9]HIV Pathogenesis Programme, The Doris Duke Medical Research Institute, University of KwaZulu-Natal, Durban, South Africa

[10]Division of Infection and Immunity, University College London, London, United Kingdom

[11]Department of Biomedical Engineering, Faculty of Engineering, Lund University, Lund, Sweden

[12]Division of Infection Medicine, Department of Clinical Sciences Lund, Faculty of Medicine, Lund University, Lund, Sweden
[13]Center for Family Health Research, Kigali, Rwanda
[14]Center for Family Health Research, Lusaka, Zambia
[15]UCSF Department of Epidemiology and Biostatistics, San Francisco, California, USA
[16]Medical Research Council/Uganda Virus Research Institute and London School of Hygiene and Tropical Medicine, Uganda Research Unit, Uganda
[17]Department of Pathology & Laboratory Medicine, School of Medicine, Emory University, Atlanta, Georgia, USA
[18]Ragon Institute of Massachusetts General Hospital, Massachusetts Institute of Technology and Harvard University, Cambridge, Massachusetts, USA
[19]Department of Infectious Diseases, Infection and Immunity, Faculty of Medicine, Imperial College, London, United Kingdom
[20]Nuffield Department of Medicine, University of Oxford, UK
[21]Sir William Dunn School of Pathology, University of Oxford, UK
[22]KEMRI/Wellcome Trust Research Programme, Kilifi, Kenya
[23]The Aurum Institute, Johannesburg, South Africa
[24]Institute for Human Development, Aga Khan University, Nairobi, Kenya

These authors contributed equally: Amin S. Hassan. Joakim Esbjörnsson.
*Correspondence and requests for materials should be addressed to J.E. (email: joakim.esbjornsson@med.lu.se)

SUPPLEMENTARY METHODS

Sample preparation for LC-MS/MS analysis

Neat plasma

The protein digestion process was optimized for 1 µl (1 mg/ml) of plasma. First, 50µl of digestion buffer (8 M urea, in 100 mM ammonium bicarbonate) was added to the plasma. Next, proteins were reduced with 5 mM Tris(2-carboxyethyl) phosphine, pH 7.0 for 60 minutes at 37 °C, and then alkylated with 25 mM iodoacetamide (Sigma) at room temperature for 30 minutes in the dark. The mixture was diluted with 100 mM ammonium bicarbonate to achieve a final urea concentration below 1.5 M, and trypsin (1/100, w/w, Sequencing Grade Modified Trypsin, Porcine; Promega) was added for overnight digestion (at least 9 hours) at 37 °C. Digestion was halted using 5% trifluoracetic acid (TFA) (Sigma) to pH 2-3, and the peptides were purified and desalted using SOLAµ^{TM} reverse phase solid phase extraction plates (Thermo Fisher Scientific), following the manufacturer's instructions. After washing with 50% acetonitrile with 0.1% TFA, the solvents were evaporated using a vacuum concentrator (Genevac, miVac), and the peptides were resuspended in 50µl HPLC-water (Fisher Chemical) containing 2% acetonitrile and 0.1% formic acid (Sigma).

An equivalent sample amount of 1µg peptide was then injected into the column, and the samples were spiked with indexed retention time peptides (iRT) peptides before LC-MS analysis. The LC-MS analysis for neat plasma was conducted using an EASY-nLC 1200 ultra-HPLC system coupled to a Q Exactive HF-X mass spectrometer (Thermo Fisher Scientific) with the following column setting: trap column (PepMap100 C18 3 µm; 75 µm × 2 cm; Thermo Fisher Scientific), EASY-Spray column (ES803, column temperature 45 °C; Thermo Fisher Scientific), and linear gradient from 5% to 38% over 90 or 120 minutes at a flow rate of 350 nl/min. The DIA-44 variable windows + 90 min NL gradientnLC was used for LC-MS/MS

analysis. The full MS resolution was set to 60,000 at 200 m/z, and the mass range was set to 350–1650 m/z.

For neat plasma, a spectral library was established using the Pulsar search engine integrated into Spectronaut 15.1(Biognosys, Schlieren, Switzerland) with the factory default settings. Briefly, peptides from 24 immunodepleted plasma were pooled and fractionated with off-line high-pH reversed-phase chromatography (Thermo Fisher Scientific). The fractions were then subjected to DDA analysis, and the raw DIA and DDA data were loaded directly into Pulsar and searched against the human reference proteome acquired from Uniprot Homo sapiens Database (December 2019, 42,410 entries). The generated library consisted of 15,896 precursors: 8616 peptides, 781 protein groups, and 1272 proteins.

Depleted plasma

To enhance the detection of medium and low range proteins in plasma, a depletion strategy was employed to remove high abundant proteins. Specifically, 95% of the top 14 most abundant proteins, including HSA, albumin, IgG, IgA, IgM, IgD, IgE kappa and lambda light chains, alpha-1-acid glycoprotein, alpha-1-antitrypsin, alpha-2-macroglobulin, apolipoprotein A1, fibrinogen, haptoglobin, and transferrin, were removed to increase the sensitivity of detection for low abundance proteins. The plasma samples were processed using High Select™ Top14 Abundant Protein Depletion Mini Spin Columns according to the manufacturer's protocol, which involved the use of resins containing highly specific immobilized antibodies. Briefly, four µl plasma was added to the column, mixed for 10 min at room temperature, and the eluent was collected by centrifugation at 1,000 × g for 2 min.

The depleted plasma was then subjected to digestion using the filter-aided sample preparation (FASP) method. To denature the proteins, 8M urea in 100 mM Ammonium bicarbonate was

used, and disulfide bridges were reduced with freshly prepared 35mM dithiothreitol (DTT). The proteins were then alkylated with 55mM iodoacetamide (IAA) to allow immediate access of the trypsin to the internal cleavage sites. Ultrafiltration facilitated by centrifugation was employed to remove DTT and other low-molecular-weight components. The filters were washed with 100mM Ammonium bicarbonate in between the different steps to ensure the removal of any remaining DTT or IAA.

Prior to protein digestion, the concentration of the protein was measured using the Qubit™ 4 Fluorometer (#Q33239, Thermo Fisher Scientific, Waltham, MA, USA) and the Invitrogen Qubit™ Protein BR Assay Kit (#Q33211, Thermo Fisher Scientific, Waltham, MA, USA). Sequencing Grade Modified Trypsin (1µg/µl,#V5111, Promega, Madison, WI, USA) was added to 30 µg protein for each sample in 100mM ammonium bicarbonate and incubated at 37°C for 16 hours. The digestion was halted by adding 10% trifluoroacetic acid (TFA) (#302031, Sigma- Aldrich, Merck, Darmstadt, Germany), and the pH was checked using pH strips. The peptide concentration was measured using the Nanodrop™ 2000 spectrophotometer (#ThermoND-2000, Fisher Scientific, Waltham, MA, USA) after digestion.

For LC-MS analysis, an equal amount of 1 µg peptide was injected into the column, and the samples were spiked with indexed retention time peptides (iRT) before analysis. The Dionex Ultimate 3000 RSLCnano UPLC coupled to an Exploris 480 mass spectrometer with FAIMS (Thermo Fischer Scientific) was used for LC-MS analysis of depleted plasma. The column settings were as follows: trap column (PN 164535), anal column (ES802A) LC-MS/MS analysis (DIA): DIA-26 variable windows + FAIMS with 2 CVs (-45V & -60V), 90 min NL gradient. nLC: – non-linear gradient, 1 min at 5% B, in 75 min up to 25% B, in 9 min up to 32% B, in 6 min up to 45% B, in 2 min up to 95% B, 5 min at 95% B and 12 min equilibration at 5% B full MS - resolution: 120.000, normalized AGC target: 300%, maxIT: 45 ms, 380-

1100 m/z,DIA – 26 windows with variable width, resolution: 30.000, normalized AGC target: 1000%, maxIT: auto, NCE: 32, centroid.

Due to differences in instrumentation, a separate spectral library was generated for depleted plasma. A total of 10 immunodepleted plasma samples were pooled, fractionated (N=8), and analyzed by DDA (DDA-max speed [1.7s + 1.3s cycle time] FAIMS with two CVs [-45V and -60V], 90 min NL gradient). The generated raw DIA data was combined with the DDA data and searched against Uniprot Homo sapiens Database (July 2020, 42,386 entries) using pulsar. The resulting spectral library generated using Spectronaut 15.1(Biognosys, Schlieren, Switzerland), contained 17,727 precursors: 12,529 peptides, 1,294 protein groups, and 2,259 proteins.

DIA/SWATH-MS Targeted Data Extraction

DIA data files were analysed using Spectronaut 14.10.201222.47784 (Biognosys, Schlieren, Switzerland), against the spectral library using the BGS factory default settings. The identifications were filtered at an FDR of 1% at both peptide and protein levels. Spectronaut used retention time prediction based on iRT (28), the m/z dimension in the SWATH-MS data, mass accuracy and isotopic distribution of fragment ions to identify a peptide. For each targeted peptide, all available transitions were extracted, along with their corresponding decoy-transition groups, which were generated by pseudo-reversing the sequence of the targeted peptides.

HIV-1 Subtyping

HIV-1 env sequences (V1-V3, approximately 940 base pairs) were generated, and the HIV-1 subtype was phylogenetically determined as previously described[1]. Briefly, the general time-reversible (GTR) model of nucleotide substitution with gamma-distributed rate heterogeneity

was used to infer maximum likelihood trees. Branch support was assessed using the approximate likelihood ratio test based on the Shimodaira-Hasegawa (aLRT-SH) method, with branch support of ≥ 0.90 considered significant[2].

SUPPLEMENTARY TABLES

Table S1 | Characteristics of study participants diagnosed with hyperacute HIV-1 infection. This table presents the demographic and clinical characteristics of individuals diagnosed with hyperacute HIV-1 infection. Key variables include age, sex, and HIV-1 transmission route. Data are summarised for participants included in the study cohort, with total number and percentage for each variable.

Characteristics	Total (N = 54)	Female participants (N = 20)	Male participants (N = 34)
**Age in years**			
**Median age (IQR)**	24 (22-27)	22 (21-24)	26 (22-31)
**Below age 25 (%)**	32 (59)	15 (75)	17 (50)
**Above age 25 (%)**	22 (41)	5 (25)	17 (50)
**Country of collection (Site, %)**			
**Kenya (Kilifi)**	32 (59)	3 (15)	29 (85)
**Rwanda (Kigali)**	4 (7)	2 (10)	2 (6)
**Zambia (Lusaka)**	3 (6)	0 (0)	3 (9)
**South Africa (Durban)**	15 (28)	15 (75)	0 (0)
**HIV-1 subtype (%)**			
**A1**	31 (57)	5 (25)	26 (76)
**A2D**	1 (2)	0 (0)	1 (3)
**C**	20 (37)	15 (75)	5 (15)
**D**	1 (2)	0 (0)	1 (3)
**G**	1 (2)	0 (0)	1 (3)
**Risk group (%)**			
**DC**	7 (13)	2 (10)	5 (15)
**HET**	19 (35)	18 (90)	1 (3)
**MSM**	28 (52)	0 (0)	28 (82)
**Median time in days from EDI (IQR)**			
**Pre-infection (V0)**	62 (28-106)	59 (41-92)	71 (22-113)
**After-Infection – Fiebig stage I (V1)**	10 (7-14)	7 (7-10)	9 (10-14)
**After-Infection – Fiebig stage II (V2)**	31 (28-37)	29 (28-31)	32 (29-39)

Abbreviations: HIV-1, human immunodeficiency virus type 1; IQR, interquartile range. Risk group data: DC (serodiscordant couples), HET (heterosexual) and MSM (men who have sex with men). Availability of matched pre-infection samples by days from sampling to the estimated date of infection (EDI).

Table S2 | HLA Alleles and disease progression classification across study participants. This table summarises the distribution of HLA alleles among study participants and their associated HIV-1 disease progression classifications. Disease progression is categorised as fast or slow, based on Time to CD4+ T-cell count <500 cells/mm^3 within 12 months from EDI and viral load dynamics over one year.

ID	sex	cohort	HLA-A	HLAA	HLAB	HLAB	HLAC	HLAC	VL	progression
1	Female	Durban	A*02:01	A*30:01	B*44:03	B*58:02	C*04:01	C*06:02	high	fast
2	Female	Durban	A*68:01	A*68:02	B*57:02	B*58:02	C*06:02	C*18:01	high	fast
3	Female	Durban	A*02:01	A*30:01	B*15:10	B*42:02	C*08:04	C*17:01	high	fast
4	Male	IAVI	A*01:01	A*30:02	B*15:03	B*58:02	C*02:10	C*06:02	high	fast
5	Female	IAVI	A*30:02	A*74:01	B*49:01	B*53:01	C*04:01	C*07:01	high	fast
6	Female	Durban	A*03:01	A*74	B*15:03	B*58:02	C*02:10	C*06:02	high	fast
7	Female	Durban	A*23:01	A*68:02	B*08:01	B*58:01	C*03:04	C*07:01	high	fast
8	Male	IAVI	A*34:02	A*36:01	B*53:01	B*53:01	C*04:01	C*04:01	high	fast
9	Male	IAVI	A*23:01	A*68:01	B*07:02	B*13:02	C*07:02	C*16:04	high	fast
10	Male	IAVI	A*68:01	A*68:02	B*15:10	B*58:02	C*03:04	C*06:02	high	fast
11	Male	IAVI	A*68:02	A*68:02	B*14:01	B*15:10	C*03:04	C*08:02	high	fast
12	Male	IAVI	A*02:01	A*68:02	B*27:03	B*58:01	C*02:02	C*03:02	high	fast
13	Male	IAVI	A*30:02	A*36:01	B*15:03	B*53:01	C*02:10	C*04:01	high	fast
14	Male	IAVI	A*68:02	A*68:02	B*07:02	B*53:01	C*06:02	C*07:02	high	fast
15	Male	IAVI	A*01:01	A*68:02	B*07:02	B*58:01	C*07:01	C*07:02	high	fast
16	Male	IAVI	A*36:01	A*74:01	B*53:01	B*58:01	C*04:01	C*06:02	high	fast
17	Male	IAVI	A*02:02	A*68:02	B*07:02	B*15:03	C*02:10	C*07:02	high	fast
18	Male	IAVI	A*02:01	A*30:02	B*15:10	B*45:01	C*04:01	C*16:01	high	fast
19	Male	IAVI	A*02:01	A*30:01	B*15:03	B*15:10	C*03:04	C*08:02	high	fast
20	Male	IAVI	A*01:01	A*02:02	B*45:01	B*58:01	C*06:02	C*17:01	high	fast
21	Female	IAVI	A*30:02	A*68:02	B*15:10	B*45:01	C*03:04	C*16:01	high	fast
22	Male	IAVI	A*02:01	A*74:01	B*51:01	B*58:02	C*06:02	C*16:02	high	fast
23	Male	IAVI	A*23:01	A*29:02	B*15:03	B*45:01	C*02:10	C*06:02	high	fast

24	Male	IAVI	A*30:01	A*30:02	B*42:01	B*53:01	C*04:01	C*17:01	high	fast
25	Female	Durban	A*23:01	A*74	B*35:01	B*58:01	C*04:01	C*06:02	high	fast
26	Female	Durban	A*23:01	A*29:02	B*42:01	B*53:01	C*03:04	C*17	high	fast
27	Male	IAVI	A*30:02	A*68:02	B*14:02	B*58:01	C*07:01	C*08:02	low	fast
28	Female	IAVI	A*02:01	A*03:01	B*15:03	B*47:01	C*02:10	C*06:02	low	fast
29	Male	IAVI	n/a	n/a	n/a	n/a	n/a	n/a	low	fast
30	Male	IAVI	A*30:02	A*68:02	B*18:03	B*73:01	C*04:01	C*15:02	low	fast
31	Male	IAVI	A*03:01	A*74:01	B*47:03	B*58:01	C*03:02	C*07:01	low	fast
32	Female	IAVI	A*30:01	A*30:02	B*42:02	B*57:03	C*17:01	C*18:01	low	fast
33	Male	IAVI	A*02:01	A*34:02	B*27:03	B*58:02	C*02:02	C*06:02	low	fast
34	Male	IAVI	A*02:05	A*36:01	B*41:02	B*53:01	C*04:01	C*17:01	low	fast
35	Female	Durban	A*01:01	A*66:01	B*39:10	B*81:01	C*12:03	C*18	low	fast
36	Female	Durban	A*43:01	A*43:01	B*07:02	B*15:03	C*02:10	C*18	low	fast
37	Male	IAVI	A*30:02	A*68:01	B*45:01	B*58:02	C*04:01	C*16:01	n/a	fast
38	Female	IAVI	A*30:02	A*68:02	B*15:10	B*53:01	C*03:04	C*04:01	n/a	fast
39	Male	IAVI	A*30:01	A*68:01	B*37:01	B*42:01	C*07:01	C*17:01	n/a	fast
40	Male	IAVI	A*30:02	A*68:02	B*07:02	B*15:03	C*02:10	C*07:02	n/a	fast
41	Male	IAVI	A*03:01	A*30:01	B*15:10	B*15:47	C*02:10	C*03:04	n/a	fast
42	Female	Durban	A*02:05	A*02:05	B*58:01	B*58:01	C*07:01	C*07:01	n/a	fast
43	Female	Durban	A*24:02	A*29:02	B*07:02	B*44:03	C*07:01	C*07:02	high	slow
44	Male	IAVI	A*26:01	A*74:01	B*35:01	B*50:01	C*04:01	C*06:02	high	slow
45	Male	IAVI	A*02:01	A*66:01	B*15:03	B*58:02	C*02:10	C*06:02	high	slow
46	Male	IAVI	A*02:01	A*31:04	B*07:02	B*18:01	C*07:02	C*07:04	high	slow
47	Male	IAVI	A*02:05	A*68:02	B*15:10	B*35:01	C*03:04	C*04:01	high	slow
48	Male	IAVI	A*30:01	A*30:02	B*14:02	B*42:01	C*08:02	C*17:01	high	slow
49	Female	Durban	A*02:05	A*66:01	B*14:01	B*39:10	C*08:04	C*12:03	low	slow
50	Female	Durban	A*23:01	A*30:01	B*15:10	B*58:01	C*03:02	C*16:01	low	slow
51	Male	IAVI	A*02:02	A*68:02	B*42:01	B*53:01	C*06:02	C*17:01	low	slow
52	Male	IAVI	A*34:02	A*68:02	B*07:02	B*15:03	C*02:10	C*07:02	low	slow
53	Female	Durban	A*33:03	A*68:02	B*07:02	B*57:02	C*07:02	C*18	low	slow

54	Female	Durban	A*30:01	A*68:02	B*18:01	B*58:01	C*02:02	C*03:02	low	slow

Table S3 | Differentially expressed proteins between HIV-1 pre-infection and acute HIV-1 infection stages of HIV-1. The table presents a comprehensive description of differentially expressed proteins that were observed during acute stages of HIV-1 infection. Significance was determined using a stringent threshold of $p<0.005$, $q<0.005$, and log2FC>1 through Linear mixed models, while also ensuring differences were observed in both cohorts. The Protein ID column displays the UniProtKB/Swiss-Prot entry name for each protein, while the Protein description column provides the recommended full protein name from UniProtKB/Swiss-Prot. The Biological process column captures either biological process keywords from UniProtKB/TrEMBL or protein function information provided by the human protein atlas. Secretome location provides information on the predicted location of the protein based on signal peptide and transmembrane region prediction methods listed in HPA, or alternatively, a description of the subcellular location of the mature protein (including isoform locations if available) as described by UNIPROT. Tissue specificity was provided through extracted information from UNIPROT and HPA on the expression of a gene at the mRNA and protein level in cells or tissues of multicellular organisms.

Protein	Protein description	Biological process	Secretome location	Tissue specificity
**V1-V0: Increased at V1**				
VWF	Von Willebrand factor	Blood coagulation, Cell adhesion, Haemostasis	Secreted to blood	Plasma
FN1	Fibronectin 1	Acute phase, Angiogenesis, Cell adhesion, Cell shape	Secreted to blood	Liver
**V2-V0: Increased at V2**				
TTN	Titin	Cardiac muscle cell development	Leakage	Cardiac and skeletal muscle
VWF	Von Willebrand factor	Blood coagulation, Cell adhesion, Haemostasis	Secreted to blood	Plasma
FN1	Fibronectin 1	Acute phase, Angiogenesis, Cell adhesion, Cell shape	Secreted to blood	Liver
PAPOLA	Poly(A) polymerase alpha	mRNA processing	Leakage	Ubiquitous
RAB10	RAB10, member RAS oncogene family	Transport	Leakage	Hippocampus, testis
FLNA	Filamin A	Cilium biogenesis/degradation, actin cytoskeleton reorganisation	Leakage	Ubiquitous
HLA-A	Major histocompatibility complex, class I, A	Adaptive immunity, Host-virus interaction, Innate immunity	Leakage	Ubiquitous
MYH9	Myosin heavy chain 9	Cell adhesion, Cell shape	Leakage	Ubiquitous

V2-V0: Decreased at V2				
LTF	Lactotransferrin	Immunity, Ion transport, Osteo-genesis, Transcription regulation	Secreted to blood	Plasma, tears, saliva,
PRDX2	Peroxiredoxin 2	T-cell proliferation	Leakage	Ubiquitous
FGL1	Fibrinogen like 1	Adaptive immunity, regulation of T-cell activation	Secreted to blood	Liver
ATF6	Activating transcription factor 6	Transcription regulation, Unfolded response	Leakage	Ubiquitous
CA2	Carbonic anhydrase 2	One-carbon metabolic process, carbon dioxide transport	Leakage	Intestine, Stomach
CAT	Catalase	Cellular detoxification of hydrogen peroxide	Leakage	Liver
HPN	Hepsin	Negative regulation of apoptotic process	Leakage	Kidney, liver, pancreas
POSTN	Periostin	Cell adhesion	Secreted to extracellular matrix	Stomach, Skin
CA1	Carbonic anhydrase 1	One-carbon metabolic process, carbon dioxide transport	Leakage	Intestine, bone marrow
SPTA1	Spectrin alpha, erythrocytic 1	Cell shape	Leakage	Bone marrow

V2-V1: Decreased at V1				
HNRNPA2B1	Heterogeneous nuclear ribonucleo A2/B1	Host-virus interaction, mRNA processing/splicing/ transport	Leakage	Ubiquitous
PRDX2	Peroxiredoxin 2	T-cell proliferation	Leakage	Ubiquitous
MANBA	Mannosidase beta	Glyco catabolic process	Leakage	Ubiquitous
HPN	Hepsin	Positive regulation by host of viral transcription	Leakage	Liver, kidney
CA2	Carbonic anhydrase 2	One-carbon metabolic process, carbon dioxide transport	Leakage	Intestine, Stomach
CA1	Carbonic anhydrase 1	One-carbon metabolic process, carbon dioxide transport	Leakage	Intestine, Stomach
SPTA1	Spectrin alpha, erythrocytic 1	Cell shape	Leakage	Bone marrow
GRN	Granulin precursor	Astrocyte activation involved in immune response	Secreted to blood	Kidney,

184

Table S4 | Performance measures in predicting ARS across 50 test sets in PLS-DA.

model	ER	acc	auc	TN	TP	FP	FN
**v0v1v2**	0.36	0.64	0.75	0.17	0.47	0.23	0.12
**v0v10v20**	0.24	0.76	0.82	0.24	0.52	0.16	0.07
**v1v2**	0.46	0.54	0.69	0.13	0.41	0.28	0.18
**v10v20**	0.20	0.80	0.82	0.26	0.54	0.15	0.05
**v10**	0.32	0.68	0.80	0.22	0.46	0.19	0.13
**v20**	0.23	0.77	0.85	0.23	0.54	0.15	0.08

Table S5 | Proteins associated with acute retroviral syndrome. The table presents a comprehensive description of statistically significant proteins at two weeks post HIV-1 infection vs. pre-infection that are associated with ARS. Significance was determined using a stringent threshold of p<0.005, q<0.005, and log2FC>1 through linear mixed modelling, while also ensuring differences were observed in both cohorts. The Protein ID column displays the UniProtKB/Swiss-Prot entry name for each protein, while the Protein description column provides the recommended full protein name from UniProtKB/Swiss-Prot. The Biological process column captures either biological process keywords from UniProtKB/TrEMBL or protein function information provided by the human protein atlas. Secretome location provides information on the predicted location of the protein based on signal peptide and transmembrane region prediction methods listed in HPA, or alternatively, a description of the subcellular location of the mature protein (including isoform locations if available) as described by UNIPROT. Tissue specificity is provided through extracted information from UNIPROT and HPA on the expression of a gene at the mRNA and protein level in cells or tissues of multicellular organisms.

Protein	Protein description	Biological process	Secretome location	Tissue specificity
**V1-V0: Increased more among those with ARS**				
KRT9	Keratin 9	Epithelial cell differentiation	Leakage	Lymphoid tissue
LILRA3	Leukocyte immunoglobulin-like receptor subfamily A member 3	Adaptive immune response	Secreted	B-cells, lung
**V1-V0: Decreased more among those with ARS**				
SCGB1A1	Secretoglobin family 1A member 1	Inflammatory response, negative regulation of IFN-gamma/IL-13/IL-4 production	Secreted in other tissues	Club cells
ZYX	Zyxin	Inflammatory response, Cell adhesion, Host-virus interaction, response to IFN-gamma	Leakage	Ubiquitous
PPBP	Pro-platelet basic protein	Inflammatory response, Chemotaxis	Secreted to blood	Bone marrow, lymphoid tissue
FCGR3A	Fc fragment of IgG receptor IIIa	ADCC, regulation of immune response, epithelial cell differentiation	Secreted to blood	Plasma
GSN	Gelsolin	Cellular response to interferon-gamma, viral entry into host cell, Cilium biogenesis	Secreted to blood	Plasma

ICOSLG	Inducible T-cell costimulator ligand	Adaptive immunity, B-cell activation, Immunity	Membrane	Ubiquitous
ECMI	Extracellular matrix protein 1	Adaptive immunity, B-cell activation, Immunity	Secreted	Esophagus, epididymis
**V2-V0: Increased more among those with ARS**				
GDI2	GDP dissociation inhibitor 2	Signal transduction, vesicle mediated transport	Leakage	Brain
GSTO1	Glutathione S-transferase omega 1	Positive regulation of skeletal muscle contraction by regulation of release of sequestered calcium ion	Leakage	Ubiquitous
PSMA1	Proteasome 20S subunit alpha 1	Inflammatory response, proteasomal protein catabolic process	Leakage	Ubiquitous
TMOD3	Tropomodulin 3	Actin filament organisation	Leakage	Ubiquitous
TUBB	Tubulin beta class I	Cytoskeleton-dependent intracellular transport	Leakage	Spleen, thymus, brain
**V2-V0: Decreased more among those with ARS**				
C2	Complement C2	Complement activation, innate immunity	Leakage	Liver
CTSS	Cathepsin S	Adaptive immune response, antigen processing and presentation	Leakage	Lymphoid tissue, bone marrow
HRG	Histidine rich glycoprotein	Fibrinolysis, platelet activation, actin cytoskeleton organization	Secreted to blood	Plasma
ICOSLG	Inducible T-cell costimulator ligand	Adaptive immunity, B-cell activation, Immunity	Membrane	Ubiquitous
PTPRG	Protein tyrosine phosphatase receptor type G	Negative regulation of epithelial cell migration	Membrane	Ubiquitous
SCGB1A1	Secretoglobin family 1A member 1	Inflammatory response, negative regulation of IFN-gamma/IL-13/IL-4 production	Secreted in other tissues	Club cells
ECMI	Extracellular matrix protein 1	Adaptive immunity, B-cell activation, Immunity	Secreted	Esophagus, epididymis

Table S6 | Association between ARS, clinical parameters, and viral control. The table displays the results of various tests assessing the association of viral control with several variables, including acute retroviral syndrome (ARS), site, HIV-1 transmission risk group, age, sex, and HIV-1 subtype. Each row represents a specific variable, and the corresponding test was used to evaluate the association between viral controllers and non-viral controllers. Variables investigated include ARS, site, HIV-1 transmission risk group, age, sex, and HIV-1 subtype. The test column lists the several tests used to assess the association between the variable and viral control. The p-value associated with the test (all two-sided), representing the statistical significance of the results and providing evidence of no association, is shown in the last column. The numbers in parentheses indicate the percentage of participants for each variable among viral control groups.

Variable	Non-viral controllers	Viral controllers	Test	p-value
**N**	30	15	None	
**ARS=Yes (%)**	14 (58.3)	4 (50)	Fisher	0.703
Fever=Yes (%)	20 (83.3)	4 (50)	Fisher	0.152
Headache=Yes (%)	13 (54.2)	5 (62.5)	Fisher	1
Nightsweats=Yes (%)	13 (54.2)	5 (62.5)	Fisher	1
Myalgia=Yes (%)	16 (66.7)	5 (62.5)	Fisher	1
Fatigue=Yes (%)	17 (70.8)	5 (62.5)	Fisher	0.681
Skinrash=Yes (%)	0 (0)	1 (12.5)	Fisher	0.25
Oralulcers=Yes (%)	4 (16.7)	2 (25)	Fisher	0.625
Pharyngitis=Yes (%)	10 (41.7)	3 (37.5)	Fisher	1
Lymphadenopathy=Yes (%)	9 (37.5)	1 (12.5)	Fisher	0.38
Diarrhea=Yes (%)	8 (33.3)	1 (12.5)	Fisher	0.386
Anorexia=Yes (%)	16 (66.7)	4 (50)	Fisher	0.433
**Site (%)**			Fisher	0.338
Durban	6 (20)	6 (40)		
Kigali	2 (6.7)	2 (13.3)		
Kilifi	20 (66.7)	7 (46.7)		
Lusaka	2 (6.7)	0 (0)		

**Risk group (%)**			Fisher	0.337
DC	4 (13.3)	2 (13.3)		
HET	8 (26.7)	7 (46.7)		
MSM	18 (60)	6 (40)		
**Sex=Male (%)**	22 (73.3)	7 (46.7)	Chi square	0.152
**Age (mean (SD))**	25.52 (6.74)	27.65 (6.81)	Mann-Whitney U	0.0766
**Subtype (%)**	(%)		Fisher	0.649
A1	18 (60)	8 (53.3)		
A2D	1 (3.3)	0 (0)		
C	10 (33.3)	6 (40)		
D	0 (0)	1 (6.7)		
G	1 (3.3)	0 (0)		

Table S7 | Proteins associated with viral control. The table presents a comprehensive description of statistically significant/ proteins at two weeks post HIV-1 infection vs. pre-infection; one-month post HIV-1 infection vs. pre-infection; one-month vs two-week post HIV-1 infection; that are associated with viral control. To determine significance, a stringent threshold was set at $p<0.005$, $q<0.005$, and log2FC>1, while also ensuring differences were observed in both cohorts. The Protein ID column displays the UniProtKB/Swiss-Prot entry name for each protein, while the Protein description column provides the recommended full protein name from UniProtKB/Swiss-Prot. The Biological process column captures either biological process keywords from UniProtKB/TrEMBL or protein function information provided by the human protein atlas. Secretome location provides information on the predicted location of the protein based on signal peptide and transmembrane region prediction methods listed in HPA, or alternatively, a description of the subcellular location of the mature protein (including isoform locations if available) as described by UNIPROT. Tissue specificity is provided through extracted information from UNIPROT and HPA on the expression of a gene at the mRNA and protein level in cells or tissues of multicellular organisms.

Protein	Protein description	Biological process	Secretome location	Tissue specificity
**V1-V0: Increased more among viral controllers**				
NAPA	NSF attachment protein alpha	ER-Golgi transport, Protein transport	Leakage	
**V2-V0: Increased more among viral controllers**				
NAPA	NSF attachment protein alpha	ER-Golgi transport, Protein transport	Leakage	
RAN	GTP-binding nuclear protein Ran	Host-virus interaction, protein export from nucleus	Leakage	Plasma
**V2-V0: Decreased among viral controllers**				
ITIH4	Inter-alpha-trypsin inhibitor heavy chain 4	Acute phase	Secreted to blood	Liver
**V2-V1: Increased among viral controllers**				
RAN	GTP-binding nuclear protein Ran	Host-virus interaction, protein export from nucleus	Leakage	Plasma

Table S8 | Proteins associated with disease progression. The table presents a comprehensive description of statistically significant/ proteins at two weeks post HIV-1 infection vs. pre-infection; one-month post HIV-1 infection vs. pre-infection; one-month vs two-week post HIV-1 infection; that are associated with disease progression. To determine significance, a stringent threshold was at $p<0.005$, $q<0.005$, and log2FC>1, while also ensuring differences were observed in both cohorts. The Protein ID column displays the UniProtKB/Swiss-Prot entry name for each protein, while the Protein description column provides the recommended full protein name from UniProtKB/Swiss-Prot. The Biological process column captures either biological process keywords from UniProtKB/TrEMBL or protein function information provided by the human protein atlas. Secretome location provides information on the predicted location of the protein based on signal peptide and transmembrane region prediction methods listed in HPA, or alternatively, a description of the subcellular location of the mature protein (including isoform locations if available) as described by UNIPROT. Tissue specificity is provided through extracted information from UNIPROT and HPA on the expression of a gene at the mRNA and protein level in cells or tissues of multicellular organisms.

Protein	Protein description	Biological process	Secretome location	Tissue specificity
**V1-V0: Increase increases risk of progression /Increased among fast progressors**				
HPN	Hepsin	Positive regulation by host of viral transcription	Membrane	Liver, kidney
PRKCB	Protein kinase C beta	Adaptive immunity, apoptosis, transcription regulation	Leakage	
APOC4	Apolipoprotein C4	Lipid transport, transport	Secreted to blood	Liver, plasma
PSMB6	Proteasome 20S subunit beta 6	Host-virus interaction	Leakage	
TXNDC5	Thioredoxin domain containing 5	Negative regulation of apoptotic process, protein folding	Intracellular and membrane	
CRHBP	Corticotropin releasing hormone binding protein	Inflammatory response	Secreted to blood	
**V1-V0: Decrease increases risk of progression /Decreased among fast progressors**				
GSTM2	Glutathione S-transferase mu 2	Regulation of release of sequestered calcium ion	Leakage	Muscle
**V2-V0: Increase increases risk of progression /Increased among fast progressors**				
ITGB3	Integrin subunit beta 3	Cell adhesion, host-virus interaction	Intracellular and membrane	
DDTL	D-dopachrome tautomerase-like		Leakage	
UBB	Ubiquitin B	Protein ubiquitination	Leakage	
HSPA8	Heat shock protein family A (Hsp70) member 8	Host-virus interaction, mRNA processing, stress response, transcription	Leakage	Ubiquitous

V2-V0: Decrease increases risk of progression /Decreased among fast progressors

CD84	CD84 molecule	Adaptive immunity, autophagy, cell adhesion, innate immunity	Leakage	Hematopoietic tissues
LTBP1	Latent transforming growth factor beta binding protein 1	Regulation of transforming growth factor beta activation	Secreted to extracellular matrix	Heart

V2-V1: Increase increases risk of progression /Increased among fast progressors

CLU	Clusterin	Apoptosis, complement pathway, innate immunity	Secreted to blood	Plasma
C7	Complement C7	Complement alternate pathway, complement pathway, cytolysis, innate immunity	Secreted to blood	Plasma
C4BPA	Complement component 4 binding protein alpha	Complement pathway, innate immunity	Secreted to blood	Plasma

V2-V1: Decrease increases risk of progression /Decreased among fast progressors

OAF	Out at first homolog		Secreted - unknown location	
PEPD	Peptidase D	Collagen degradation		
PPIF	Peptidylprolyl isomerase F	Apoptosis, necrosis	Leakage	
CD84	CD84 molecule	Adaptive immunity, autophagy, cell adhesion, immunity, innate immunity	Membrane	Hematopoietic tissues
CLIC1	Chloride intracellular channel 1	Ion transport, transport	Leakage	Heart, placenta, liver, kidney, pancreas
SLTM	SAFB like transcription modulator	Apoptosis, transcription, transcription regulation	Leakage	
FOXA1	Forkhead box A1	Transcription, transcription regulation	Leakage	
RTN4	Reticulon 4	Neurogenesis	Membrane	
ALOX12	Arachidonate 12-lipoxygenase, 12S type	Fatty acid metabolism, lipid metabolism	Leakage	Vascular smooth muscle cell
SERPIND1	Serpin family D member 1	Blood coagulation, chemotaxis, haemostasis	Secreted to blood	Liver, plasma
APOB	Apolipoprotein B	Cholesterol metabolism, lipid metabolism, lipid transport, steroid metabolism, sterol metabolism, transport	Secreted to blood	

Table S9 | Model parameters for the different analyses: Time differences, ARS, viral control, and disease progression. This table outlines the model parameters used in the analysis of Acute Retroviral Syndrome (ARS), viral load, and disease progression, assessed through linear regression and Cox regression models. Parameters include predictor variables, coefficients, hazard ratios, confidence intervals, and p-values for each model. ARS and viral load were evaluated as continuous and categorical variables, while disease progression was classified into relevant categories (e.g., slow, rapid).

**Model type**	**Model equation**	**Model tested on visit**	**Dependent variable**	**Predictor variable**	**Covariates**	**Accuracy**	**AUROC**	**Misclassification error**	**Sample size per cohort**	**Inclusion/ Exclusion criteria**	**Scale/ measure**
Linear mixed model	$Protein_{ij}$ =<br>β_0<br>+<br>β_1Visit<br>+<br>β_2Cohort<br>+<br>β_3Age<br>+<br>Visit:Cohort<br>+<br>PC + P2<br>+<br>u_i<br>(Random effect for individual)	V0<br>V1<br>V2<br>V1-V0<br>V2-V0<br>V2-V1	Individual protein expression level	Visit/ timepoints<br>V0<br>V1<br>V2<br>V1-V0<br>V2-V0<br>V2-V1	Age<br><br>Cohort				N = 52<br><br>Durban: 15<br><br>IAVI: N = 37	Excluded: Incomplete data<br><br>Included: Only patients with an protein values at V0,V1,V2	Expression levels (log2)<br><br>Visit: categorical<br><br>Age: continuous<br><br>Individuals: 52 (2 missing data at V1/V2)

Model parameters for ARS analysis

Model type	Model equation	Model tested on visit	Dependent variable	Predictor variable	Covariates	Accuracy	AUROC	Misclassification error	Sample size per cohort	Inclusion/ Exclusion criteria	Scale/ measure
PLS-DA	NA	V0+V1+V2<br>V0+V10+V20<br>V1+V2<br>V10+V20<br>V10<br>V20	Protein Expression profiles	ARS status (binary)	Age<br><br>Cohort	78% (CV)	0.82 (average computed over 50 test sets)		Durban: NA<br><br>IAVI: N = 33<br><br>(ARS+ = 20, ARS- = 13)	Excluded: Incomplete data<br><br>Included: Only patients with an ARS value from LCA	Expression levels (log2)<br><br>ARS: Yes or No<br><br>Age: continuous
Linear model	$Protein_{ij} = \beta_0 + \beta_1 ARS + \beta_2 Visit + \beta_3 Age + ARS{:}Visit + u_i$ (Random effect for individual)	V0<br>V1<br>V2<br>V1-V0<br>V2-V0<br>V2-V1	Individual protein expression level	ARS status (binary)	Age				Durban: NA<br><br>IAVI: N = 33<br><br>(ARS+ = 20, ARS- = 13)	Excluded: Incomplete data<br><br>Included: Only patients with an ARS value from LCA	Expression levels (log2)<br><br>ARS: Yes or No<br><br>Age: continuous

Model parameters for viral control analysis

**Model type**	**Model equation**	**Model tested on visit**	**Dependent variable**	**Predictor variable**	**Covariates**	**Accuracy**	**AUROC**	**Misclassification error**	**Sample size per cohort**	**Inclusion/ Exclusion criteria**	**Scale/ measure**
Linear model	Proteinij = β0 + β1VL + β2Visit + β3Age + VL:Visit + ui (Random effect for individual)	V0<br>V1<br>V2<br>V1-V0<br>V2-V0<br>V2-V1	Individual protein expression level	Viral control (binary)	Age<br><br>Cohort				N = 45 (high = 30, Low = 15)<br><br>Durban: N =12 (high = 6, Low = 6)<br><br>IAVI: N = 33 (high = 24, Low = 9)	Excluded: Incomplete data, without VL results from 1-12 months post EDI<br><br>Included: Only patients with an protein values at V0,V1,V2	Expression levels (log2)<br><br>Viralcontrol: binary<br><br>Age: continuous<br><br>Individuals: 52

Model parameters for disease progression analysis

Model type	Model equation	Model tested on visit	Dependent variable	Predictor variable	Covariates	Accuracy	AUROC	Misclassification error	Sample size per cohort	Inclusion/ Exclusion criteria	Scale/ measure
Cox regression		V0<br>V1<br>V2<br>V1-V0<br>V2-V0<br>V2-V1	Individual protein expression level	Event: Time to cd4_abs<500 from 6 weeks after EDI (binary: Progressed-fast vs not progressed-Slow)	Age<br><br>Cohort<br><br>Sex				N = 54 (fast = 42, slow = 12)<br><br>Durban: N =15 (fast = 10, slow = 5)<br><br>IAVI: N = 39 (high = 32, Low = 7)	Included: Only patients with an protein values at V0,V1,V2	Expression levels (log2)<br><br>Disease progression: binary<br><br>Age: continuous<br><br>Individuals: 52

SUPPLEMENTARY FIGURES

Fig. S1 | Protein pre-processing and quality control results.

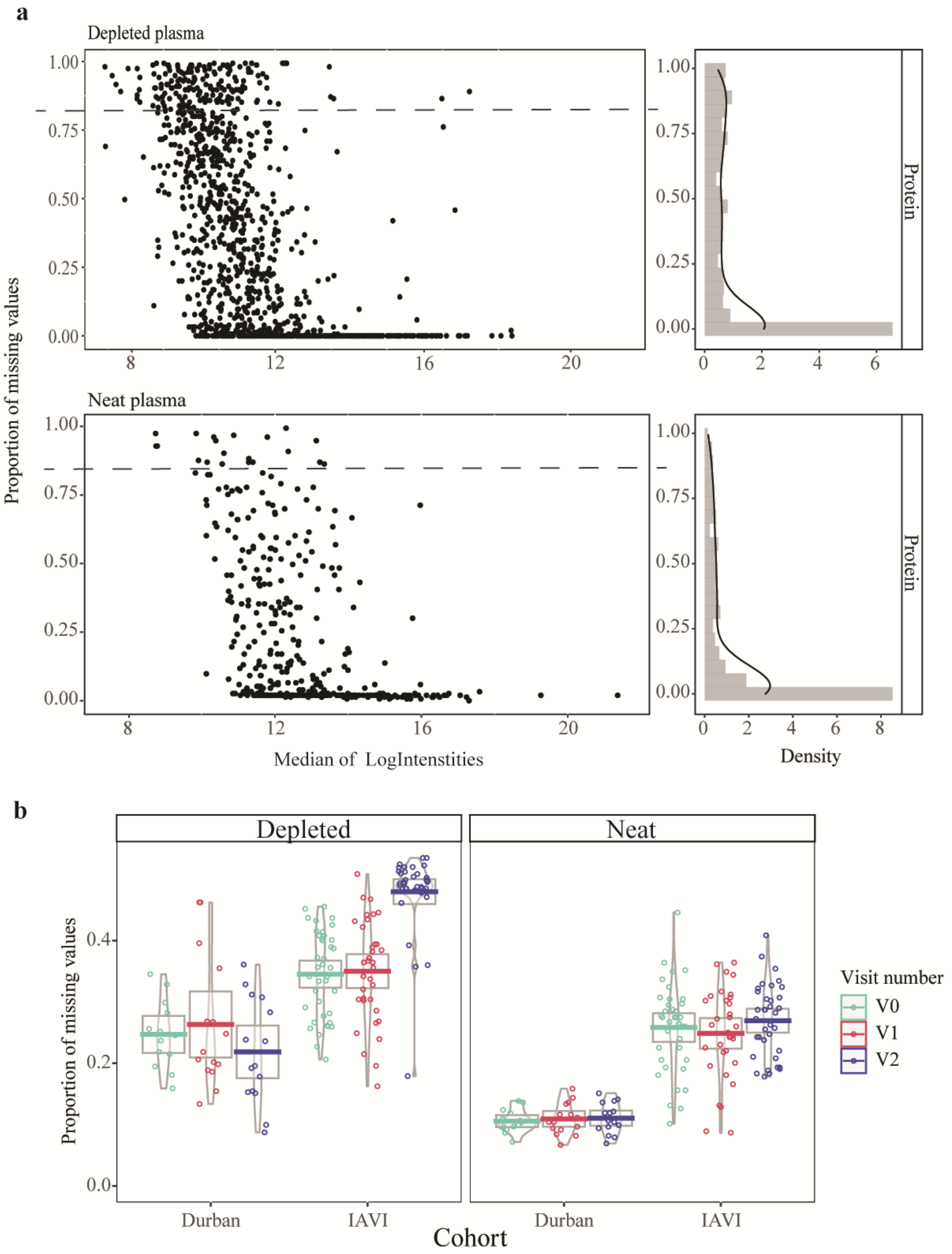

Fig. S1 | Protein pre-processing and quality control results. a, Scatterplot depicting the protein-wise relationship between median intensities and the proportion of missing values for

each sample preparation type in the left panel. Histograms illustrating the frequencies of the proportion are presented alongside with density plots to the right. Protein-wise investigation revealed inverse correlation indicating that proteins with lower median signals generally exhibited more missing values. These trends align with the assumption of missing values are not at random in DIA/SWATH data. **b**, Plot representing the proportion of missing values across cohorts. The IAVI cohort showed higher missingness across all time points when compared to the Durban cohort in both depleted and neat plasma samples ($p<0.005$; Welch Two Sample t-test). Abbreviations: V0, visit 0 (collected before estimated date of infection); V1, visit 1 (collected 10-14 days post estimated date of infection); V2, visit 2 (collected 15-42 days before estimated date of infection); IAVI, International AIDS Vaccine Initiative.

Fig. S2 | Longitudinal plasma proteome based on mean protein intensities.

a

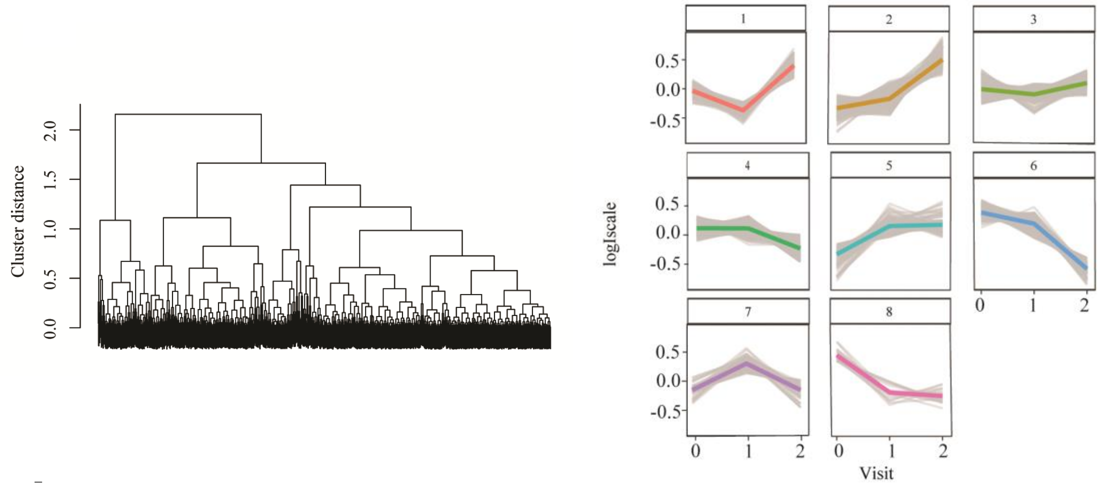

b

Cluster	Depleted	Neat	Enriched biological processes
1	79	19	cell adhesion<br>extracellular structure organisation
2	215	31	Epithelial cell /kidney development<br>complement activation<br>cellular extravasation<br>viral entry into host cell<br>extracellular structure organisation
3	227	157	blood coagulation, fibrin clot formation<br>regulation of glucose metabolic process<br>intermediate filament cytoskeleton organisation
4	179	111	activation of immune response<br>complement activation<br>pyruvate metabolic process<br>cytoskeleton organisation and protein transport
5	85	22	blood coagulation, fibrin clot formation<br>acute-phase response<br>activation of immune response
6	121	14	cytoskeleton organisation<br>ubiquitin-dependent protein catabolic process<br>regulation of apoptosis<br>regulation of cellular biosynthetic process
7	37	19	acute-phase response<br>activation of immune response
8	14	6	positive regulation of programmed cell death<br>blood coagulation, fibrin clot formation<br>negative regulation of hydrolase activity

Fig. S2 | Longitudinal plasma proteome based on mean protein intensities. a, Dendrogram illustrating hierarchical clustering with complete linkage for longitudinal protein expression profiles during AHI. The dendrogram was based on the mean protein expression profiles across all 54 patients i.e., 1336 protein combination values from all three time points were analysed. Optimal clusters, indicative of distinct longitudinal expression profiles, were identified using the elbow method, resulting in eight clusters. These clusters were then color-coded and plotted, with the x-axis representing the visit number and the y-axis reflecting the scaled mean log-intensity per protein. **b**, Summary of the number of proteins in both depleted and neat; and enriched biological process terms per cluster.

Fig. S3 | Protein expression different between cohorts.

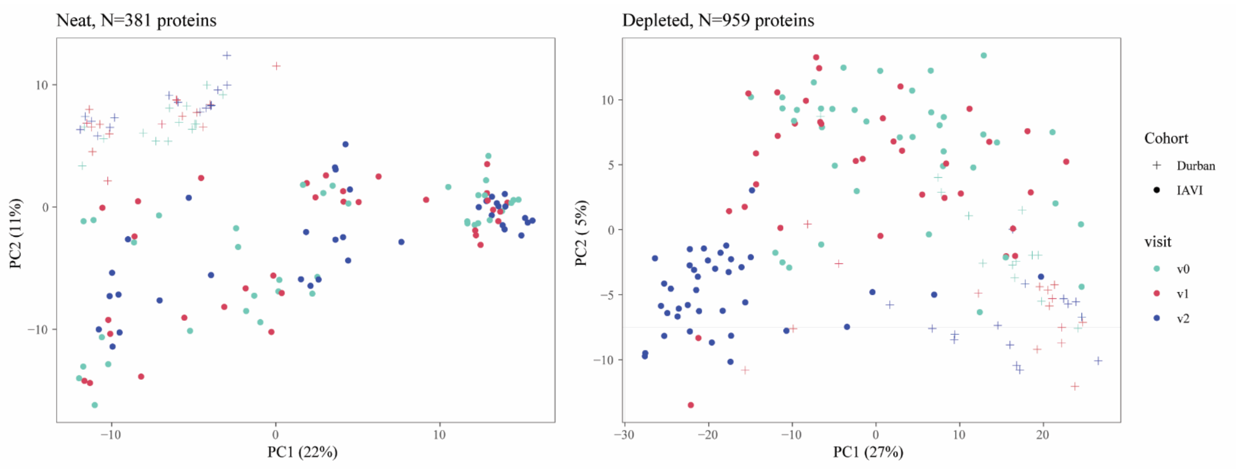

Fig. S3 | Protein expression different between cohorts. PCA plot illustrating protein expression profiles in AHI samples longitudinally collected from 54 participants across two cohorts. The x- and y- axes represent PCA 1 and 2, respectively, with the explained percentage variance indicated on the axis labels in bracket. Cohorts are distinguished by different symbols: "+" for the Durban cohort and "o" for the IAVI cohort. To preprocess the protein expression data, missing values imputed by replacing each with a randomly chosen value between one and the minimum of the protein that has the missing value. The data was then log-2 transformed and normalised using the Cyclic Loess method (normalizeCyclicLoess function in limma package v3.50.0) as determined by NormalyzerDE. Abbreviations: V0, visit 0 (collected before estimated date of infection); V1, visit 1 (collected 10-14 days post estimated date of infection); V2, visit 2 (collected 15-42 days before estimated date of infection); IAVI, International AIDS Vaccine Initiative. PC, principal component.

Fig. S4 | Acute HIV-1 associated tissue damage signatures.

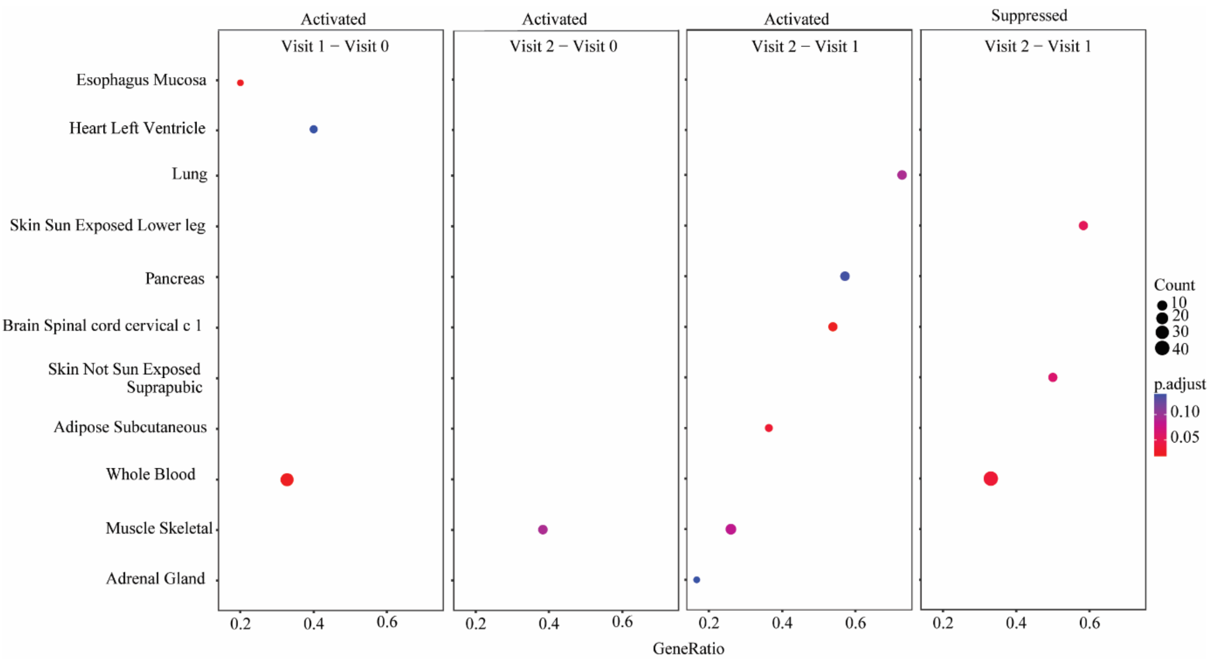

Fig. S4 | Acute HIV-1 associated tissue damage signatures. Dot plot illustrating the results of gene set enrichment analysis for tissue damage signatures obtained using a tissue damage library. Each dot represents a gene set and is positioned based on its enrichment score and statistical significance. The size of the dot corresponds to the significance of enrichment, with larger dots indicating higher significance. The color of the dot represents the adjusted p-value associated with the protein set. Dots located below the significance threshold ($p<0.05$) indicate positive enrichment, signifying overrepresentation of the gene set in the analysed data. The tissue damage signatures are displayed on the y-axis, while the x-axis represents the Protein/GeneRatio. Visit 0, collected before estimated date of infection; visit 1, collected 10-14 days post estimated date of infection; and visit 2, collected 15-42 days before estimated date of infection.

Fig. S5 | Classification of disease progression.

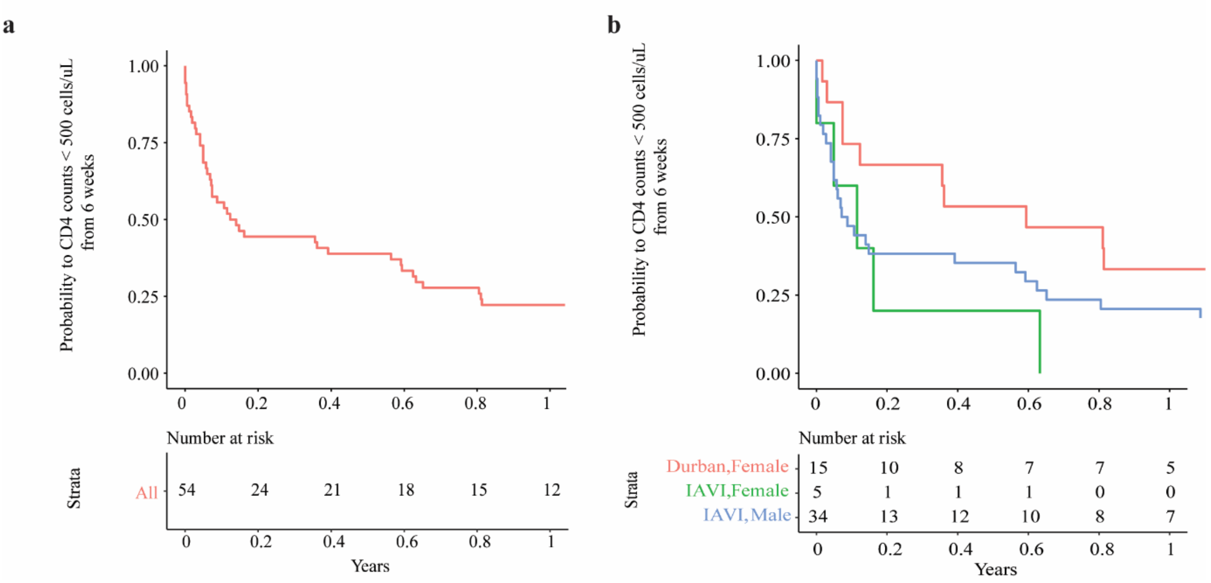

Fig. S5 | Classification of disease progression. a, Kaplan-Meier plot displaying the estimated probability of survival against time to CD4+ T-cell counts <500 cells/µl, starting from six weeks post estimated date of infection to ART start date. The number of study participants at risk at regular time intervals is shown at the bottom of the figure. **b**, Kaplan-Meier plot displaying the differences in survival between female and male study participants. A p-value of 0.08 from the Log-rank test is indicative of no significant difference in survival between female and male participants.

REFERENCES

1 Esbjornsson, J., Mild, M., Mansson, F., Norrgren, H. & Medstrand, P. HIV-1 Molecular Epidemiology in Guinea-Bissau, West Africa: Origin, Demography and Migrations. *PLoS ONE* **6**, doi:10.1371/journal.pone.0017025 (2011).

2 Guindon, S. *et al.* New Algorithms and Methods to Estimate Maximum-Likelihood Phylogenies: Assessing the Performance of PhyML 3.0. *Systematic biology* **59**, 307-321, doi:10.1093/sysbio/syq010 (2010).
