# Supplementary material for: Dynamics of the blood plasma proteome during hyperacute HIV-1 infection
Source: Nat Commun. 2024 Dec 5;15:10593. doi: 10.1038/s41467-024-54848-0 (PMC11618498; doi:10.1038/s41467-024-54848-0)
Supplement: Supplementary file 2 — Description of Additional Supplementary Files [file 41467_2024_54848_MOESM2_ESM.docx]

**Description of Additional Supplementary Files**

File Name: Supplementary Data 1 Description: **Previously determined associations between HIV-1 proteins and the differentially expressed plasma proteins related to acute retroviral syndrome (ARS).** The table includes: The HIV-1 protein name (HIV-1_Protein_Name); a descriptive keyword of the type of interaction between the HIV-1 protein and the differentially expressed plasma protein (Keyword); the human gene symbol of the differentially expressed plasma protein (Human_GeneSymbol); the PubMed identifier(s) of the related references (PMID(s)); and a description of the interaction between the HIV-1 protein and the differentially expressed plasma protein (Interaction_Desc).

File Name: Supplementary Data 2 Description: **Previously determined associations between HIV-1 proteins and the differentially expressed plasma proteins related to HIV-1 control.** The table includes: The HIV-1 protein name (HIV-1_Prot_Name); a descriptive keyword of the type of interaction between the HIV-1 protein and the differentially expressed plasma protein (Keyword); the human gene symbol of the differentially expressed plasma protein (Human_GeneSymbol); the human protein name (Human_Prot_Name); the PubMed identifier(s) of the related references (PMID(s)); and a description of the interaction between the HIV-1 protein and the differentially expressed plasma protein (Interaction_Desc).

File Name: Supplementary Data 3 Description: **Previously determined associations between HIV-1 proteins and the differentially expressed plasma proteins related to HIV-1 disease progression.** The table includes: The HIV-1 protein name (HIV-1_Prot_Name); a descriptive keyword of the type of interaction between the HIV-1 protein and the differentially expressed plasma protein (Keyword); the human gene symbol of the differentially expressed plasma protein (Human_GeneSymbol); the PubMed identifier(s) of the related references (PMID(s)); and a description of the interaction between the HIV-1 protein and the differentially expressed plasma protein (Interaction_Desc).

File Name: Supplementary Data 4 Description: **Previously determined associations between HIV-1 proteins and the differentially expressed plasma proteins in hyperacute HIV-1 infection.** The table includes: the human gene symbol of the differentially expressed plasma protein (Gene.Names); the HIV-1 gene identifier (HIV‐1_GeneID); the HIV-1 protein accession code (HIV‐1_Prot_Acc); The HIV-1 protein name (HIV-1_Prot_Name); a descriptive keyword of the type of interaction between the HIV-1 protein and the differentially expressed plasma protein (Keyword); the PubMed identifier(s) of the related references (PMID(s)); and a description of the interaction between the HIV-1 protein and the differentially expressed plasma protein (Interaction_Desc).
