# Supplementary material for: Dynamics of the blood plasma proteome during hyperacute HIV-1 infection
Source: Nat Commun. 2024 Dec 5;15:10593. doi: 10.1038/s41467-024-54848-0 (PMC11618498; doi:10.1038/s41467-024-54848-0)
Supplement: Supplementary file 3 — Supplementary Data 1 [file 41467_2024_54848_MOESM3_ESM.pdf]

HIV-1_Protein_Name	Keyword	Human_GeneSymbol	PMID(s)	Interaction_Desc
Vif	binds	ELOC	15574592, 18541215, 19008196, 19038776, 20096141, 20174454	HIV-1 Vif binding to ElonginC is negatively regulated by serine phosphorylation in the BC-box of the Vif SOCS-box motif, and mutating amino acid S144 in Vif prevents phosphorylation at this site, significantly impairing Vif function and viral replication
Vif	binds	ELOC	20450485, 20463065, 20532212, 20728451, 24586532	Amino acid residues Vif135-158 have the most binding to the Elongin BC complex and undergo a structural change in the presence of Elongin BC
Vif	complexes with	ELOC	14564014, 15574592, 15574593, 16303161, 17285170, 18499212, 18541215, 18562529, 18789977, 19008196, 19038776, 19218568, 20096141, 20174454, 20728451, 22190034, 22190036, 22190037, 22379088, 23621690, 25124760	HIV-1 Vif (amino acids 144-149; SLQXLA motif) interacts with cellular proteins Cul5, elongins B and C, and Rbx1 to form an Skp1-Cullin-F-box (SCF)-like complex that allows Vif to interact with APOBEC3G and induce its ubiquitination and degradation
Vif	complexes with	ELOC	22190036, 22190037, 23333304, 23988114, 24402281, 25124760, 25408426, 25901786, 25912140	HIV-1 Vif, CBF-beta, CUL5, and ELOB/C form a complex that is required for Vif-mediated downregulation of A3G and A3F. CBF-beta regulates HIV-1 infectivity only in the presence of A3G
Vif	complexes with	ELOC	22190037,	CUL5/RBX2/ELOB/ELOC/Vif/CBF-beta complex catalyzes polyubiquitin chain formation on A3G in the presence of ubiquitin E2 UBE2R1 (CDC34) or UBCH5b (UBE2D2)
Vif	complexes with	ELOC	23333304,	The substitution of Leu64 or Ile66 with serine abolishes the ability of CBF-beta to interact with the Vif-EloB/EloC complex, while the substitution of Thr68 or Tyr69 with alanine has an intermediate effect on the interaction of CBF-beta with the complex
Vif	complexes with	ELOC	24402277, 24402281, 25408426	An overall crystal structure indicates that the Vif-CBF-beta-CUL5-ELOB-ELOC complex has a U-shape architecture, including the two straight arms Vif-CBF-beta and CUL5 and the bent arm formation between ELOC and CUL5 and Vif interactions
Vif	complexes with	ELOC	24402281,	Simultaneous substitution of the three Vif-interacting residues L52, W53, and D55 and the two ELOC-interacting residues P41 and H48 in CUL5 impairs the ability of CUL5 to interact with the Vif-CBF-beta-ELOB-ELOC protein complex
Vif	complexes with	ELOC	24402281,	The absence of Vif-CBF-beta reduces the interaction between the CUL5 and the EloC-EloB complex, indicating that the former two proteins have a critical role in promoting assembly of the pentameric complex
Vif	complexes with	ELOC	25981045, 27375898	HIV-1 complexes with TCEB1 (ELOC)
Vif	enhanced by	ELOC	23333304, 23988114	The solubility of HIV-1 Vif is significantly enhanced by co-expression of EloB, EloC, and CBF-beta in vitro
Vif	interacts with	ELOC	18499212, 19008196, 19038776, 20096141, 20174454, 23988114, 25124760	Mutations in HIV-1 Vif PPLP motif (amino acids 161-164) reduces Vif binding to A3G without affecting its interaction with ElonginC and Cullin5
Vif	interacts with	ELOC	22379088, 23378232, 23405965	A potent small molecular compound VEC-5 protects APOBEC3G, APOBEC3F, and APOBEC3C from HIV-1 Vif-induced degradation and enhances A3G incorporation into HIV-1 virions by inhibiting the interaction between Vif and elongin C
Vif	interacts with	ELOC	23988114,	The interaction of HIV-1 Vif with EloB/EloC complex is important for the binding of Vif to CBF-beta in cells. The Vif SOCS box mutant (SLQ to AAA) significantly disrupt its interaction with the EloB/EloC complex
Vif	interacts with	ELOC	24225024,	NMR solution structure indicates that HIV-1 Vif residues L145, A149, and L150 and ELOC residues A99 and L103 are involved in the interaction between Vif and ELOC
Vif	interacts with	ELOC	24402281,	Mutagenesis analysis indicates that HIV-1 Vif residues T123, R127, L145, and A149 and ELOC residues A100, L101, and L104 are involved in the interaction between Vif and ELOC
Vif	interacts with	ELOC	25901786,	ASK1 inhibits the interaction of HIV-1 Vif with ELOB/C in a dose-dependent manner, whereas no significant change is observed in the binding of Vif with CUL5 or CBFbeta
Vif	interacts with	ELOC	25901786,	ASK1 markedly inhibits HIV-1 Vif-induced ubiquitination of APOBEC3G by a reduction in the Vif-ELOC interaction
Vif	interacts with	ELOC	26241003,	HIV-1 Vif interacts with TCEB1 (Elongin C) as demonstrated by co-immunoprecipitation assay
Nef	interacts with	ZYX	22721673,	Yeast two hybrid assay identifies the HIV-1 Nef-interacting protein zyxin. HIV-1 Nef co-localizes with zyxin in the cortical regions of human podocyte
HIV-1 virus replication	enhanced by expression of human gene	ZYX	18854154,	Knockdown of zyxin (ZYX) by siRNA inhibits the early stages of HIV-1 replication in 293T cells infected with VSV-G pseudotyped HIV-1
retropepsin	cleaves	PPBP	10419831,	Connective tissue-activating peptide (CTAPIII) and neutrophil-activating peptide 2 (NAP/2) are generated by digestion of a ubiquitin-CTAPIII conjugate with yeast ubiquitin C-terminal hydrolase (YUH1) and HIV protease, respectively
Nef	incorporates	PPBP	27211553,	HIV-1 Nef specifically incorporates CSF2, PPBP (NAP2), CCL5, TNF, FAS, CXCL1, IL12B, MIF and OSM into plasma extracellular vesicles from HIV-1 infected patient samples
Envelope surface glycoprotein gp120	inhibited by	GSN	23575248,	Gelsolin overexpression impairs HIV-1 gp120-induced cortical F-actin reorganization and capping and gp120-mediated CD4-CCR5 and CD4-CXCR4 redistribution in permissive lymphocytes
Tat	downregulates	GSN	16526095,	In Jurkat cells expressing HIV-1 Tat, decreased expression levels are found for basic cytoskeletal proteins such as actin, beta-tubulin, annexin, cofilin, gelsolin, and Rac/Rho-GDI complex
Vpr	inhibited by	GSN	17254575,	The G5 domain of gelsolin inhibits HIV-Vpr-induced T-cell apoptosis by blocking the interaction between Vpr and VDAC
Vpr	upregulates	GSN	23874603,	A stable-isotope labeling by amino acids in cell culture coupled with mass spectrometry-based proteomics identifies upregulation of gelsolin (GSN) expression by HIV-1 Vpr in Vpr transduced macrophages
HIV-1 virus replication	enhanced by expression of human gene	GSN	23575248,	Knockdown of gelsolin by siRNA inhibits early HIV-1 infection and HIV-1 Env (gp120/gp41)-mediated membrane fusion in permissive lymphocytes
Envelope surface glycoprotein gp120	downregulates	LGALS3BP	24156545,	The expression of 90K/LGALS3BP downregulates the relative amounts of mature gp120/gp41, whereas it upregulates the relative levels of uncleaved gp160 precursor inhibiting incorporation of the viral gp120/gp41 glycoproteins into progeny virions

Envelope surface glycoprotein gp120	inhibited by	LGALS3BP	24156545,	The two central protein-binding domains (residues 127-409) of 90K/LGALS3BP are required for inhibition of gp160 processing and incorporation of the viral gp120/gp41 glycoproteins into progeny virions
Envelope surface glycoprotein gp160, precursor	inhibited by	LGALS3BP	24156545,	The two central protein-binding domains (residues 127-409) of 90K/LGALS3BP are required for inhibition of gp160 processing and incorporation of the viral gp120/gp41 glycoproteins into progeny virions
Envelope surface glycoprotein gp160, precursor	upregulates	LGALS3BP	24156545,	The expression of 90K/LGALS3BP downregulates the relative amounts of mature gp120/gp41, whereas it upregulates the relative levels of uncleaved gp160 precursor inhibiting incorporation of the viral gp120/gp41 glycoproteins into progeny virions
Envelope transmembrane glycoprotein gp41	downregulates	LGALS3BP	24156545,	The expression of 90K/LGALS3BP downregulates the relative amounts of mature gp120/gp41, whereas it upregulates the relative levels of uncleaved gp160 precursor inhibiting incorporation of the viral gp120/gp41 glycoproteins into progeny virions
Envelope transmembrane glycoprotein gp41	inhibited by	LGALS3BP	23156545,	The two central protein-binding domains (residues 127-409) of 90K/LGALS3BP are required for inhibition of gp160 processing and incorporation of the viral gp120/gp41 glycoproteins into progeny virions
Pr55(Gag)	binds	LGALS3BP	27604950,	HIV-1 Gag binds to LGALS3BP (M2BP)
Pr55(Gag)	inhibited by	LGALS3BP	27604950,	HIV-1 Gag trafficking to the plasma membrane is inhibited by LGALS3BP (M2BP) and the inhibition by LGALS3BP is dependent upon vimentin
Pol	interacts with	LGALS3BP	22190034,	HIV-1 Pol is identified to have a physical interaction with lectin, galactoside-binding, soluble, 3 binding protein (LGALS3BP) in human HEK293 and/or Jurkat cell lines by using affinity tagging and purification mass spectrometry analyses
HIV-1 virus replication	inhibited by expression of human gene	LGALS3BP	24156545,	Knockdown of 90K/LGALS3BP by siRNA enhances replication and infectivity of HIV-1 in TZM-bl cells and primary macrophages
HIV-1 virus replication	inhibited by expression of human gene	LGALS3BP	27604950,	HIV-1 replication is inhibited by LGALS3BP (M2BP) overexpression in 293HEK cells
integrase	degraded by	PSMA1	10893419,	Proteasomal degradation of HIV-1 integrase in mammalian cells occurs by the N-end rule pathway
Tat	enhances	PSMA1	9079628,	HIV-1 Tat slightly enhances the activity of the purified 26 S proteasome
Tat	inhibits	PSMA1	14550573,	HIV-1 Tat binds to the alpha2, alpha4, alpha6, alpha7, beta1, beta2, beta3, beta5, beta6, beta7, LMP7/beta5i, and MECL1/beta2i subunits of the proteasome 20 S core structure and can inhibit cellular proteasome function
Tat	inhibits	PSMA1	9079628, 12419264, 14550573	HIV-1 Tat inhibits the peptidase activity of the 20 S proteasome and interferes with the formation of the 20 S proteasome-11 S regulator complex
Tat	interacts with	PSMA1	12419264,	Amino acids Lys51, Arg52, and Asp67 of HIV-1 Tat represent the proteasome binding site of Tat, and Tat amino acids 37-72 are necessary for proteasomal interaction and suppression of 11 S regulator-mediated antigen presentation
Vif	interacts with	PSMA1	9811770, 9846577, 12167863, 12719574, 12750511, 12808465, 12808466, 12809610, 12830140, 12840737, 12859895, 12914693, 12920286, 12970355, 14527406, 14528300, 14528301, 14557625, 14564014, 14614829	HIV-1 Vif binds to the cellular cytidine deaminase APOBEC3G and targets it for degradation through an interaction with the proteasome, thereby inhibiting APOBEC3G mediated restriction of HIV-1 replication
HIV-1 virus replication	enhanced by expression of human gene	PSMA1	18854154,	Knockdown of proteasome (prosome, macropain) subunit, alpha type, 1 (PSMA1) by siRNA inhibits the early stages of HIV-1 replication in 293T cells infected with VSV-G pseudotyped HIV-1
Nef	interacts with	ZYX	22721673,	Yeast two hybrid assay identifies the HIV-1 Nef-interacting protein zyxin. HIV-1 Nef co-localizes with zyxin in the cortical regions of human podocyte
HIV-1 virus replication	enhanced by expression of human gene	ZYX	18854154,	Knockdown of zyxin (ZYX) by siRNA inhibits the early stages of HIV-1 replication in 293T cells infected with VSV-G pseudotyped HIV-1
Tat	enhances	ECM1	24742657,	HIV-1 Tat enhances adhesion of human U937 monocyte-like cells to proteins of the extracellular matrix, such as collagen IV, laminin, and ECM1
Tat	enhances	ECM1	24742657,	Treatment with cannabinoids inhibits HIV-1 Tat-enhanced attachment of U937 cells to collagen IV, laminin, or ECM1 proteins, which is linked to the cannabinoid receptor type 2 and the modulation of beta1-integrin and actin distribution
