# Supplementary material for: Dynamics of the blood plasma proteome during hyperacute HIV-1 infection
Source: Nat Commun. 2024 Dec 5;15:10593. doi: 10.1038/s41467-024-54848-0 (PMC11618498; doi:10.1038/s41467-024-54848-0)
Supplement: Supplementary file 4 — Supplementary Data 2 [file 41467_2024_54848_MOESM4_ESM.pdf]

HIV-1_Prot_Name	Keyword	Human_GeneSymbol	Human_Prot_Name	PMID(s)	Interaction_Desc
Envelope surface glycoprotein gp120	complexes with	RAN	GTP-binding nuclear protein Ran isoform 1	23125841,	Tandem affinity purification and mass spectrometry analysis identify GTP binding protein RAN (RanGTP), HIV-1 Gag, Gag/Pol, gp120, and Nef incorporated into staufen1 RNP complexes isolated from HIV-1-expressing cells
Pr55(Gag)	complexes with	RAN	GTP-binding nuclear protein Ran isoform 1	23125841,	Tandem affinity purification and mass spectrometry analysis identify GTP binding protein RAN (RanGTP), HIV-1 Gag, Gag/Pol, gp120, and Nef incorporated into staufen1 RNP complexes isolated from HIV-1-expressing cells
Pr55(Gag)	regulated by	RAN	GTP-binding nuclear protein Ran isoform 1	9562972,	Ran terminates the nuclear import of the Matrix protein of HIV-1 Gag by directly binding to karyopherin beta and disassembling the import complex
capsid	inhibited by	RAN	GTP-binding nuclear protein Ran isoform 1	23097435,	RanGTP inhibits the ability of TNPO3 to stimulate the uncoating of HIV-1 CA cores
matrix	regulated by	RAN	GTP-binding nuclear protein Ran isoform 1	9562972,	Ran terminates the nuclear import of HIV-1 Matrix by directly binding to karyopherin beta and disassembling the import complex
Gag-Pol	complexes with	RAN	GTP-binding nuclear protein Ran isoform 1	23125841,	Tandem affinity purification and mass spectrometry analysis identify GTP binding protein RAN (RanGTP), HIV-1 Gag, Gag/Pol, gp120, and Nef incorporated into staufen1 RNP complexes isolated from HIV-1-expressing cells
integrase	inhibited by	RAN	GTP-binding nuclear protein Ran isoform 1	23878195,	The binding between HIV-1 IN and TNPO3 is inhibited by RanGTP in a dose-dependent manner, leading to a TNPO3-RanGTP complex formation
Nef	complexes with	RAN	GTP-binding nuclear protein Ran isoform 1	23125841,	Tandem affinity purification and mass spectrometry analysis identify GTP binding protein RAN (RanGTP), HIV-1 Gag, Gag/Pol, gp120, and Nef incorporated into staufen1 RNP complexes isolated from HIV-1-expressing cells
Rev	binds	RAN	GTP-binding nuclear protein Ran isoform 1	25486595,	A dimeric CRM1-RanGTP complex binds a Rev-RRE complex to export the Rev-RRE complex from the nuclear to the cytoplasm in cells
Rev	binds	RAN	GTP-binding nuclear protein Ran isoform 1	9837918, 10518602, 18508616, 19149559, 21358275, 22355797, 22783232, 24530126, 25486594, 25486595, 25564443, 25723178	the GTP bound form of Ran (RanGTP) binds to a preformed Rev-CRM1 (exportin 1) complex to mediate nuclear export of HIV-1 mRNA
Rev	binds	RAN	GTP-binding nuclear protein Ran isoform 1	9837918, 18508616, 19149559, 22783232	binding of the GTP bound form of Ran (RanGTP) to a preformed Rev-CRM1 complex is linked to an interaction of RanGTP with the nuclear export signal (NES) of Rev (amino acids 75-83)
Rev	enhanced by	RAN	GTP-binding nuclear protein Ran isoform 1	12134013, 18508616	Rev-Rev interactions (multimerization) are enhanced by RanGTP
Rev	interacts with	RAN	GTP-binding nuclear protein Ran isoform 1	22174317,	HIV-1 Rev interacting protein, RAN, is identified by the in-vitro binding experiments involving cytosolic or nuclear extracts from HeLa cells
Tat	interacts with	RAN	GTP-binding nuclear protein Ran isoform 1	19454010,	Interaction of HIV-1 Tat with RAN in T-cells is identified by a proteomic strategy based on affinity chromatography
