# Supplementary material for: Dynamics of the blood plasma proteome during hyperacute HIV-1 infection
Source: Nat Commun. 2024 Dec 5;15:10593. doi: 10.1038/s41467-024-54848-0 (PMC11618498; doi:10.1038/s41467-024-54848-0)
Supplement: Supplementary file 5 — Supplementary Data 3 [file 41467_2024_54848_MOESM5_ESM.pdf]

HIV-1_Prot_Name	Keyword	Human_GeneSymbol	PMID(s)	Interaction_Desc
Envelope surface glycoprotein gp120	activates	PRKCB	19363595,	c-FLIPL inhibits Bax activation via modulating PKC expression at the transcriptional level involving AP-2 during gp120 treatment
Envelope surface glycoprotein gp120	complexes with	HSPA8	23125841,	Tandem affinity purification and mass spectrometry analysis identify heat shock 70kDa protein 8 (HSPA8, HSC70), HIV-1 Gag, Gag/Pol, gp120, and Nef incorporated into staufen1 RNP complexes isolated from HIV-1-expressing cells
Envelope surface glycoprotein gp120	inhibited by	HSPA8	12832005,	Over expression of hsp70 with a herpes viral amplicon vector protects cultured hippocampal rat neurons from gp120 neurotoxicity
Envelope surface glycoprotein gp120	interacts with	ITGB3	23152803,	HIV-1 Tat complexes with gp120 to induce entry of VLPs expressing R5- or X4-tropic Env into MDDCs, which involves alpha5beta1, alpha5beta3, and alpha5beta5 integrins
Envelope surface glycoprotein gp120	interacts with	PSMB6	22190034,	HIV-1 gp120 is identified to have a physical interaction with proteasome subunit, beta type, 6 (PSMB6) in human HEK293 and/or Jurkat cell lines by using affinity tagging and purification mass spectrometry analyses
Envelope surface glycoprotein gp120	interacts with	PRKCB	11141237, 11504923	Induction of apoptosis in cell cultures through binding of HIV-1 gp120 or gp160 to CXCR4 involves protein kinase C, basic fibroblast growth factor, caspase-3, and the pro-apoptotic molecule Bax
Envelope surface glycoprotein gp120	interacts with	PRKCB	1970444,	Down modulation of the interaction between HIV-1 gp120 and CD4 by TPA is blocked by protein kinase C (PKC) inhibitors, suggesting PKC may play an important role in HIV-1 infection
Envelope surface glycoprotein gp120	interacts with	PRKCB	22114277,	HIV-1 gp120 activates forward trafficking and surface clustering of NMDA receptors in membrane micro domains by a PKA-dependent phosphorylation of the NR1 C-terminal Ser897, followed by a PKC-dependent phosphorylation of Ser896
Envelope surface glycoprotein gp120	interacts with	PRKCB	3259291,	A specific interaction between CD4 and HIV-1 gp120 is required for phosphorylation of CD4, which could involve protein kinase C
Envelope surface glycoprotein gp120	interacts with	PRKCB	8599832,	IL-16 induces rapid translocation of PKC from the cytosol to the membrane in CD4+ cells; PKC inhibitors completely block IL-16-induced lymphocyte migration as well as the motile response induced by HIV-1 gp120 and anti-CD4 antibody binding to CD4
Envelope surface glycoprotein gp120	interacts with	TXNDC5	22190034,	HIV-1 gp120 is identified to have a physical interaction with thioredoxin domain containing 5 (TXNDC5) in human HEK293 and/or Jurkat cell lines by using affinity tagging and purification mass spectrometry analyses
Envelope surface glycoprotein gp120	regulated by	PRKCB	15689238,	Pre-treatment of endothelial cells with fibroblast growth factor 2 (FGF2) protects cells from HIV-1 gp120 angiotoxicity; this protection is regulated by crosstalk among the ERK, PI3K-AKT and PKC signaling pathways
Envelope surface glycoprotein gp120	regulated by	PRKCB	18632858, 20842205	PKC-dependent pathway, particularly PKCalpha and PKCbeta1, requires HIV-1 gp120-mediated Rac-1 activation and membrane fusion
Envelope surface glycoprotein gp120	upregulates	HSPA8	7906708,	The exposure of permissive CD4+ cells to HIV-1 gp120 increases the synthesis and nuclear translocation of 70kDa heat shock protein
Envelope surface glycoprotein gp120	upregulates	PRKCB	8206685,	HIV-1 gp120 increases the levels of calcium-dependent and -independent PKC isozymes; the most striking change is observed in PKC-zeta isozyme levels
Envelope surface glycoprotein gp160, precursor	interacts with	UBB	27375898,	HIV-1 gp160 interacts with UBB
Envelope surface glycoprotein gp160, precursor	interacts with	PSMB6	22190034,	HIV-1 gp160 is identified to have a physical interaction with proteasome subunit, beta type, 6 (PSMB6) in human HEK293 and/or Jurkat cell lines by using affinity tagging and purification mass spectrometry analyses
Envelope surface glycoprotein gp160, precursor	interacts with	PRKCB	7642615,	HIV-1 gp160-induced AP-1 complex formation is dependent upon protein tyrosine phosphorylation and is abolished by inhibitors of protein kinase C, but it is unaffected by calcium channel blockers or cyclosporine A
Envelope surface glycoprotein gp160, precursor	interacts with	TXNDC5	27375898,	HIV-1 gp160 interacts with TXNDC5; predicted interaction to be within the endoplasmic reticulum and function as a thioredoxin reductase
Envelope surface glycoprotein gp160, precursor	relocalizes	PRKCB	11744714,	HIV-1 gp160-induced Ca(2+) influx reduction is antagonized by an inhibitor acting especially on PKC alpha and PKC beta I; Western blotting analyses show that the cellular distribution of PKC alpha and -beta I are significantly modified by gp160

Envelope transmembrane glycoprotein gp41	inhibits	PRKCB	1832084, 2139676	A synthetic peptide containing residues 581-597 from HIV-1 gp41 inhibits protein kinase C (pkC)-mediated phosphorylation of the CD3 gamma-chain in intact cells and directly inhibits partially purified pkC
Envelope transmembrane glycoprotein gp41	inhibits	PRKCB	7850771,	A synthetic peptide corresponding to cytoplasmic domain residues 828-848 of HIV-1 gp41 inhibits pKC-catalysed phosphorylation of a protein substrate
Envelope transmembrane glycoprotein gp41	interacts with	TXNDC5	22190034,	HIV-1 gp41 is identified to have a physical interaction with thioredoxin domain containing 5 (TXNDC5) in human HEK293 and/or Jurkat cell lines by using affinity tagging and purification mass spectrometry analyses
Pr55(Gag)	complexes with	HSPA8	23125841,	Tandem affinity purification and mass spectrometry analysis identify heat shock 70kDa protein 8 (HSPA8, HSC70), HIV-1 Gag, Gag/Pol, gp120, and Nef incorporated into staufen1 RNP complexes isolated from HIV-1-expressing cells
Pr55(Gag)	incorporates	HSPA8	11932435, 21738476	Hsc70 is specifically incorporated into HIV-1 virions, similar to Hsp70 which is incorporated into virions through an interaction with HIV-1 Gag
capsid	interacts with	PPIF	25505242,	The interaction of HIV-1 CA with human cellular peptidylprolyl isomerase F protein (PPIF, cyclophilin F) is identified by yeast two-hybrid screen
capsid	ubiquitinated by	UBB	15994808, 16775314	Monoubiquitinated forms of the HIV-1 matrix (MA), capsid (CA), and nucleocapsid (NC) proteins are detected in mature virus particles
matrix	phosphorylated by	PRKCB	7876252, 8473314, 9151826, 21651489	Protein kinase C (PKC) phosphorylates HIV-1 Matrix on serine residue 111 resulting in a shift in localization of Matrix from the cytosol to the cellular membrane, suggesting a myristoyl-protein switch regulated by PKC phosphorylation
matrix	stimulated by	HSPA8	10964507,	Hsp70 facilitates nuclear import of HIV-1 preintegration complexes by stimulating the binding of HIV-1 Matrix to karyopherin alpha
matrix	ubiquitinated by	UBB	15994808, 16775314	Monoubiquitinated forms of the HIV-1 matrix (MA), capsid (CA), and nucleocapsid (NC) proteins are detected in mature virus particles
nucleocapsid	ubiquitinated by	UBB	15994808, 16775314	Monoubiquitinated forms of the HIV-1 matrix (MA), capsid (CA), and nucleocapsid (NC) proteins are detected in mature virus particles
nucleocapsid	upregulates	ITGB3	18051367,	HIV-1 NC upregulates integrin, beta 3 (platelet glycoprotein IIIa, antigen CD61) in HEK 293T cells
p1	ubiquitinated by	UBB	16775314,	HIV-1 p1 is mono- or di-ubiquitinated at levels comparable to those of the other HIV-2 Gag domains MA, CA, NC, and p6; cumulative replacement of all lysine residues in NC and p1 or in NC and p6 results in an accumulation of late budding structures
p6	ubiquitinated by	UBB	11087859, 12610113, 15994808, 16808324	Ubiquitination of HIV-1 p6-Gag may be important for proper virus release from cells
p6	ubiquitinated by	UBB	17609272,	HIV-1 Gag PTAP mutant impairs the entry of Gag into the ESCRT pathway and leads to increased ubiquitination of Gag
p6	ubiquitinated by	UBB	9525617, 11087859, 11087860, 11112487, 11991975, 16775314	The L domain (P(T/S)APP; amino acids 7-11) of HIV-1 p6-Gag interacts with a ubiquitin ligase complex resulting in the monoubiquitination of p6
Gag-Pol	complexes with	HSPA8	23125841,	Tandem affinity purification and mass spectrometry analysis identify heat shock 70kDa protein 8 (HSPA8, HSC70), HIV-1 Gag, Gag/Pol, gp120, and Nef incorporated into staufen1 RNP complexes isolated from HIV-1-expressing cells
integrase	degraded by	PSMB6	10893419,	Proteasomal degradation of HIV-1 integrase in mammalian cells occurs by the N-end rule pathway
retropepsin	inhibited by	PRKCB	10491200,	Phosphorylation of human recombinant vimentin by PKC inhibits the proteolytic processing of the vimentin head domain by HIV-1 protease
reverse transcriptase	phosphorylated by	PRKCB	10641798,	HIV-1 RT heterodimer expressed in bacteria can be phosphorylated in vitro by several purified mammalian protein kinases including auto-activated protein kinase (PK), CKII, cytosolic protamine kinase (CPK), myelin basic protein kinase 1 (MBPK1), and PRKC
Nef	co-localizes with	HSPA8	19912576,	HIV-1 Nef increases the production of exosomes and co-localizes with exosomal proteins CD63, AIP/Alix, AChE, Hsc70, LAMP2, and annexin A2 in HeLa cells

Nef	complexes with	HSPA8	23125841,	Tandem affinity purification and mass spectrometry analysis identify heat shock 70kDa protein 8 (HSPA8, HSC70), HIV-1 Gag, Gag/Pol, gp120, and Nef incorporated into staufen1 RNP complexes isolated from HIV-1-expressing cells
Nef	downregulates	PRKCB	10417813, 17632570	HIV-1 Nef selectively downregulates beta II and epsilon PKC isoforms in human astrocytoma cells
Nef	interacts with	HSPA8	21763498,	Heat shock proteins Hsp40 and Hsp70 interact with HIV-1 Nef and form a complex in cells
Rev	ubiquitinated by	UBB	17067581,	HIV-1 Rev is modified by polyubiquitination at Lys-33 and Lys-115; Lys-33 is the most efficient residue for branching of ubiquitin chains
Tat	activates	PRKCB	15488737, 18692180	HIV-1 Tat activates the activity of PKC beta(II), which is essential for the activation of IL-10 production in human monocytes and macrophages
Tat	activates	PRKCB	18692180,	HIV-1 Tat-mediated stimulation of IL-10 production through the activation of PKC beta(II), but not TNF-alpha, requires p38 MAP kinase in human macrophages
Tat	activates	PRKCB	18692180,	HIV-1 Tat activates the activity of PKC beta(II), which is essential for the activation of TNF-alpha production in human macrophages
Tat	activates	PRKCB	18692180,	HIV-1 Tat-mediated stimulation of IL-10 and TNF-alpha production through the activation of PKC beta(II) requires ERK1/2 MAP kinase and NF-kappaB transcription factor in human macrophages
Tat	activates	PRKCB	27053552,	HIV-1 Tat protein activates RELA (p65), MAP kinases ERK1/2 and p38, and PKC-bII in a TLR4-dependent manner in human monocytes
Tat	activates	PRKCB	8627654, 9446795, 9671211, 10843712, 11044099, 11154208, 11833470, 11919157, 12482669, 15488737, 18541226, 27053552	HIV-1 Tat activates protein kinase C, resulting in the induction of TNF-alpha, IL-6 and IL-10 expression and the secretion of MCP-1
Tat	binds	ITGB3	2202737, 7539135, 7682219, 7690138, 9517988, 10397733, 21951552	The arginine-glycine-aspartic acid (RGD) sequence present at the carboxy-terminal of HIV-1 Tat mediates vascular cell and monocyte migration and invasion by binding to the alpha-5-beta-1 and alpha-v-beta-3 integrins
Tat	binds	PSMB6	14550573,	HIV-1 Tat binds to the alpha2, alpha4, alpha6, alpha7, beta1, beta2, beta3, beta5, beta6, beta7, LMP7/beta5i, and MECL1/beta2i subunits of the proteasome 20 S core structure and can inhibit cellular proteasome function
Tat	cooperates with	ITGB3	20661303,	HIV-1 Tat-mediated inhibition of autophagy in bystander macrophages/monocytic cells requires CXCR4, VEGFR1, and beta-integrins
Tat	cooperates with	ITGB3	21143381,	HIV-1 Tat-induced platelet activation requires the chemokine receptor CCR3 and beta3-integrin expression on platelets and calcium flux in platelets
Tat	cooperates with	PRKCB	20336759,	Phospholipase C/protein kinase C signaling pathway-dependent phosphorylation of p44/42 and JNK MAP kinases participates partially in IL-1beta induction by TAT
Tat	enhances	PSMB6	9079628,	HIV-1 Tat slightly enhances the activity of the purified 26 S proteasome
Tat	inhibits	PSMB6	9079628, 12419264, 14550573	HIV-1 Tat inhibits the peptidase activity of the 20 S proteasome and interferes with the formation of the 20 S proteasome-11 S regulator complex
Tat	interacts with	HSPA8	25496916,	Heat shock 70kDa protein 8 (HSPA8) is identified to interact with HIV-1 Tat mutant Nullbasic in HeLa cells by LC MS/MS
Tat	interacts with	ITGB3	16105876, 16166568	HIV-1 Tat exerts several pleiotropic effects by interacting with different cellular receptors, including integrin alpha(v)beta3, which triggers the activation of focal adhesion kinase, RhoA and pp60src
Tat	interacts with	ITGB3	22362758,	Endothelial cell adherent to HIV-1 Tat induces rearrangement of actin cytoskeleton and is dependent on integrin alpha5beta3
Tat	interacts with	ITGB3	22528484,	Sialic acid (NeuAc)-binding lectin from Maakia amurensis binds the NeuAc residues of integrin alpha5beta3 and inhibits the interaction of the integrin with the basic domain of HIV-1 Tat

Tat	interacts with	ITGB3	22528484,	Sialic acid is required for signal transduction triggered by HIV-1 Tat/integrin alpha5beta3 interaction in endothelial cells
Tat	interacts with	ITGB3	23152803,	HIV-1 Tat complexes with gp120 to induce entry of VLPs expressing R5- or X4-tropic Env into MDDCs, which involves alpha5beta1, alpha5beta3, and alpha5beta5 integrins
Tat	interacts with	ITGB3	25313583,	HIV-1 Tat-induced inhibition of IFN-gamma release is regulated by the interaction of Tat-RGD domain with alpha5beta1 and alpha5beta3 integrins in CD8+ T cells
Tat	interacts with	ITGB3	7539135, 7690138, 10397733, 10438928	HIV-1 Tat induces angiogenesis and cooperates in the development of AIDS-associated Kaposi sarcoma as a result of interactions with integrins alpha-5-beta-1 and alpha-v-beta 3
Tat	interacts with	ITGB3	8757599,	HIV-1 Tat induced monocyte invasion is inhibited by anti-beta integrin Ab or tissue inhibitor of metalloproteinase (TIMP), indicating an interaction with beta integrins and TIMP
Tat	interacts with	ITGB3	9256940, 11867691	The RGD-containing domain of exogenous HIV-1 Tat inhibits the engulfment of apoptotic bodies by dendritic cells through an interaction with integrin alpha v beta 3
Tat	interacts with	ITGB3	9916748,	IFN-gamma interacts with HIV-1 Tat to induce endothelial cells to proliferate and invade the extracellular matrix by upregulating the receptors for Tat (integrins alpha-5-beta-1 and alpha-v-beta-3), suggesting Tat and IFN-gamma play major roles in AIDS-KS
Tat	interacts with	PSMB6	12419264,	Amino acids Lys51, Arg52, and Asp67 of HIV-1 Tat represent the proteasome binding site of Tat, and Tat amino acids 37-72 are necessary for proteasomal interaction and suppression of 11 S regulator-mediated antigen presentation
Tat	phosphorylated by	PRKCB	8914829, 21651489	Protein kinase C phosphorylates HIV-1 Tat on serine residue 46
Tat	regulated by	HSPA8	10617616,	Hsp70 and Hsp90 and Cdc37 regulate the stabilization and folding of CDK9 as well as the assembly of an active CDK9/cyclin T1 complex responsible for P-TEFb-mediated HIV-1 Tat transactivation
Tat	regulated by	PRKCB	2182321, 18577246	Protein kinase C is required for HIV-1 Tat-mediated transactivation of the viral LTR promoter, indicating protein kinase C regulates the process of HIV-1 transactivation and may play a role in the transition of HIV from latency to productive growth
Tat	ubiquitinated by	UBB	12883554,	HIV-1 Tat is ubiquitinated on Lys71 by Hdm2 in conjunction with ubiquitin-activating enzyme E1 and ubiquitin-conjugating enzyme E2D 1 (UbcH5)
Tat	upregulates	CLIC1	23166591,	Expression of HIV-1 Tat upregulates the abundance of chloride intracellular channel 1 (CLIC1) in the nucleoli of Jurkat T-cells
Tat	upregulates	C7	24667918,	Microarray analysis indicates HIV-1 Tat-induced upregulation of complement component 7 (C7) in primary human brain microvascular endothelial cells
Tat	upregulates	ITGB3	16339753,	HIV-1 Tat upregulates the levels of CD61 in phorbol myristate acetate (PMA)-stimulated K562 hematopoietic progenitor cells
Tat	upregulates	ITGB3	22362758,	Endothelial cell adherent to HIV-1 Tat induces upregulation of VEGFR2, integrin beta(3) subunit, and pp60src and recruits VEGFR2, integrin beta(3) subunit, paxillin, focal adhesion kinase, and pp60src to ventral plasma membranes
Vif	inhibited by	UBB	18596088,	HIV-1 Vif-induced G2 accumulation requires a Cul5-based E3 ligase, but is independent of APOBEC3D/E, F, and G expression. Overexpression of ubiquitin(K48R) abolishes Vif-induced G2 accumulation
Vif	interacts with	HSPA8	27375898,	HIV-1 Vif interacts with HSPA8; predicted interaction to be relevant to gene regulation

Vif	interacts with	PSMB6	9811770, 9846577, 12167863, 12719574, 12750511, 12808465, 12808466, 12809610, 12830140, 12840737, 12859895, 12914693, 12920286, 12970355, 14527406, 14528300, 14528301, 14557625, 14564014, 14614829	HIV-1 Vif binds to the cellular cytidine deaminase APOBEC3G and targets it for degradation through an interaction with the proteasome, thereby inhibiting APOBEC3G mediated restriction of HIV-1 replication
Vpr	competes with	HSPA8	10964507, 19275587	HIV-1 Vpr competes with Hsp70 for binding to karyopherin alpha
Vpr	complexes with	UBB	24318982,	A di-Ub(K48)-hHR23A-Vpr ternary complex is formed with Lys-48-linked di-ubiquitin binding to the UBA1 domain in the Vpr-hHR23A complex
Vpr	cooperates with	UBB	19516896,	The C-terminal domain or the R90 residue of HIV-1 Vpr are important for Vpr-mediated IL-12 degradation via ubiquitin-dependent pathway
Vpr	downregulates	CLIC1	23874603,	A stable-isotope labeling by amino acids in cell culture coupled with mass spectrometry-based proteomics identifies downregulation of chloride intracellular channel 1 (CLIC1) expression by HIV-1 Vpr in Vpr transduced macrophages
Vpr	downregulates	RTN4	23874603,	A stable-isotope labeling by amino acids in cell culture coupled with mass spectrometry-based proteomics identifies downregulation of reticulon 4 (RTN4) expression by HIV-1 Vpr in Vpr transduced macrophages
Vpr	interacts with	HSPA8	21763498,	HIV-1 Vpr is required for the inhibitory effect of Hsp70 on viral gene expression and replication
Vpu	interacts with	RTN4	22190034,	HIV-1 Vpu is identified to have a physical interaction with reticulon 4 (RTN4) in human HEK293 and/or Jurkat cell lines by using affinity tagging and purification mass spectrometry analyses
HIV-1 virus replication	enhanced by expression of human gene	APOB	18976975,	Knockdown of apolipoprotein B (APOB) by siRNA inhibits HIV-1 replication in HeLa P4/R5 cells
HIV-1 virus replication	enhanced by expression of human gene	PSMB6	18854154,	Knockdown of proteasome (prosome, macropain) subunit, beta type, 6 (PSMB6) by siRNA inhibits the early stages of HIV-1 replication in 293T cells infected with VSV-G pseudotyped HIV-1
