# Supplementary material for: Dynamics of the blood plasma proteome during hyperacute HIV-1 infection
Source: Nat Commun. 2024 Dec 5;15:10593. doi: 10.1038/s41467-024-54848-0 (PMC11618498; doi:10.1038/s41467-024-54848-0)
Supplement: Supplementary file 6 — Supplementary Data 4 [file 41467_2024_54848_MOESM6_ESM.pdf]

Gene.Names	HIV-1_GeneID	HIV-1_Prot_Acc	HIV-1_Prot_Name	Keyword	PMID(s)	Interaction_Desc
NRP1	156110	NP_057857.2	Nef	upregulates	18443354,	HIV-1 Nef upregulates VEGFR2 and its co-receptor neuropilin-1 and downregulates the expression of semaphorin 3a in podocytes
NRP1			HIV-1 virus replication	enhanced by expression of human gene	19460752,	Knockdown of neuropilin 1 (NRP1) by shRNA library screening inhibits HIV-1 replication in cultured Jurkat T-cells
LDHA	155971	NP_579894.2	Envelope surface glycoprotein gp120	complexes with	23125841,	Tandem affinity purification and mass spectrometry analysis identify lactate dehydrogenase A (LDHA), HIV-1 Gag, Gag/Pol, gp120, and Nef incorporated into staufen1 RNP complexes isolated from HIV-1-expressing cells
LDHA	155971	NP_579894.2	Envelope surface glycoprotein gp120	induces release of	11125887,	HIV-1 gp120/41 (SFVenvBX08)-expressing microglia exhibit a 170% increase in lactate dehydrogenase (LDH) release
LDHA	155971	NP_579895.1	Envelope transmembrane glycoprotein gp41	induces release of	11582518,	A lentivirus lytic peptide 1 (LLP-1) corresponding to the carboxyl terminus of HIV-1 gp41 induces a significant lactate dehydrogenase (LDH, a marker of cell death) release from human neuronal and glial cell lines
LDHA	155030	NP_057850.1	Pr55(Gag)	complexes with	23125841,	Tandem affinity purification and mass spectrometry analysis identify lactate dehydrogenase A (LDHA), HIV-1 Gag, Gag/Pol, gp120, and Nef incorporated into staufen1 RNP complexes isolated from HIV-1-expressing cells
LDHA	155348	NP_057849.4	Gag-Pol	complexes with	23125841,	Tandem affinity purification and mass spectrometry analysis identify lactate dehydrogenase A (LDHA), HIV-1 Gag, Gag/Pol, gp120, and Nef incorporated into staufen1 RNP complexes isolated from HIV-1-expressing cells
LDHA	156110	NP_057857.2	Nef	complexes with	23125841,	Tandem affinity purification and mass spectrometry analysis identify lactate dehydrogenase A (LDHA), HIV-1 Gag, Gag/Pol, gp120, and Nef incorporated into staufen1 RNP complexes isolated from HIV-1-expressing cells
LDHA	155871	NP_057853.1	Tat	downregulates	15710247,	Upregulation of actin, heat shock protein 90 and mitochondrial single-stranded DNA binding protein, and downregulation of lactate dehydrogenase are identified in human astrocytes expressing Tat
LDHA	155871	NP_057853.1	Tat	upregulates	23025307,	HIV-1 Tat upregulates lactate dehydrogenase A (LDHA) expression in Jurkat T-cells
CP	155871	NP_057853.1	Tat	upregulates	24667918,	Microarray analysis indicates HIV-1 Tat-induced upregulation of ceruloplasmin (CP; ferroxidase) in primary human brain microvascular endothelial cells
F13A1	155871	NP_057853.1	Tat	downregulates	24667918,	Microarray analysis indicates HIV-1 Tat-induced downregulation of coagulation factor XIII, A1 polypeptide (F13A1) in primary human brain microvascular endothelial cells
CFB	155871	NP_057853.1	Tat	upregulates	24667918,	Microarray analysis indicates HIV-1 Tat-induced upregulation of complement factor B (CFB) in primary human brain microvascular endothelial cells
SERPINA3	155971	NP_579894.2	Envelope surface glycoprotein gp120	upregulates	23867815,	HIV-1 gp120-treated vaginal epithelial cells show upregulation of serpin peptidase inhibitor, clade A, member 3 (SERPINA3) expression as compared to untreated control
SERPINA3	155871	NP_057853.1	Tat	upregulates	24667918,	Microarray analysis indicates HIV-1 Tat-induced upregulation of serpin peptidase inhibitor, clade A, member 3 (SERPINA3) in primary human brain microvascular endothelial cells
A2M	155971	NP_579895.1	Envelope transmembrane glycoprotein gp41	downregulates	23383108,	A synthetic peptide corresponding to the immunosuppressive domain (amino acids 574-592) of HIV-1 gp41 downregulates the expression of alpha-2-macroglobulin (A2M) in peptide-treated PBMCs
A2M	155348	NP_705926.1	retropepsin	cleaves	1724156, 7524416, 7690356	The cleavage site of alpha 2-Macroglobulin by HIV-1 protease is the Phe684-Tyr685 bond
A2M	155871	NP_057853.1	Tat	inhibits	11100124,	Binding of HIV-1 Tat to LRP inhibits neuronal binding, uptake and degradation of physiological ligands for LRP, including alpha2-macroglobulin, apolipoprotein E4, amyloid precursor and amyloid beta-protein
C3	155971	NP_579894.2	Envelope surface glycoprotein gp120	binds	7590866, 7642209, 7893437, 7911492	Complement proteins C4, C3d, C5b-9, and properdin bind to HIV-1 gp120-coated CD4+ T cells of healthy individuals when incubated in autologous serum
C3	155971	NP_579894.2	Envelope surface glycoprotein gp120	binds	7590866, 8630395	Amino acid residues 100-129, 161-190, 231-250, 301-328, 410-449, and 470-499 of HIV-1 gp120 are involved in its binding to C3
C3	155971	NP_579894.2	Envelope surface glycoprotein gp120	binds	8630395,	A synthetic peptide covering positions 233-251 of the HIV-1 gp120 protein binds to complement proteins C3, C4, C5, C9, and properdin
C3	155971	NP_579894.2	Envelope surface glycoprotein gp120	cleaves	8471312,	Complexes of recombinant HIV-1 gp120 with anti-HIV-1 antibodies cleave C3 and present generated C3 fragments on the cell surface
C3	155971	NP_579894.2	Envelope surface glycoprotein gp120	interacts with	7535292,	Inhibition of DAF or use of factor H depleted sera significantly increases C3 deposition on recombinant HIV-1 gp120 coated CD4 cells
C3	155971	NP_579894.2	Envelope surface glycoprotein gp120	interacts with	7893437,	Preincubation of HIV-1 gp41 with either factor H or properdin, and of HIV-1 gp120 with C3b or C4b affect the interaction between HIV-1 gp41 and gp120
C3	155971	NP_057856.1	Envelope surface glycoprotein gp160, precursor	upregulates	8471312, 9544576	Complement component 3 (C3) production is upregulated by HIV-1 gp160
C3	156110	NP_057857.2	Nef	upregulates	11884542, 19878567	HIV-1 induces the upregulation of complement factor C3 in astrocytes and neurons through signaling pathways that involve protein kinase C and adenylate cyclase activation, which is an effect that may contribute to the pathogenesis of AIDS in the brain
C3	155871	NP_057853.1	Tat	upregulates	24667918,	Microarray analysis indicates HIV-1 Tat-induced upregulation of complement component 3 (C3) in primary human brain microvascular endothelial cells
C1QC	155971	NP_579894.2	Envelope surface glycoprotein gp120	binds	1875953, 7507842, 7590886, 7642209, 12396016	Free C1q binds to HIV-1 gp120; digestion of the C1q stem portion with collagenase completely eliminates its binding to recombinant gp120, suggesting that the collagen-like stem region of C1q participates in the binding to gp120
C1QC	155971	NP_579894.2	Envelope surface glycoprotein gp120	interacts with	1875953, 9443108	Fibronectin, which is present in submandibular saliva, binds to HIV-1 gp120/160 and enhances the interaction of C1q with gp120/160
C1QC	155971	NP_579895.1	Envelope transmembrane glycoprotein gp41	binds	1744579,	HIV-1 gp41 (amino acid residues 561-575, 591-605 and 601-620) binds to complement C1q and activates the C1 complex in a dose- and time-dependent manner
C1QC	155971	NP_579895.1	Envelope transmembrane glycoprotein gp41	binds	1744579, 7507842, 7739575, 8245486, 8252810, 10504397, 11318594, 12396016	Three sites (amino acids 526-538, 590-613 and 625-655) of the cell-external part of HIV-1 gp41 bind both HIV-1 gp120 and C1q
C1QC	155971	NP_579895.1	Envelope transmembrane glycoprotein gp41	interacts with	7739575,	The interaction between C1q and HIV-gp41 is dependent upon the presence of calcium; calcium can not be replaced by larger cations such as strontium, barium, lead or smaller ions such as magnesium and manganese
C1QC	155971	NP_579894.2	Envelope surface glycoprotein gp120	binds	1875953, 7507842, 7590886, 7642209, 12396016	Free C1q binds to HIV-1 gp120; digestion of the C1q stem portion with collagenase completely eliminates its binding to recombinant gp120, suggesting that the collagen-like stem region of C1q participates in the binding to gp120
C1QC	155971	NP_579894.2	Envelope surface glycoprotein gp120	interacts with	1875953, 9443108	Fibronectin, which is present in submandibular saliva, binds to HIV-1 gp120/160 and enhances the interaction of C1q with gp120/160
C1QC	155971	NP_579895.1	Envelope transmembrane glycoprotein gp41	binds	1744579,	HIV-1 gp41 (amino acid residues 561-575, 591-605 and 601-620) binds to complement C1q and activates the C1 complex in a dose- and time-dependent manner
C1QC	155971	NP_579895.1	Envelope transmembrane glycoprotein gp41	binds	1744579, 7507842, 7739575, 8245486, 8252810, 10504397, 11318594, 12396016	Three sites (amino acids 526-538, 590-613 and 625-655) of the cell-external part of HIV-1 gp41 bind both HIV-1 gp120 and C1q
C1QC	155971	NP_579895.1	Envelope transmembrane glycoprotein gp41	interacts with	7739575,	The interaction between C1q and HIV-gp41 is dependent upon the presence of calcium; calcium can not be replaced by larger cations such as strontium, barium, lead or smaller ions such as magnesium and manganese
C9	155971	NP_579894.2	Envelope surface glycoprotein gp120	binds	8630395,	A synthetic peptide covering positions 233-251 of the HIV-1 gp120 protein binds to complement proteins C3, C4, C5, C9, and properdin
APOH	155030	NP_057850.1	Pr55(Gag)	binds	8989432,	Binding of apolipoprotein H to HIV-1 Gag protein p18 as well as to the Gag p55 polyprotein has been demonstrated in vitro

FN1	155971	NP_579894.2	Envelope surface glycoprotein gp120	binds	1875953, 8173552	Fibronectin, which is present in submandibular saliva, binds to HIV-1 gp120/160 and enhances the interaction of C1q with gp120/160
FN1	155971	NP_579894.2	Envelope surface glycoprotein gp120	binds	8952048, 9443108, 10706716	HIV-1 gp120 binds to fibronectin (FN) present on the surface of platelets; the specificity of this binding is confirmed by the inhibition obtained by pretreating platelets with anti-FN antibodies
FN1	155971	NP_579894.2	Envelope surface glycoprotein gp120	induces phosphorylation of	22241990,	HIV-1 gp120 induces phosphorylation of fibronectin and enhances a physical association between fibronectin and Robo4 in human lymphatic endothelial cells
FN1	155971	NP_057856.1	Envelope surface glycoprotein gp160, precursor	binds	1875953, 9443108	HIV-1 gp160 and gp120 specifically recognize the C-terminal heparin-binding domain of fibronectin (Fn) and this binding inhibits the interaction of gp160/gp120 with soluble CD4
FN1	155971	NP_579895.1	Envelope transmembrane glycoprotein gp41	binds	8173552,	Fibronectin (FN) binds to HIV-1 glycoproteins, including gp41 and gp120; preincubation with antibodies against FN abolishes this binding
FN1	155348	NP_705926.1	retropepsin	cleaves	1959621, 8997639, 12119179	The cell-associated protein fibronectin (A-chain) is specifically cleaved in vitro by HIV-1 protease
FN1	156110	NP_057857.2	Nef	upregulates	10451539, 11180285	Exogenous HIV-1 Nef upregulates fibronectin (FN) expression in MT4 and H9 T-cell lines
FN1	155871	NP_057853.1	Tat	competes with	7690138,	HIV-1 Tat competes with fibronectin for binding to integrins
FN1	155871	NP_057853.1	Tat	modulated by	9339851, 9626063	Fibronectin modulates the effects of HIV-1 Tat on endothelial cells and murine Kaposi's sarcoma-like cells
FN1	155871	NP_057853.1	Tat	upregulates	1409674, 8599839, 11311202	HIV-1 Tat upregulates fibronectin expression in salivary gland cell lines, thymic epithelial cells, and glioblastoma cells
FN1	155971	NP_579894.2	Envelope surface glycoprotein gp120	binds	1875953, 8173552	Fibronectin, which is present in submandibular saliva, binds to HIV-1 gp120/160 and enhances the interaction of C1q with gp120/160
FN1	155971	NP_579894.2	Envelope surface glycoprotein gp120	binds	8952048, 9443108, 10706716	HIV-1 gp120 binds to fibronectin (FN) present on the surface of platelets; the specificity of this binding is confirmed by the inhibition obtained by pretreating platelets with anti-FN antibodies
FN1	155971	NP_579894.2	Envelope surface glycoprotein gp120	induces phosphorylation of	22241990,	HIV-1 gp120 induces phosphorylation of fibronectin and enhances a physical association between fibronectin and Robo4 in human lymphatic endothelial cells
FN1	155971	NP_057856.1	Envelope surface glycoprotein gp160, precursor	binds	1875953, 9443108	HIV-1 gp160 and gp120 specifically recognize the C-terminal heparin-binding domain of fibronectin (Fn) and this binding inhibits the interaction of gp160/gp120 with soluble CD4
FN1	155971	NP_579895.1	Envelope transmembrane glycoprotein gp41	binds	8173552,	Fibronectin (FN) binds to HIV-1 glycoproteins, including gp41 and gp120; preincubation with antibodies against FN abolishes this binding
FN1	155348	NP_705926.1	retropepsin	cleaves	1959621, 8997639, 12119179	The cell-associated protein fibronectin (A-chain) is specifically cleaved in vitro by HIV-1 protease
FN1	156110	NP_057857.2	Nef	upregulates	10451539, 11180285	Exogenous HIV-1 Nef upregulates fibronectin (FN) expression in MT4 and H9 T-cell lines
FN1	155871	NP_057853.1	Tat	competes with	7690138,	HIV-1 Tat competes with fibronectin for binding to integrins
FN1	155871	NP_057853.1	Tat	modulated by	9339851, 9626063	Fibronectin modulates the effects of HIV-1 Tat on endothelial cells and murine Kaposi's sarcoma-like cells
FN1	155871	NP_057853.1	Tat	upregulates	1409674, 8599839, 11311202	HIV-1 Tat upregulates fibronectin expression in salivary gland cell lines, thymic epithelial cells, and glioblastoma cells
TF	155030	NP_579880.1	capsid	co-localizes with	26623655,	HIV-1 CA colocalizes with TF in follicular dendritic cells from lymph nodes of HIV infected patients on antiretroviral therapy
TF	155030	NP_579876.2	matrix	associates with	24830293,	HIV-1 MA associates with transferrin in recycling endosomes in human vaginal epithelial cells
TF	156110	NP_057857.2	Nef	co-localizes with	23372701,	HIV-1 Nef co-localizes with CTLA-4 in early and recycling endosomes with transferrin marker protein in HeLa cells
TF	155871	NP_057853.1	Tat	upregulates	24667918,	Microarray analysis indicates HIV-1 Tat-induced upregulation of transferrin (TF) in primary human brain microvascular endothelial cells
LTF	155971	NP_579894.2	Envelope surface glycoprotein gp120	binds	9781360, 11675140, 12103434, 14717698, 15222480, 15709021, 16261253, 16928883, 18183929, 21847071	Native lactoferrin (LF) and acylated LF from milk strongly bind to the V3 domain of the HIV-1 envelope protein gp120, resulting in inhibition of the virus-cell fusion and entry of the virus in CD4+ cells
LTF	155348	NP_705927.1	reverse transcriptase	inhibited by	25445609,	A peptide derived from human lactoferricin inhibits HIV-1 RT activity in a dose-dependent manner
LTF			HIV-1 virus replication	inhibited by expression of human gene	27182834,	HIV-1 is inhibited by TNC (tenascin C), MUC1, and LTF (lactoferrin) as shown through neutralization assays in TZM-bl cells
ANG			HIV-1 virus replication	incorporates expression of human gene	27211553,	HIV-1 infected clinical samples have plasma extracellular vesicles that contain elevated CCL1 (I309), IGFBP1, CCL5 (RANTES), GMCSF, ANG (Angiogenin), ADIPOQ (ACRP30), CSF3 (GCSF), CXCL1, ICAM1, IL2RA, IL6R, TNFRSF1A, and TIMP1 compared to healthy donors
SLPI	155971	NP_579894.2	Envelope surface glycoprotein gp120	upregulates	15858026, 16928883	The expression and production of secretory leukocyte protease inhibitor (SLPI), a 12-kDa mucosal antiviral protein, can be stimulated in oral epithelial cells through interactions with HIV-1 gp120
SLPI	155348	NP_705926.1	retropepsin	enhanced by	10548568, 11170993	Secretory levels of secretory leukocyte peptidase inhibitor (SLPI) from HIV-1 infected patients is enhanced in saliva
SLPI	155348	NP_705926.1	retropepsin	inhibited by	9456660, 10598905, 10799472, 12355371, 12392704	Human secretory leukocyte protease inhibitor (hSLPI) inhibits HIV-1 infection of lymphocyte- and monocyte-derived tumor cell lines and peripheral blood lymphocytes
VWF	155971	NP_579894.2	Envelope surface glycoprotein gp120	downregulates	21612582,	HIV-1 gp120 downregulates vWF expression in human mesenchymal stem cells
VWF	155871	NP_057853.1	Tat	downregulates	22095559,	HIV-1 Tat inhibits the differentiation of mesenchymal stem cells (MSCs) to endothelial cells by downregulating the expression of VEGF-induced endothelial markers such as Flt-1, KDR and vWF
VWF			HIV-1 virus replication	enhanced by expression of human gene	18854154,	Knockdown of von Willebrand factor (VWF) by siRNA inhibits the early stages of HIV-1 replication in 293T cells infected with VSV-G pseudotyped HIV-1
VWF	155971	NP_579894.2	Envelope surface glycoprotein gp120	downregulates	21612582,	HIV-1 gp120 downregulates vWF expression in human mesenchymal stem cells
VWF	155871	NP_057853.1	Tat	downregulates	22095559,	HIV-1 Tat inhibits the differentiation of mesenchymal stem cells (MSCs) to endothelial cells by downregulating the expression of VEGF-induced endothelial markers such as Flt-1, KDR and vWF
VWF			HIV-1 virus replication	enhanced by expression of human gene	18854154,	Knockdown of von Willebrand factor (VWF) by siRNA inhibits the early stages of HIV-1 replication in 293T cells infected with VSV-G pseudotyped HIV-1
GAPDH	155971	NP_579894.2	Envelope surface glycoprotein gp120	complexes with	23125841,	Tandem affinity purification and mass spectrometry analysis identify glyceraldehyde-3-phosphate dehydrogenase (GAPDH), HIV-1 Gag, Gag/Pol, gp120, and Nef incorporated into staufen1 RNP complexes isolated from HIV-1-expressing cells
GAPDH	155030	NP_057850.1	Pr55(Gag)	complexes with	23125841,	Tandem affinity purification and mass spectrometry analysis identify glyceraldehyde-3-phosphate dehydrogenase (GAPDH), HIV-1 Gag, Gag/Pol, gp120, and Nef incorporated into staufen1 RNP complexes isolated from HIV-1-expressing cells
GAPDH	155030	NP_057850.1	Pr55(Gag)	interacts with	23237566,	Immunoprecipitation assay shows that GAPDH directly interacts with HIV-1 Gag and Gag-Pol. Packaging of LysRS and tRNA-Lys3 into virions is negatively regulated by GAPDH, leading to decreased viral infectivity
GAPDH	155348	NP_057849.4	Gag-Pol	complexes with	23125841,	Tandem affinity purification and mass spectrometry analysis identify glyceraldehyde-3-phosphate dehydrogenase (GAPDH), HIV-1 Gag, Gag/Pol, gp120, and Nef incorporated into staufen1 RNP complexes isolated from HIV-1-expressing cells
GAPDH	155348	NP_057849.4	Gag-Pol	interacts with	23237566,	Immunoprecipitation assay shows that GAPDH directly interacts with HIV-1 Gag and Gag-Pol. Packaging of LysRS and tRNA-Lys3 into virions is negatively regulated by GAPDH, leading to decreased viral infectivity
GAPDH	156110	NP_057857.2	Nef	complexes with	23125841,	Tandem affinity purification and mass spectrometry analysis identify glyceraldehyde-3-phosphate dehydrogenase (GAPDH), HIV-1 Gag, Gag/Pol, gp120, and Nef incorporated into staufen1 RNP complexes isolated from HIV-1-expressing cells
GAPDH	155908	NP_057854.1	Rev	interacts with	22174317,	HIV-1 Rev interacting protein, glyceraldehyde-3-phosphate dehydrogenase (GAPDH), is identified by the in-vitro binding experiments involving cytosolic or nuclear extracts from HeLa cells. The interaction of Rev with GAPDH is increased by RRE

GAPDH	155871	NP_057853.1	Tat	interacts with	25496916,	Glyceraldehyde-3-phosphate dehydrogenase (GAPDH) is identified to interact with HIV-1 Tat mutant Nullbasic in HeLa cells by LC MS/MS
GAPDH	155807	NP_057852.2	Vpr	downregulates	23728617,	Treatment of human primary astrocytes with HIV-1 Vpr downregulates expression of mRNA GAPDH and GAPDH activity
GAPDH	155807	NP_057852.2	Vpr	downregulates	23874603,	A stable-isotope labeling by amino acids in cell culture coupled with mass spectrometry-based proteomics identifies downregulation of glyceraldehyde-3-phosphate dehydrogenase (GAPDH) expression by HIV-1 Vpr in Vpr transduced macrophages
GAPDH			HIV-1 virus replication	inhibited by expression of human gene	23237566,	Knockdown of GAPDH by siRNA enhances HIV-1 infectivity in TZM-bl cells
HLA-A	19424028	YP_009028572.1	Asp	binds	25809376,	Two ASP peptide sequences, ASP-YL9 (89YLYNSLLQL97) and ASP-TL10 (79TPNGSIFTTL88), show high binding affinity to HLA-A*02 and HLA-B*07 molecules, respectively
HLA-A	19424028	YP_009028572.1	Asp	inhibited by	20065064, 25701112	Antisense reading frame-derived cryptic epitopes from the gag, pol, and nef genes are inhibited by the predicted HLA-I alleles, and presented by HIV-1-infected CD8+ T-cells
HLA-A	19424028	YP_009028572.1	Asp	inhibited by	25589651,	Antisense reading frame-derived cryptic epitopes from the env gene are inhibited by the HLA-I alleles in CD8+ T-cells
HLA-A	155971	NP_579894.2	Envelope surface glycoprotein gp120	complexes with	2789433, 8671651, 8877415, 9120272, 10546855, 11932387	Conformational changes in HIV-1 gp120, including an enhanced expression of the V3 loop of gp120 and of epitopes that are exposed upon CD4 binding, are consistent with the formation of a multimolecular complex between HLA class I and gp120/160
HLA-A	155971	NP_579894.2	Envelope surface glycoprotein gp120	interacts with	7539755, 9263011, 12427289	Treatment of CD4+ T cells with HIV-1 gp120 significantly increases CD4 association with CD3, CD45RA, CD45RB, CD59, CD38, CD26 and HLA class I, and decreases that with CD45RC
HLA-A	155971	NP_057856.1	Envelope surface glycoprotein gp160, precursor	binds	17116886,	Epitope Env37-46 from HIV-1 gp160 binds strongly to HLA-A3 molecules and forms very stable complexes
HLA-A	155971	NP_057856.1	Envelope surface glycoprotein gp160, precursor	interacts with	1316930, 10799863, 20200278	HIV-1 gp160-derived peptide p18 presented by H-2Dd class I major histocompatibility complex molecules is processed by angiotensin-1 converting enzyme (ACE) prior to T cell stimulation by the peptide p18
HLA-A	155971	NP_579895.1	Envelope transmembrane glycoprotein gp41	upregulates	7913356, 8084338, 9373217	HIV-1 gp41 selectively enhances MHC class I, ICAM-1, IFN-alpha, IFN-beta, and IFN-omega expression in H9 cells
HLA-A	155971	NP_579895.1	Envelope transmembrane glycoprotein gp41	upregulates	8084338,	Soluble HIV-1 gp41 can selectively enhance MHC class I and II expression on human B cells, but does not increase expression of other cell surface antigens such as CD21 and CD54 (ICAM-1)
HLA-A	155971	NP_579895.1	Envelope transmembrane glycoprotein gp41	upregulates	8084338,	Soluble HIV-1 gp41 enhancement effects on MHC class I and II antigen expression can be inhibited by soluble gp41-binding proteins of 45, 49 and 62 kD from human B cells
HLA-A	155030	NP_057850.1	Pr55(Gag)	affects	27120610,	HIV-1 p6 Gag mutation affects HLA-A antigen presentation; p6 mutation of Glu residues to Alas impairs Gag processing & virus release and enhances Gag-membrane association with increased polyubiquitation & entry of Gag into the MHC-1 presentation pathway
HLA-A	155030	NP_057850.1	Pr55(Gag)	binds	17116886,	The HIV-1 Gag 20-28 epitope binds strongly to HLA-A3 molecules and forms very stable complexes
HLA-A	155030	NP_057850.1	Pr55(Gag)	enhances	18097038,	Targeting HIV-1 Gag into the defective ribosomal product pathway enhances MHC class I antigen presentation and CD8+ T cell activation
HLA-A	155030	NP_057850.1	Pr55(Gag)	enhances	25279819,	The S40F mutation in HIV-1 p6 enhances MHC-I antigen presentation of Gag
HLA-A	155030	NP_057850.1	Pr55(Gag)	interacts with	17878955, 24942586, 25165114, 25781986	Protective HLA alleles have a true preference for HIV-1 Gag protein, while non-protective HLA alleles preferentially interact with HIV-1 Nef
HLA-A	155030	NP_057850.1	Pr55(Gag)	interacts with	21482733,	The PTAP L-domains in the p6 domain of HIV-1 Gag regulates ubiquitination of Gag which controls MHC-I presentation and gag processing in the DRiP pathway.
HLA-A	155030	NP_057850.1	Pr55(Gag)	interacts with	22826228,	The degree of HIV-1 Nef-mediated HLA-A2 downregulation strongly influences recognition of virus-infected cells by the Gag-specific CD8+ cytotoxic T lymphocyte clone
HLA-A	155030	NP_057850.1	Pr55(Gag)	upregulates	21778700,	HIV-1 Gag virus-like particles efficiently activate human monocyte-derived dendritic cells (MDDC) and induce MDDC maturation with an associated increase in the surface expression of CD80, CD86 and MHC classes I and II
HLA-A	155030	NP_579880.1	capsid	interacts with	23061377,	HLA supertypes such as HLA B*07, HLA B*58, HLA A*02 and HLA A*03 are most successful in restricting the amino acid positions of epitope dense regions of HIV-1 Nef, CA, and MA with low entropy and hydrophobic property
HLA-A	155030	NP_579876.2	matrix	interacts with	23061377,	HLA supertypes such as HLA B*07, HLA B*58, HLA A*02 and HLA A*03 are most successful in restricting the amino acid positions of epitope dense regions of HIV-1 Nef, CA, and MA with low entropy and hydrophobic property
HLA-A	155030	NP_579883.1	p6	enhances	25279819,	The S40F mutation in HIV-1 p6 enhances MHC-I antigen presentation of Gag
HLA-A	155348	NP_789740.1	Pol	binds	17116886,	The HIV-1 Pol 325-333 epitope binds strongly to HLA-A3 molecules and forms very stable complexes
HLA-A	156110	NP_057857.2	Nef	binds	15569716, 15653685	HIV-1 Nef disrupts antigen presentation by binding to MHC-I (HLA-A2) hypophosphorylated cytoplasmic tails in the endoplasmic reticulum; this Nef-MHC-I complex migrates normally into the Golgi apparatus but subsequently fails to arrive at the cell surface
HLA-A	156110	NP_057857.2	Nef	binds	17116886,	The HIV-1 Nef 73-82 epitope binds strongly to HLA-A3 molecules and forms very stable complexes
HLA-A	156110	NP_057857.2	Nef	binds	9582271, 10366557, 10982373, 11463741, 12414957, 12836198, 14965316, 15078178, 16454711, 18057255, 18073204, 18296443, 18653452, 19149577, 22301137, 22705789, 22767237, 23170180, 23202450	Four glutamic acids from position 62 to 65 in the SH3 domain of HIV-1 Nef bind to the cytoplasmic tail at position 320Y of MHC-I, and are required for the Nef-mediated downregulation of MHC-I from the cell surface
HLA-A	156110	NP_057857.2	Nef	co-localizes with	15854903,	PxxP motifs in HIV-1 Nef induce the accumulation of CCR5 in a perinuclear compartment where both molecules co-localize with MHC-1
HLA-A	156110	NP_057857.2	Nef	complexes with	21917951, 25585010	Dominant active ARF1 (Q71L) potently stabilizes interactions among AP-1 mu1, HIV-1 Nef, and HLA-A2 and that the formation of a static complex sequesters necessary trafficking components
HLA-A	156110	NP_057857.2	Nef	complexes with	22705789, 22767237	Asp327 and Tyr320 of MHC-I, Asp123 of Nef, and Arg225, Arg393, Lys396, Arg211, and Arg246 of mu 1 are involved in a crucial three-way electrostatic network, which results in the Nef-MHC-I CD-mu 1 complex formation
HLA-A	156110	NP_057857.2	Nef	degrades	18725938, 20622010	MHC-I is found in the Rab7(+) vesicles and targeted for degradation via the activity of the Nef-interacting protein, beta-COP
HLA-A	156110	NP_057857.2	Nef	downregulates	10403641, 18155264, 23170180, 23202450	HIV-1 selectively downregulates HLA-A and HLA-B but does not significantly affect HLA-C or HLA-E, which allows HIV-infected cells to avoid NK cell-mediated lysis; this effect is likely mediated by the HIV-1 Nef protein
HLA-A	156110	NP_057857.2	Nef	downregulates	10684310, 15078178, 18073204, 22301137, 22705789, 22767237, 26319395, 26607225	A methionine residue at amino acid 20 in the alpha-helix domain of HIV-1 Nef is required for the ability of Nef to downregulate MHC-I expression but not for the downregulation of CD4
HLA-A	156110	NP_057857.2	Nef	downregulates	10707087, 18005690, 19149577, 20622010	HIV-1 Nef-induced downregulation of MHC-I expression and MHC-I targeting to the trans-Golgi network (TGN) require the binding of Nef to PACS-1, a molecule that controls the TGN localization of the cellular protein furin
HLA-A	156110	NP_057857.2	Nef	downregulates	11289809, 12526811, 18438604, 19149577, 20702582	HIV-1 Nef downregulates expression of MHC-I by blocking transport of MHC-I molecules to the cell surface through a mechanism that requires phosphoinositide 3-kinase (PI 3-kinase) activity
HLA-A	156110	NP_057857.2	Nef	downregulates	11438519,	HIV-1 Nef downregulates MHC-I in Jurkat cells in a concentration-dependent manner

HLA-A	156110	NP_057857.2	Nef	downregulates	11500821,	A dominant-negative mutant protein derived from Hck, (composed of the N-terminal region, SH2, and SH3 domains) interacts with HIV-1 Nef and inhibits Nef-induced downregulation of MHC class I
HLA-A	156110	NP_057857.2	Nef	downregulates	11578695, 16454711	Deletion of the 19 N-terminal amino acids including the myristoylation signal from HIV-1 Nef inhibits both MHC-I and CD4 downregulation while preserving most CTL, T-helper and B-cell epitopes
HLA-A	156110	NP_057857.2	Nef	downregulates	11602047,	Downregulation of major histocompatibility class I on human dendritic cells by HIV-1 Nef impairs antigen presentation to HIV-specific CD8+ T lymphocytes
HLA-A	156110	NP_057857.2	Nef	downregulates	12097566, 15611225	HIV-1 Nef-mediated downregulation of HLA class I suppresses the cytolytic activity of HIV-1-specific cytotoxic T-lymphocyte (CTL) clones
HLA-A	156110	NP_057857.2	Nef	downregulates	12482663, 12884192	HIV-1 Nef downregulates human MHC-I more efficiently than murine MHC-I molecules in HeLa cells, and Nef does not function efficiently in murine endothelial cells
HLA-A	156110	NP_057857.2	Nef	downregulates	14557639, 16354571, 16454711, 16684552	HIV-1 Nef alleles derived from perinatally infected children efficiently downregulate both CD4 and MHC-I in HeLa-CD4+ cells
HLA-A	156110	NP_057857.2	Nef	downregulates	15194762,	HIV-1 group N and group O Nef alleles only weakly downregulate CD4, CD28, and class I and II MHC molecules
HLA-A	156110	NP_057857.2	Nef	downregulates	15262497,	HIV-1 Nef has been observed to downregulate HLA-A2 on immature dendritic cells from two donors
HLA-A	156110	NP_057857.2	Nef	downregulates	15878340,	The HIV-1 Nef mutant NefAAAA, which cannot interact with the endosomal sorting protein PACS-1, increases the number of cells containing long and stable tubules, which allows the internalization of MHC-1 into the tubules from the cell surface
HLA-A	156110	NP_057857.2	Nef	downregulates	16000390, 16091223	Macrophage-tropic HIV-1 Nef downregulates expression of HLA-A2 on the surface of productively infected macrophages; point mutations in Nef at prolines P74 or P80 abrogate the downregulation of HLA-A2
HLA-A	156110	NP_057857.2	Nef	downregulates	16365153,	HIV-1 Nef induces drastic and moderate downregulation of CD4 and MHC-I in resting CD4(+) T lymphocytes, respectively, but markedly upregulates cell surface levels of the MHC-II invariant chain CD74
HLA-A	156110	NP_057857.2	Nef	downregulates	16847125,	Mutation of amino acid P78 in HIV-1 Nef affects downregulation of MHC-I molecules from the cell surface, but does not interfere with Nef binding to Src homology 3 (SH3) domains
HLA-A	156110	NP_057857.2	Nef	downregulates	17581864, 20622010, 25585010	Knocking down either AP-1 gamma, AP-1 mu1, or clathrin strongly inhibits Nef-induced downregulation of HLA-A2
HLA-A	156110	NP_057857.2	Nef	downregulates	18005690, 18296443, 25585010	Nef/Hck complex recruits and phosphorylates the tyrosine kinase ZAP-70, which binds class I PI3K to trigger MHC-I downregulation in primary CD4+ T cells
HLA-A	156110	NP_057857.2	Nef	downregulates	18005690, 25585010	In promonocytic cells, Nef/Hck recruits the ZAP-70 homolog Syk to downregulate MHC-I
HLA-A	156110	NP_057857.2	Nef	downregulates	18073204,	Mutating three amino acids (Y320, A324, and D327) in the cytoplasmic tail of HLA-A2 abrogates Nef-induced downregulation of HLA-A2 through a failuer to recruit the mu1 or gamma subunits of AP-1
HLA-A	156110	NP_057857.2	Nef	downregulates	18296443, 25585010	HIV-1 Nef-mediated downregulation of MHC-I requires Nef motif EEEE(65)-dependent binding to the sorting protein PACS-2, which targets Nef to the paranuclear region and enables Nef PXXP(75) to bind and activate a trans-Golgi network localized Src kinase
HLA-A	156110	NP_057857.2	Nef	downregulates	20622010, 21917951	ARF6(T27N/Q67L) and RAB11(Q67L) mutants induce significant reversal of HLA-I A2 downregulation by HIV-1 Nef through redistributing HLA-I A2 from the perinuclear vesicles to the peripheral punctate vesicles at the plasma membrane
HLA-A	156110	NP_057857.2	Nef	downregulates	21543478, 23170180, 23202450, 25585010	beta-COP as a cellular cofactor is required for HIV-1 Nef-mediated HLA-A2, CD4, and CD8 downregulation
HLA-A	156110	NP_057857.2	Nef	downregulates	22301137,	Double (W13A/V16R) and triple (W13A/V16R/M20A) substitution mutants of HIV-1 Nef fail to downregulate MHC-I
HLA-A	156110	NP_057857.2	Nef	downregulates	22537596,	The HIV-1 Nef highly conserved valine-glycine-phenylalanine amino acid triplet (VGF) motif, which links the acidic cluster and the proline-rich motif, is important for downregulation of CXCR4 and MHC-I
HLA-A	156110	NP_057857.2	Nef	downregulates	22553319,	HIV-1 Nef with A84D, Y135F, and G140R mutation impairs to its ability to downregulate MHC-I
HLA-A	156110	NP_057857.2	Nef	downregulates	22826228,	HLA-A2 molecules with HLA-A cytoplasmic domains are more downregulated by HIV-1 Nef than those with HLA-B domains. There is no downregulation of HLA-A2 with HLA-C cytoplasmic domains by Nef
HLA-A	156110	NP_057857.2	Nef	downregulates	23289738,	HIV-1 Nef clones, isolated from plasma of elite controllers (EC) and chronic progressors (CP), show significantly lower HLA class I downregulation activity in EC than that in CP
HLA-A	156110	NP_057857.2	Nef	downregulates	24041011, 25193656	HIV-1 Nef clones obtained from chronic patients infected with HIV-1 subtypes A, B, C or D show a functional hierarchy of subtype B > A/D > C for Nef-mediated HLA class I downregulation
HLA-A	156110	NP_057857.2	Nef	downregulates	24965469,	HIV-1 Nef clones from acute controllers display a lesser ability to downregulate CD4 and HLA class I from the cell surface, and a reduced ability to enhance virion infectivity compared to those from acute progressors
HLA-A	156110	NP_057857.2	Nef	downregulates	26439863,	HIV-1 Nef downregulates cell (CEMT4) surface expression of HLA-A
HLA-A	156110	NP_057857.2	Nef	downregulates	26607225,	HIV-1 NL4-3 and SK68 Nef downregulates HLA-A (HLA-A*02), which is dependent upon amino acids M20 and S88
HLA-A	156110	NP_057857.2	Nef	downregulates	26656785,	HIV-1 (SF2) Nef downregulates MHC-I (HLA-A/B/C); downregulation is dependent upon a proline-rich SH3 binding domain in Nef
HLA-A	156110	NP_057857.2	Nef	downregulates	26700863,	HIV-1 NL4-3 Nef downregulates HLA-A/B/C, which moderately requires the CPG-motif in Nef
HLA-A	156110	NP_057857.2	Nef	downregulates	26787826,	HIV-1 NL4-3 and subtype B Nef downregulates HLA-A more than HLA-B, which discerned by amino acid 202 in Nef

HLA-A	156110	NP_057857.2	Nef	downregulates	8612235, 12734410, 15638726, 16091223, 16272310, 16979207, 16987968, 17077296, 17581864, 17586321, 17632197, 17632570, 18005680, 18005690, 18073204, 18155264, 18296443, 18438604, 18473783, 18541215, 18653452, 18725938, 18808677, 19091857, 19149577, 19449444, 19555986, 19643141, 19770068, 20012528, 20380698, 20594957, 20702582, 21068258, 21165790, 21209113, 21482738, 21543478, 21762823, 21849975, 21861776, 21917951, 21922073, 21994772, 22103831, 22103833, 22103834, 22175768, 22301137, 22301152, 22537596, 22553319, 22613796, 22651890, 22826228, 22844345, 22980333, 23170180, 23202450, 23289738, 23490051, 23847689, 23853598, 23986795, 24023945, 24041011, 24058696, 24158818, 24172637, 24192765, 24400003, 24495362, 24748005, 24789790, 24904546, 24965469, 25193656, 25275127, 25423108, 25525794, 25827531,	HIV-1 Nef downregulates the expression of MHC-I at the surface of lymphoid, monocytic and epithelial cells, causing MHC-I molecules to be rapidly internalized, accumulated in endosomal vesicles and degraded
HLA-A	156110	NP_057857.2	Nef	downregulates	9052838, 9586638, 18438604, 18808677, 19149577, 19847956, 20622010, 22705789, 22767237, 23170180, 23202450	Interaction of HIV-1 Nef with the mu subunit of AP adaptor complexes requires the recognition of tyrosine-based sorting signals, which likely facilitates the connection between MHC I and the clathrin-dependent sorting machinery during MHC I downregulation
HLA-A	156110	NP_057857.2	Nef	downregulates	9123874,	Downregulation of MHC-I by HIV-1 Nef decreases the incorporation of MHC-I molecules into virions, but does not decrease virion infectivity
HLA-A	156110	NP_057857.2	Nef	inhibits	11289809, 12584329, 20622010, 23170180, 23202450, 23678182, 25915798	Expression of HIV-1 Nef in human T cells inhibits HLA-A2 transport to the cell surface
HLA-A	156110	NP_057857.2	Nef	inhibits	15569716, 20622010, 23678182	The ability of HIV-1 Nef to disrupt MHC-I trafficking and inhibit antigen presentation is regulated by the expression of the mu1 subunit of adaptor protein (AP) AP-1A, a cellular protein complex implicated in TGN to endolysosomal pathways
HLA-A	156110	NP_057857.2	Nef	inhibits	15596859, 23170180, 23202450	The N-terminal alpha helix (17-26), polyproline (72-78), acidic (62-65), and oligomerization (123) domains of HIV-1 Nef are required for Nef-mediated disruption of the transport of HLA-A2 to the cell surface and for Nef to coprecipitate with HLA-A2
HLA-A	156110	NP_057857.2	Nef	interacts with	17581864,	Knocking down AP-2 enhances Nef activity by causing increased delivery of HLA-A2 to a prelysosomal compartment
HLA-A	156110	NP_057857.2	Nef	interacts with	17878955, 25165114	Protective HLA alleles have a true preference for HIV-1 Gag protein, while non-protective HLA alleles preferentially interact with HIV-1 Nef
HLA-A	156110	NP_057857.2	Nef	interacts with	18057255, 18073204	HIV-1 Nef acidic (Glu62-65) and polyproline domains (Pro75/78) stabilize the interaction between the HLA-A2/Nef fusion protein and AP-1 mu1
HLA-A	156110	NP_057857.2	Nef	interacts with	23061377, 24886641	HLA supertypes such as HLA B*07, HLA B*58, HLA A*02 and HLA A*03 are most successful in restricting the amino acid positions of epitope dense regions of HIV-1 Nef, CA, and MA with low entropy and hydrophobic property
HLA-A	156110	NP_057857.2	Nef	interacts with	26061722,	HIV-1 Nef interacts with HLA-A (MHC1) and this interaction occurs partially within RAB5+ early endosomes
HLA-A	156110	NP_057857.2	Nef	modulates	11396948, 14557639, 22613796, 23490051	Different levels of MHC-I modulation are induced by different HIV-1 Nef proteins derived from HIV-1 infected adults and children
HLA-A	156110	NP_057857.2	Nef	modulates	9971776, 12502873, 15078178, 17170457, 18005690, 18073204	Two distinct regions of HIV-1 Nef modulate MHC-I cell surface expression: an N-terminal alpha-helix (residues 17-26) and a proline-rich motif (residues 75-78)
HLA-A	156110	NP_057857.2	Nef	relocalizes	20622010,	HIV-1 Nef sequesters HLA-I A2 and colocalizes with CD63 and LAMP1 markers in late endosomes and lysosomes
HLA-A	155871	NP_057853.1	Tat	downregulates	17604883,	Four mutations (C27S, K51T, R55L, and G79A) on HIV-1 Tat result in the loss of the deleterious effects of Tat on the expression of MHC I, IL-2, and CD25 genes compared with wild-type Tat in Jurkat cells
HLA-A	155871	NP_057853.1	Tat	downregulates	7621073, 8493575, 9751712, 9840288	HIV-1 Tat represses the MHC class I gene promoter by binding to and repressing TAFII250, a component of the general transcription factor TFIID, suggesting a mechanism for HIV-1 to downregulate MHC class I expression and avoid immune surveillance
HLA-A	155871	NP_057853.1	Tat	upregulates	11751963, 14595379	HIV-1 Tat upregulates MHC class I in monocyte-derived dendritic cells and CD8(+) T cells, thereby driving T cell-mediated immune responses
HLA-A	155807	NP_057852.2	Vpr	upregulates	23874603,	A stable-isotope labeling by amino acids in cell culture coupled with mass spectrometry-based proteomics identifies upregulation of HLA-A (A-68 alpha chain) expression by HIV-1 Vpr in Vpr transduced macrophages
HLA-A	155945	NP_057855.1	Vpu	downregulates	9104816, 18672082, 20012522, 24400003, 25275127, 25620704	Using antibodies specific to MHC class I A, B, and C molecules (clone W6/32), HIV-1 Vpu protein has been shown to downregulate the expression of MHC class I molecules on the surface of HIV-1 infected cells
HLA-A	155945	NP_057855.1	Vpu	regulated by	22503975,	HLA class I-associated immune responses have minor effects on Vpu variability, suggesting that Vpu conformation and function are preserved through many possible combinations of primary and secondary polymorphisms
HLA-A			HIV-1 virus replication	downregulates expression of human gene	26439863,	HIV-1 infection (VSV-G pseudotyped) of CEMT4 T cells downregulates plasma membrane expression of HLA-A
HLA-A			HIV-1 virus replication	downregulates expression of human gene	26497177,	Capsid expressing (p24+) cells from pleural fluid of HIV-1/TB coinfected patients or in vitro infected PBMC (NL4-3 or NLAD8) downregulate HLA-A/B/C and BST2 (Tetherin) concomitantly with CD4 downregulation
GSN	155971	NP_579894.2	Envelope surface glycoprotein gp120	inhibited by	23575248,	Gelsolin overexpression impairs HIV-1 gp120-induced cortical F-actin reorganization and capping and gp120-mediated CD4-CCR5 and CD4-CXCR4 redistribution in permissive lymphocytes

GSN	155871	NP_057853.1	Tat	downregulates	16526095,	In Jurkat cells expressing HIV-1 Tat, decreased expression levels are found for basic cytoskeletal proteins such as actin, beta-tubulin, annexin, cofilin, gelsolin, and Rac/Rho-GDI complex
GSN	155807	NP_057852.2	Vpr	inhibited by	17254575,	The G5 domain of gelsolin inhibits HIV-Vpr-induced T-cell apoptosis by blocking the interaction between Vpr and VDAC
GSN	155807	NP_057852.2	Vpr	upregulates	23874603,	A stable-isotope labeling by amino acids in cell culture coupled with mass spectrometry-based proteomics identifies upregulation of gelsolin (GSN) expression by HIV-1 Vpr in Vpr transduced macrophages
GSN			HIV-1 virus replication	enhanced by expression of human gene	23575248,	Knockdown of gelsolin by siRNA inhibits early HIV-1 infection and HIV-1 Env (gp120/gp41)-mediated membrane fusion in permissive lymphocytes
P4HB	155971	NP_579894.2	Envelope surface glycoprotein gp120	cleaved by	12218051, 12218052, 23206338	Protein-disulfide isomerase (PDI) cleaves disulfide bonds in recombinant HIV-1 envelope glycoprotein gp120, and gp120 bound to the surface receptor CD4 undergoes a disulfide reduction that is prevented by PDI inhibitors
P4HB	155971	NP_579894.2	Envelope surface glycoprotein gp120	interacts with	20458450,	The disulfide cross-linking interaction between gp120 and PDI is enhanced by CD4 protein
P4HB	155971	NP_579894.2	Envelope surface glycoprotein gp120	interacts with	23206338,	PDI is predominantly involved in HIV-1 entry and infection of the T cell line PM-1 and PHA-stimulated primary T lymphocytes, suggesting the preferential use of PDI relevant to the HIV-1 entry and establishment of virus reservoirs in resting CD4+ cells
P4HB	155971	NP_057856.1	Envelope surface glycoprotein gp160, precursor	interacts with	22190034,	HIV-1 gp160 is identified to have a physical interaction with prolyl 4-hydroxylase, beta polypeptide (P4HB) in human HEK293 and/or Jurkat cell lines by using affinity tagging and purification mass spectrometry analyses
P4HB	155971	NP_057856.1	Envelope surface glycoprotein gp160, precursor	processed by	17301129,	Treatment of trimeric HIV-1 rgp140 with protein disulfide isomerase yields monomers by disruption of the intermolecular disulfide bonds
P4HB	155908	NP_057854.1	Rev	interacts with	22174317,	HIV-1 Rev interacting protein, prolyl 4-hydroxylase, beta polypeptide (P4HB), is identified by the in-vitro binding experiments involving cytosolic or nuclear extracts from HeLa cells. The interaction of Rev with P4HB is increased by RRE
P4HB	155807	NP_057852.2	Vpr	upregulates	23874603,	A stable-isotope labeling by amino acids in cell culture coupled with mass spectrometry-based proteomics identifies upregulation of prolyl 4-hydroxylase, beta polypeptide (P4HB, PDIA1) expression by HIV-1 Vpr in Vpr transduced macrophages
PFN1	155807	NP_057852.2	Vpr	downregulates	23874603,	A stable-isotope labeling by amino acids in cell culture coupled with mass spectrometry-based proteomics identifies downregulation of profilin 1 (PFN1) expression by HIV-1 Vpr in Vpr transduced macrophages
THBS1	155971	NP_579894.2	Envelope surface glycoprotein gp120	binds	9419208,	Interaction of TSP1 with HIV-1 gp120 involves CSVTCG sequences in the type 1 properdin-like repeats of TSP1 and amino acids 281-300, 311-330, and 361-380 in the C2-V3-C3 domains of gp120
THBS1	155971	NP_579894.2	Envelope surface glycoprotein gp120	binds	9419208,	TSP1 inhibits HIV-1 infection of peripheral blood mononuclear cells and transformed T and promonocytic cell lines by its binding to HIV-1 gp120
THBS1	155871	NP_057853.1	Tat	binds	11023976,	Thrombospondin-1 (TSP) binds to HIV-1 Tat, an interaction that can be inhibited by heparin which can bind to both TSP and Tat
THBS1	155871	NP_057853.1	Tat	inhibited by	10398144,	Thrombospondin-1 (TSP) prevents endothelial cell motility induced by HIV-1 Tat and inhibits angiogenic activity exerted by Tat in the Matrigel sponge model, suggesting downregulation of TSP may be permissive for development of KS-associated angiogenesis
THBS1	155871	NP_057853.1	Tat	inhibited by	11023976,	Thrombospondin-1 inhibits cell internalization and HIV-1 LTR transactivating activity of extracellular HIV-1 Tat, cell interaction and mitogenic activity of extracellular Tat, as well as the autocrine loop of stimulation exerted by endogenous Tat
THBS1	155807	NP_057852.2	Vpr	stimulates	12444143,	HIV-1 Vpr potentiates the stimulation of thrombospondin 1 by glucocorticoids via the glucocorticoid receptor pathway
CD14	156110	NP_057857.2	Nef	downregulates	22808111,	CD14 is significantly downregulated from the surface of HIV-1 Nef-expressing THP-1 monocytes compared to that from the surface of control cells
CD14	156110	NP_057857.2	Nef	upregulates	12414752,	HIV-1 Nef enhances membrane-bound (m) CD14 expression on monocytes but does not induce the release of soluble CD14 into the culture supernatants of PBMC; the upregulation of mCD14 expression does not involve endogenously produced IL-10
FCGR3A	155971	NP_579894.2	Envelope surface glycoprotein gp120	binds	21832933,	IgG2 and IgG4 bind more poorly to enzymatically deglycosylated recombinant gp120 (rgp120) than to unchanged rgp120
FCGR3A	155971	NP_579894.2	Envelope surface glycoprotein gp120	downregulates	22496218,	NK cells that respond with IFN-gamma and TNF-alpha cytokine production to HIV-1 gp120 peptides have reduced CD16 and NKp46 expression and have increased levels of CD57
FCGR3A	156110	NP_057857.2	Nef	antagonizes	25980612,	Production of infectious triple deletion vpr/vpu/nef HIV-1 mutant is suppressed, indicating that Nef may antagonize restriction activity of FCGR3A against production of infectious wild-type HIV-1
FCGR3A	155871	NP_057853.1	Tat	inhibits	9743356,	HIV-1 Tat inhibits the rise in intracellular free calcium concentration in Natural Killer (NK) cells upon cross-linking of the adhesion molecule CD11a and the activation molecule CD16, indicating Tat is involved in the impairment of NK cell function
FCGR3A	155871	NP_057853.1	Tat	upregulates	25250834,	HIV-1 Tat upregulates the expression of CCR2, CD16, and TLR4 in monocyte-derived macrophages
FCGR3A	155807	NP_057852.2	Vpr	antagonizes	25980612,	Production of infectious triple deletion vpr/vpu/nef HIV-1 mutant is suppressed, indicating that Vpu may antagonize restriction activity of FCGR3A against production of infectious wild-type HIV-1
FCGR3A	155945	NP_057855.1	Vpu	antagonizes	25980612,	Production of infectious triple deletion vpr/vpu/nef HIV-1 mutant is suppressed, indicating that Vpu may antagonize restriction activity of FCGR3A against production of infectious wild-type HIV-1
FCGR3A	155945	NP_057855.1	Vpu	interacts with	24623433, 25396265	The ability of HIV-1 Vpu to antagonize tetherin is important for the antibody opsonization of HIV-infected cells, which in turn increases FCGRIII (CD16) signaling
FCGR3A			HIV-1 virus replication	affected by expression of human gene	26613093,	HIV-1 is vertically transmitted more readily by mothers who are heterozygous for high-affinity and low-affinity FCGR3A
C4B	155971	NP_579894.2	Envelope surface glycoprotein gp120	binds	7590866,	Amino acid residues 410-449 of HIV-1 gp120 are involved in its binding to C4b
C4B	155971	NP_579894.2	Envelope surface glycoprotein gp120	binds	7642209, 7893437, 7911492	Complement proteins C4, C3d, C5b-9, and properdin bind to HIV-1 gp120-coated CD4+ T cells of healthy individuals when incubated in autologous serum
C4B	155971	NP_579894.2	Envelope surface glycoprotein gp120	binds	8630395,	A synthetic peptide covering positions 233-251 of the HIV-1 gp120 protein binds to complement proteins C3, C4, C5, C9, and properdin
C4B	155971	NP_579894.2	Envelope surface glycoprotein gp120	interacts with	7893437,	Preincubation of HIV-1 gp41 with either factor H or properdin, and of HIV-1 gp120 with C3b or C4b affect the interaction between HIV-1 gp41 and gp120
HSPA5	155971	NP_579894.2	Envelope surface glycoprotein gp120	complexes with	23125841,	Tandem affinity purification and mass spectrometry analysis identify heat shock 70kDa protein 5 (HSPA5; 78kDa glucose-regulated protein), HIV-1 Gag, Gag/Pol, gp120, and Nef incorporated into staufen1 RNP complexes isolated from HIV-1-expressing cells
HSPA5	155971	NP_579894.2	Envelope surface glycoprotein gp120	inhibited by	12832005,	Over expression of hsp70 with a herpes viral amplicon vector protects cultured hippocampal rat neurons from gp120 neurotoxicity
HSPA5	155971	NP_579894.2	Envelope surface glycoprotein gp120	upregulates	26740125,	HIV-1 Env gp120 upregulates HSPA5 (GRP78/BiP) in SVGA cells and human fetal astrocytes
HSPA5	155971	NP_579894.2	Envelope surface glycoprotein gp120	upregulates	7906708,	The exposure of permissive CD4+ cells to HIV-1 gp120 increases the synthesis and nuclear translocation of 70kDa heat shock protein
HSPA5	155971	NP_057856.1	Envelope surface glycoprotein gp160, precursor	interacts with	1900540, 10514465	Newly synthesized HIV-1 gp160 interacts with GRP78-BiP in pulse-chase experiments; the interaction sites of gp160 with BiP include residues 115-132, 484-490, 602-616, 676-690, and 776-807
HSPA5	155971	NP_057856.1	Envelope surface glycoprotein gp160, precursor	interacts with	27375898,	HIV-1 gp160 interacts with HSPA5; predicted interaction to be within the endoplasmic reticulum and function as chaperone for endoplasmic reticulum-associated degradation
HSPA5	155030	NP_057850.1	Pr55(Gag)	complexes with	23125841,	Tandem affinity purification and mass spectrometry analysis identify heat shock 70kDa protein 5 (HSPA5; 78kDa glucose-regulated protein), HIV-1 Gag, Gag/Pol, gp120, and Nef incorporated into staufen1 RNP complexes isolated from HIV-1-expressing cells
HSPA5	155030	NP_057850.1	Pr55(Gag)	incorporates	11932435,	Hsp70 co-sediments with HIV-1 capsid protein in sucrose density gradients, providing evidence that it is specifically incorporated into HIV-1 virions through an interaction with HIV-1 Gag proteins
HSPA5	155030	NP_057850.1	Pr55(Gag)	incorporates	11932435, 21738476	Hsp70 is incorporated into HIV-1 virions through an interaction with HIV-1 Gag

HSPA5	155030	NP_579876.2	matrix	stimulated by	10964507,	Hsp70 facilitates nuclear import of HIV-1 preintegration complexes by stimulating the binding of HIV-1 Matrix to karyopherin alpha
HSPA5	155348	NP_057849.4	Gag-Pol	complexes with	23125841,	Tandem affinity purification and mass spectrometry analysis identify heat shock 70kDa protein 5 (HSPA5; 78kDa glucose-regulated protein), HIV-1 Gag, Gag/Pol, gp120, and Nef incorporated into staufen1 RNP complexes isolated from HIV-1-expressing cells
HSPA5	156110	NP_057857.2	Nef	complexes with	23125841,	Tandem affinity purification and mass spectrometry analysis identify heat shock 70kDa protein 5 (HSPA5; 78kDa glucose-regulated protein), HIV-1 Gag, Gag/Pol, gp120, and Nef incorporated into staufen1 RNP complexes isolated from HIV-1-expressing cells
HSPA5	156110	NP_057857.2	Nef	interacts with	21763498,	Heat shock proteins Hsp40 and Hsp70 interact with HIV-1 Nef and form a complex in cells
HSPA5	155871	NP_057853.1	Tat	activates	20457808,	Exposure of human umbilical vein endothelial cells to HIV-1 Tat causes broad activation of the unfolded-protein response in ER with phosphorylation of PERK, eIF2alpha, and JNK and induction of Grp78/BiP
HSPA5	155871	NP_057853.1	Tat	regulated by	10617616,	Hsp70 and Hsp90 and Cdc37 regulate the stabilization and folding of CDK9 as well as the assembly of an active CDK9/cyclin T1 complex responsible for P-TEFb-mediated HIV-1 Tat transactivation
HSPA5	155807	NP_057852.2	Vpr	competes with	10964507, 19275587	HIV-1 Vpr competes with Hsp70 for binding to karyopherin alpha
HSPA5	155807	NP_057852.2	Vpr	interacts with	21763498,	HIV-1 Vpr is required for the inhibitory effect of Hsp70 on viral gene expression and replication
HSPA5	155807	NP_057852.2	Vpr	upregulates	22438978,	HIV-1 Vpr significantly increases expression level of GRP78 in the endoplasmic reticulum
HSPA5	155807	NP_057852.2	Vpr	upregulates	23874603,	A stable-isotope labeling by amino acids in cell culture coupled with mass spectrometry-based proteomics identifies upregulation of heat shock 70kDa protein 5 (HSPA5, GRP78) expression by HIV-1 Vpr in Vpr transduced macrophages
ACTN1	155348	NP_705926.1	retropepsin	cleaves	1991513, 8997639	Alpha-actinin is cleaved in vitro by HIV-1 protease at amino acid residues 166-167 and 471-472
ACTN1	155807	NP_057852.2	Vpr	upregulates	27114546,	HIV-1 Vpr upregulates ACTN1 in HeLa cells within 12 hours of exposure
RNH1	155971	NP_057856.1	Envelope surface glycoprotein gp160, precursor	interacts with	27375898,	HIV-1 gp160 interacts with RNH1
RNH1	155030	NP_579881.1	nucleocapsid	interacts with	22190034,	HIV-1 NC is identified to have a physical interaction with ribonuclease/angiogenin inhibitor 1 (RNH1) in human HEK293 and/or Jurkat cell lines by using affinity tagging and purification mass spectrometry analyses
RNH1	155348	NP_705926.1	retropepsin	cleaves	22944692,	Positional proteomics analysis identifies the cleavage of human ribonuclease/angiogenin inhibitor 1 (RNH1) at amino acid residues 445-446 by the HIV-1 protease
RNH1			HIV-1 virus replication	enhanced by expression of human gene	18854154,	Knockdown of ribonuclease/angiogenin inhibitor 1 (RNH1) by siRNA inhibits the early stages of HIV-1 replication in 293T cells infected with VSV-G pseudotyped HIV-1
SELL	155971	NP_579894.2	Envelope surface glycoprotein gp120	downregulates	10449768, 14576059, 22842622	CD4 ligation by HIV-1 gp120 induces metalloproteinase-dependent L-selectin downregulation in primary resting CD4+ T cells
SELL	155971	NP_579894.2	Envelope surface glycoprotein gp120	downregulates	14576059,	L-selectin downregulation induced by HIV-1 gp120 is completely reversed by AMD3100 (a CXCR4 antagonist), but not SDF-1 alpha
SELL	156110	NP_057857.2	Nef	downregulates	25275127, 25822027	Both HIV-1 Nef and Vpu downregulate the cell surface expression of selectin L (CD62L)
SELL	156110	NP_057857.2	Nef	downregulates	25822027,	HIV-1 Nef mutants L37Q, 62EEEE/AAAA65, P72A/P75A, P78L, and F191A partially impair to downregulate CD62L in Jurkat cells
SELL	155871	NP_057853.1	Tat	upregulates	24667918,	Microarray analysis indicates HIV-1 Tat-induced upregulation of selectin L (SELL) in primary human brain microvascular endothelial cells
SELL	155945	NP_057855.1	Vpu	downregulates	25275127, 25822027	Both HIV-1 Nef and Vpu downregulate the cell surface expression of selectin L (CD62L)
SELL	155945	NP_057855.1	Vpu	inhibits	25822027,	HIV-1 Vpu inhibits the transport of newly synthesized CD62L molecules toward the cell surface of Jurkat cells
HSP90B1	155971	NP_057856.1	Envelope surface glycoprotein gp160, precursor	interacts with	27375898,	HIV-1 gp160 interacts with HSP90B1; predicted interaction to be within the endoplasmic reticulum and function as chaperone for endoplasmic reticulum-associated degradation
HSP90B1	156110	NP_057857.2	Nef	cooperates with	24204260,	Microvesicles, which contaminate purified HIV-1 inocula due to similar size and density, contain viral protein Nef and cellular proteins HSP90alpha and HSP90beta that are capable of potent stimulation of dendritic cells maturation and ICAM-1 expression
HSP90B1	156110	NP_057857.2	Nef	requires	25496667,	Genome-wide shRNA screening identifies HSP90B1, which is required for HIV-1 Nef-induced downregulation of CD4 in HeLa CD4+ cells
HSP90B1	155908	NP_057854.1	Rev	interacts with	22174317,	HIV-1 Rev interacting protein, heat shock protein 90kDa beta (HSP90B1), is identified by the in-vitro binding experiments involving cytosolic or nuclear extracts from HeLa cells. The interaction of Rev with HSP90B1 is increased by RRE
NME1	155348	NP_705927.1	reverse transcriptase	complexes with	25766862,	HIV-1 RT forms a complex with NDPKA in vitro
NME1			HIV-1 virus replication	enhanced by expression of human gene	19266025,	SiRNA-mediated knockdown of one of the components of the SET complex, NM23-H1, inhibits HIV-1 infection with significantly reduced levels of integrated HIV-1 DNA and viral production in HeLa-CD4 cells
PECAM1	155871	NP_057853.1	Tat	upregulates	23301033,	HIV-1 Tat enhances vIL-6-induced angiogenesis and tumorigenesis of fibroblasts and human endothelial cells, which correlates with upregulation of CD31, CD34, SMA, VEGF, b-FGF, and cyclin D1 expression
PECAM1	155807	NP_057852.2	Vpr	upregulates	23874603,	A stable-isotope labeling by amino acids in cell culture coupled with mass spectrometry-based proteomics identifies upregulation of platelet/endothelial cell adhesion molecule 1 (PECAM1, PECA1) expression by HIV-1 Vpr in Vpr transduced macrophages
VCL	155030	NP_579876.2	matrix	interacts with	22017400,	HIV-1 MA co-localizes with beta2 integrin, alphaM and alphaX integrins in the intracellular thick electron-dense membrane compartments, which contain talin, vinculin and paxillin that connect the integrin complexes to the actin cytoskeleton
VCL	156110	NP_057857.2	Nef	co-localizes with	25527710, 25745180	HIV-1 Nef co-localizes with vinvulin inside podosomes in human monocyte-derived macrophages
VCL	156110	NP_057857.2	Nef	polarizes	14597672,	HIV-1 Nef induces polarization of vinculin, a molecule important for dendritic cell (DC) adhesion, motility, and maturation, at one pole of DCs corresponding to the substrate-adhering portion or leading edge of the cell
CR2	155971	NP_579894.2	Envelope surface glycoprotein gp120	binds	8474169, 21161615	HIV-1 particles (gp120/gp41) bind to MT-2 (CD4+ CR2+) and Raji-3 (CD4- CR2+) cells but not to CEM (CD4+ CR2-) cells, suggesting that the virus binds to CR2 independently of CD4
CR2	155971	NP_057856.1	Envelope surface glycoprotein gp160, precursor	interacts with	15603708, 21161615	The activation of mitogen-activated protein kinases (MAPKs, including ERK, JNK, and p38MAPK) is induced by incubation of HIV-1 gp160 with CD4+complement receptor type 2 (CR2)+ cells
CR2	155030	NP_579880.1	capsid	localized by	26623655,	HIV-1 CA p24 is localized to recycling endosomes by CR2 (CD21) and soluble CD21 frees virus from follicular dendritic cells from lymph nodes of HIV infected patients
CR2	155459	NP_057851.1	Vif	downregulates	23333304,	HIV-1 Vif downregulates the expression of complement component receptor 2 (CR2) in Vif-expression T cells
CR2			HIV-1 virus replication	enhanced by expression of human gene	18976975,	Knockdown of complement component receptor 2 (CR2) by siRNA inhibits HIV-1 replication in HeLa P4/R5 cells
CR2			HIV-1 virus replication	inhibited by expression of human gene	26623655,	HIV-1 transmission to CD4+ T cells from follicular dendritic cells from lymph nodes of infected patients is inhibited by soluble CD21
CFL1	155971	NP_579894.2	Envelope surface glycoprotein gp120	activates	18775311, 18808680, 20842205, 24778234	HIV-1 gp120-CXCR4 signaling triggers cofilin activation and actin reorganization, which are important for a post entry process leading to viral nuclear localization
CFL1	155971	NP_579894.2	Envelope surface glycoprotein gp120	induces phosphorylation of	23294842,	The N-terminal leucine-rich repeat fragment of Slit2 inhibits HIV-1 gp120-induced phosphorylation of both LIMK1 and cofilin
CFL1	155971	NP_579894.2	Envelope surface glycoprotein gp120	induces phosphorylation of	24778234,	CCR5 expression inhibits HIV-1 gp120-induced LIMK1 activation and cofilin phosphorylation in CD4/CXCR4 expressing 293T cells
CFL1	155971	NP_579894.2	Envelope surface glycoprotein gp120	interacts with	17572668, 18808680	Filamin-A-dependent activation of the RhoA-ROCK-LIMK-cofilin pathway is a major event in HIV-1 gp120-induced receptor clustering
CFL1	155030	NP_057850.1	Pr55(Gag)	incorporates	8892894,	The cytoskeletal proteins ezrin, moesin, and cofilin are incorporated into HIV-1 particles, presumably through their interaction with actin which binds to the nucleocapsid domain of HIV-1 Gag
CFL1	156110	NP_057857.2	Nef	induces phosphorylation of	19683683, 20147394	HIV-1 Nef inactivates cofilin by inducing its hyperphosphorylation via association with PAK2 activity

CFL1	156110	NP_057857.2	Nef	induces phosphorylation of	21923909,	HIV-1 Nef-induced LIMK1 activation and CFL1 phosphorylation are required for Nef-mediated inhibition of retinoid receptor function
CFL1	156110	NP_057857.2	Nef	induces phosphorylation of	22537596,	The HIV-1 Nef highly conserved valine-glycine-phenylalanine amino acid triplet (VGF) motif is important for Nef-PAK2 association and cofillin hyper-phosphorylation
CFL1	155871	NP_057853.1	Tat	downregulates	16526095,	In Jurkat cells expressing HIV-1 Tat, decreased expression levels are found for basic cytoskeletal proteins such as actin, beta-tubulin, annexin, cofilin, gelsolin, and Rac/Rho-GDI complex
CALR	155971	NP_579894.2	Envelope surface glycoprotein gp120	interacts with	22190034,	HIV-1 gp120 is identified to have a physical interaction with calreticulin (CALR) in human HEK293 and/or Jurkat cell lines by using affinity tagging and purification mass spectrometry analyses
CALR	155971	NP_057856.1	Envelope surface glycoprotein gp160, precursor	binds	8550632,	The ubiquitous eukaryotic protein calreticulin binds to newly synthesized HIV-1 gp160, suggesting that calreticulin might act as a chaperone
CALR	155971	NP_057856.1	Envelope surface glycoprotein gp160, precursor	interacts with	22190034,	HIV-1 gp160 is identified to have a physical interaction with calreticulin (CALR) in human HEK293 and/or Jurkat cell lines by using affinity tagging and purification mass spectrometry analyses
CALR	155908	NP_057854.1	Rev	interacts with	22174317,	HIV-1 Rev interacting protein, calreticulin (CALR), is identified by the in-vitro binding experiments involving cytosolic or nuclear extracts from HeLa cells. The interaction of Rev with CALR is increased by RRE
CALR	155807	NP_057852.2	Vpr	upregulates	23874603,	A stable-isotope labeling by amino acids in cell culture coupled with mass spectrometry-based proteomics identifies upregulation of calreticulin (CALR) expression by HIV-1 Vpr in Vpr transduced macrophages
GRN	155871	NP_057853.1	Tat	binds	10079180,	The cysteine rich region of HIV-1 Tat (amino acids 21-37) mediates the binding of Tat to granulin amino acids 206-337 (granulin regions B+A) suggesting a role for granulin growth factors as biologically important extracellular Tat co-factors
GRN	155871	NP_057853.1	Tat	inhibited by	12588988, 15653695, 20054825	Granulin forms stable complexes with cyclin T1 and HIV-1 Tat and inhibits Tat transactivation of the viral LTR promoter
TKT	155348	NP_705926.1	retropepsin	cleaves	22944692,	Positional proteomics analysis identifies the cleavage of human transketolase (TKT) at amino acid residues178-179 by the HIV-1 protease
TKT	155807	NP_057852.2	Vpr	downregulates	23874603,	A stable-isotope labeling by amino acids in cell culture coupled with mass spectrometry-based proteomics identifies downregulation of transketolase (TKT) expression by HIV-1 Vpr in Vpr transduced macrophages
PDIA3	155971	NP_579894.2	Envelope surface glycoprotein gp120	cleaved by	12218051, 12218052, 15644496, 16182193, 22230366	Protein-disulfide isomerase (PDI) cleaves disulfide bonds in recombinant HIV-1 envelope glycoprotein gp120, and gp120 bound to the surface receptor CD4 undergoes a disulfide reduction that is prevented by PDI inhibitors
PDIA3	155971	NP_579894.2	Envelope surface glycoprotein gp120	co-localizes with	24825317,	HIV-1 gp120/MBL complex co-localizes with the ER marker ERp57 and the Golgi marker p230 at subcellular perinuclear compartments in neuronal cells
PDIA3	155971	NP_579894.2	Envelope surface glycoprotein gp120	interacts with	20458450,	The disulfide cross-linking interaction between gp120 and PDI is enhanced by CD4 protein
PDIA3	155971	NP_579894.2	Envelope surface glycoprotein gp120	interacts with	23206338,	PDI is predominantly involved in HIV-1 entry and infection of the T cell line PM-1 and PHA-stimulated primary T lymphocytes, suggesting the preferential use of PDI relevant to the HIV-1 entry and establishment of virus reservoirs in resting CD4+ cells
PDIA3	155971	NP_057856.1	Envelope surface glycoprotein gp160, precursor	interacts with	22190034,	HIV-1 gp160 is identified to have a physical interaction with protein disulfide isomerase family A, member 3 (PDIA3) in human HEK293 and/or Jurkat cell lines by using affinity tagging and purification mass spectrometry analyses
PDIA3	155971	NP_057856.1	Envelope surface glycoprotein gp160, precursor	interacts with	27375898,	HIV-1 gp160 interacts with PDIA3; predicted interaction to be within the endoplasmic reticulum and function as a thioredoxin reductase
PDIA3	155971	NP_057856.1	Envelope surface glycoprotein gp160, precursor	processed by	17301129,	Treatment of trimeric HIV-1 rgp140 with protein disulfide isomerase yields monomers by disruption of the intermolecular disulfide bonds
PDIA3	155971	NP_579895.1	Envelope transmembrane glycoprotein gp41	interacts with	22190034,	HIV-1 gp41 is identified to have a physical interaction with protein disulfide isomerase family A, member 3 (PDIA3) in human HEK293 and/or Jurkat cell lines by using affinity tagging and purification mass spectrometry analyses
PDIA3	155348	NP_705926.1	retropepsin	cleaves	22944692,	Positional proteomics analysis identifies the cleavage of human protein disulfide isomerase family A, member 3 (PDIA3) at amino acid residues 27-28 by the HIV-1 protease
PDIA3	155807	NP_057852.2	Vpr	downregulates	23874603,	A stable-isotope labeling by amino acids in cell culture coupled with mass spectrometry-based proteomics identifies downregulation of protein disulfide isomerase A3 (PDIA3) expression by HIV-1 Vpr in Vpr transduced macrophages
PDIA3			HIV-1 virus replication	enhanced by expression of human gene	18976975,	Knockdown of protein disulfide isomerase family A, member 3 (PDIA3) by siRNA inhibits HIV-1 replication in HeLa P4/R5 cells
PPIF	155030	NP_579880.1	capsid	interacts with	25505242,	The interaction of HIV-1 CA with human cellular peptidylprolyl isomerase F protein (PPIF, cyclophilin F) is identified by yeast two-hybrid screen
MAN1A1	155971	NP_579894.2	Envelope surface glycoprotein gp120	processed by	12560567,	Specific alterations of the N-linked carbohydrates on HIV-1 gp120 and gp41 by glucosidases and mannosidase inhibitors can enhance mannose-binding lectin (MBL)-mediated neutralization of virus by strengthening the interaction of HIV-1 with MBL
MAN1A1	155971	NP_579894.2	Envelope surface glycoprotein gp120	processed by	2283726, 2355006, 2406237, 2542563, 2649653, 2829950, 8218172, 8892864	HIV-1 gp120 N-linked oligosaccharides are processed by manosidase I and II in the Golgi complex
MAN1A1	155971	NP_057856.1	Envelope surface glycoprotein gp160, precursor	processed by	2187500, 2541446, 2542563, 2649653, 2829950, 8673525, 9109416, 11530211, 18215327, 18314154, 18330979	Oligosaccharide side-chains of HIV-1 gp160 are processed by glycosidase I and II, mannosidase I and II, acetylglucosaminyl transferase I and II, and fucosyl, galactosyl and sialyl transferases in both the endoplasmic reticulum and golgi apparatus
MAN1A1	155971	NP_579895.1	Envelope transmembrane glycoprotein gp41	processed by	1736542, 2829950, 3099781, 3264072, 8093218	Mannose-containing, N-linked oligosaccharide side-chains of HIV-1 gp41 are involved in the initial stage of infection by HIV-1; glycosylation inhibitors block virus-cell and cell-cell fusion and release of the virions
MAN1A1			HIV-1 virus replication	enhanced by expression of human gene	18854154,	Knockdown of mannosidase, alpha, class 1A, member 1 (MAN1A1) by siRNA inhibits the early stages of HIV-1 replication in 293T cells infected with VSV-G pseudotyped HIV-1
RDX	155971	NP_579894.2	Envelope surface glycoprotein gp120	relocalizes	15818415,	Treatment of CD4+ T cells with HIV-1 gp120 induces CD95-mediated apoptosis, CD95/ERM protein (ezrin, radixin, moesin) colocalization and stable ezrin phosphorylation
RDX	155030	NP_057850.1	Pr55(Gag)	co-localizes with	24760896,	HIV-1 Gag co-localizes with ezrin-radixin-moesin proteins at polarized HIV-1 assembly sites in human T cells
RDX	155807	NP_057852.2	Vpr	downregulates	17349711,	HIV-1 Vpr-induced downregulation of sodium hydrogen exchanger, isoform 1 (NHE1), in Vpr(+) virus infected cells leads to acidification of cells, loss of ezrin, radixin and moesin (ERM) protein complex and decrease of AKT phosphorylation
MDH2	155348	NP_705926.1	retropepsin	cleaves	22944692,	Positional proteomics analysis identifies the cleavage of human malate dehydrogenase 2, NAD, mitochondrial (MDH2) at amino acid residues 94-95 by the HIV-1 protease
MDH2	155807	NP_057852.2	Vpr	downregulates	23874603,	A stable-isotope labeling by amino acids in cell culture coupled with mass spectrometry-based proteomics identifies downregulation of malate dehydrogenase 2 (MDH2) expression by HIV-1 Vpr in Vpr transduced macrophages
ARHGDIB	155807	NP_057852.2	Vpr	modulates	19655254,	The proteomic assay from Vpr-expressing HTLV-1 transformed cells reveals apoptosis related protein changes, such as CASP3 activity indicator proteins (vimentin and Rho GDP-dissociation inhibitor 2)
RAP1B	155030	NP_579880.1	capsid	downregulated by	24586238,	Prostaglandin E2-mediated HIV-1 inhibition requires the EPAC/RAP/RhoA signaling pathway by downregulation of HIV-1 CA production
RAP1B			HIV-1 virus replication	enhanced by expression of human gene	18187620,	Knockdown of RAP1B, member of RAS oncogene family (RAP1B) by siRNA inhibits HIV-1 replication in HeLa-derived TZM-bl cells
RAP1B	155030	NP_579880.1	capsid	downregulated by	24586238,	Prostaglandin E2-mediated HIV-1 inhibition requires the EPAC/RAP/RhoA signaling pathway by downregulation of HIV-1 CA production
RAP1B			HIV-1 virus replication	enhanced by expression of human gene	18187620,	Knockdown of RAP1B, member of RAS oncogene family (RAP1B) by siRNA inhibits HIV-1 replication in HeLa-derived TZM-bl cells

B2M	156110	NP_057857.2	Nef	downregulates	25275127,	Both HIV-1 Nef and Vpu downregulate the cell surface expression of beta-2-microglobulin (B2M)
B2M	155871	NP_057853.1	Tat	downregulates	9751712, 10199391, 21085635	HIV-1 Tat represses transcription of the beta 2-microglobulin (B2M) promoter, thereby downregulating B2M expression, suggesting a mechanism by which HIV-1 could prevent cell surface expression of the MHC class I complex and avoid immune detection
B2M	155945	NP_057855.1	Vpu	downregulates	25275127,	Both HIV-1 Nef and Vpu downregulate the cell surface expression of beta-2-microglobulin (B2M)
ACTA2	155971	NP_579894.2	Envelope surface glycoprotein gp120	induces reorganization of	18775311, 22640593	HIV-1 gp120-CXCR4 signaling triggers cofilin activation and actin reorganization, which are important for a post entry process leading to viral nuclear localization
ACTA2	155971	NP_579894.2	Envelope surface glycoprotein gp120	induces reorganization of	22535526,	Syntenin-1 is recruited toward HIV-1 gp120/gp41-driven virus/cell and cell/cell contacts, associates with CD4, limits HIV-1-induced cell fusion and viral entry, and modulates gp120/gp41-triggered actin polymerization and PIP2 accumulation
ACTA2	155971	NP_579894.2	Envelope surface glycoprotein gp120	induces reorganization of	23294842,	The N-terminal leucine-rich repeat fragment of Slit2 inhibits HIV-1 gp120-induced actin polymerization in T cells
ACTA2	155971	NP_579894.2	Envelope surface glycoprotein gp120	induces reorganization of	23575248,	Gelsolin overexpression impairs HIV-1 gp120-induced cortical F-actin reorganization and capping and gp120-mediated CD4-CCR5 and CD4-CXCR4 redistribution in permissive lymphocytes
ACTA2	155971	NP_579894.2	Envelope surface glycoprotein gp120	interacts with	18443296,	Inducible T-cell kinase (ITK) affects viral entry and gp120-induced actin reorganization
ACTA2	155971	NP_579894.2	Envelope surface glycoprotein gp120	upregulates	22479424,	HIV-1 X4-tropic gp120 upregulates alpha-SMA (ACTA2) and collagen I alpha 1 expression via the ERK1/2 pathway in a CXCR4-dependent manner in activated human hepatic stellate cells
ACTA2	155971	NP_057856.1	Envelope surface glycoprotein gp160, precursor	interacts with	17360745, 17504171	Treatment of cells with actin-depolymerizing agents or tubulin polymerization inhibitors largely reduces the percentage of cells with capped HIV-1 Gag and Env, indicating an intact actin and tubulin cytoskeleton is required for efficient assembly of HIV-1
ACTA2	155971	NP_579895.1	Envelope transmembrane glycoprotein gp41	induces reorganization of	22535526,	Syntenin-1 is recruited toward HIV-1 gp120/gp41-driven virus/cell and cell/cell contacts, associates with CD4, limits HIV-1-induced cell fusion and viral entry, and modulates gp120/gp41-triggered actin polymerization and PIP2 accumulation
ACTA2	155971	NP_579895.1	Envelope transmembrane glycoprotein gp41	inhibits	10556093,	The interaction of the long cytoplasmic tail of HIV-1 gp41 with the carboxy-terminal regulatory domain of p115-RhoGEF inhibits p115-mediated actin stress fiber formation and activation of serum response factor (SRF)
ACTA2	155030	NP_057850.1	Pr55(Gag)	co-localizes with	23260110,	HIV-1 Gag, ITK, and F-actin are located in overlapping and discrete regions of T cell-T cell contact sites
ACTA2	155030	NP_057850.1	Pr55(Gag)	interacts with	17360745, 17504171	Treatment of cells with actin-depolymerizing agents or tubulin polymerization inhibitors largely reduces the percentage of cells with capped HIV-1 Gag and Env, indicating an intact actin and tubulin cytoskeleton is required for efficient assembly of HIV-1
ACTA2	155030	NP_057850.1	Pr55(Gag)	interacts with	23260110,	Tec kinase chemical inhibitors diminish the recruitment of ITK to the plasma membrane perturbing HIV-1 Gag-ITK co-localization, disrupting F-actin polymerization, and inhibiting HIV-1 release and replication
ACTA2	155030	NP_057850.1	Pr55(Gag)	requires	19883584, 21917091, 22004035, 22989508	HIV-1 Gag assembly and budding occur through an actin-driven mechanism
ACTA2	155030	NP_579876.2	matrix	interacts with	9841925, 17411366, 19639585	The localization of the HIV-1 reverse transcription complex to actin microfilaments is mediated by the interaction of a reverse transcription complex component (HIV-1 Matrix) with actin, but not vimentin (intermediate filaments) or tubulin (microtubules)
ACTA2	155030	NP_579881.1	nucleocapsid	binds	23017337,	HIV-1 NC-like aggregates are associated with dsDNA synthesis by HIV-1 RT and appear to efficiently bind to F-actin filaments, a property that may be involved in targeting complexes to the nuclear envelope
ACTA2	155030	NP_579881.1	nucleocapsid	binds	8661406, 8892894, 9971772, 10049817, 10074138, 11709093, 12009869	Mature HIV-1 Nucleocapsid, as well as the nucleocapsid domain of the HIV-1 Gag polyprotein, binds filamentous actin resulting in incorporation of actin into virus particles and enhancement of cell motility
ACTA2	155348	NP_705926.1	retropepsin	cleaves	1540415,	Actin, one of the most abundant proteins of the cell, is hydrolyzed by the human immunodeficiency virus type 1 (HIV-1) protease during acute infection of cultured human T lymphocytes
ACTA2	155348	NP_705926.1	retropepsin	cleaves	1540415, 1907279, 1991513, 8997639	HIV-1 protease cleaves actin in vitro at amino acid residues 66-67, 94-95, and 126-127
ACTA2	155348	NP_705927.1	reverse transcriptase	co-localizes with	9841925,	The localization of the HIV-1 reverse transcription complex to actin microfilaments is mediated by the interaction of a reverse transcription complex component (HIV-1 Matrix) with actin, but not vimentin (intermediate filaments) or tubulin (microtubules)
ACTA2	155348	NP_705927.1	reverse transcriptase	interacts with	23017337,	HIV-1 NC-like aggregates are associated with dsDNA synthesis by HIV-1 RT and appear to efficiently bind to F-actin filaments, a property that may be involved in targeting complexes to the nuclear envelope
ACTA2	156110	NP_057857.2	Nef	co-localizes with	22721673,	HIV-1 Nef co-localizes with F-actin and reorganizes F-actin assembly in the cortical regions of human podocyte
ACTA2	156110	NP_057857.2	Nef	downregulates	23071112,	HIV-1 Nef inhibits CXCL12 induced chemotaxis in Jurkat cells, monocytes, and PBMCs, which leads to marked downregulation of F-actin accumulation in cells
ACTA2	156110	NP_057857.2	Nef	inhibits	20147394,	HIV-1 Nef requires a PAK2 recruitment motif (F195/191I) for inhibition of actin remodeling and induction of cofilin hyperphosphorylation
ACTA2	156110	NP_057857.2	Nef	inhibits	21923909,	HIV-1 Nef induces loss of F-actin assembly and inhibits retinoid receptor-mediated transcription
ACTA2	156110	NP_057857.2	Nef	relocalizes	27560372,	HIV-1 NA7 and SF2 Nefs relocalizes ACTA1 and ACTB (F-actin); dependent upon the C-terminal aspartic acids in Nef
ACTA2	155871	NP_057853.1	Tat	downregulates	16526095,	In Jurkat cells expressing HIV-1 Tat, decreased expression levels are found for basic cytoskeletal proteins such as actin, beta-tubulin, annexin, cofilin, gelsolin, and Rac/Rho-GDI complex
ACTA2	155871	NP_057853.1	Tat	downregulates	23811015, 23875777	Treatment of primary hippocampal neurons with HIV-1 Tat produces a significant early reduction in F-actin labeled puncta. The cysteine rich domain (residues 22-37) of Tat is required for Tat-mediated reduction of F-actin labeled puncta
ACTA2	155871	NP_057853.1	Tat	induces rearrangement of	14694110,	HIV-1 Tat induces actin cytoskeletal rearrangements through p21-activated kinase 1 (PAK1) and downstream activation of the endothelial NADPH oxidase, an effect that is lost by introduction of mutations into the Tat cysteine-rich or basic domains
ACTA2	155871	NP_057853.1	Tat	interacts with	24742657,	Treatment with cannabinoids inhibits HIV-1 Tat-enhanced attachment of U937 cells to collagen IV, laminin, or ECM1 proteins, which is linked to the cannabinoid receptor type 2 and the modulation of beta1-integrin and actin distribution
ACTA2	155871	NP_057853.1	Tat	regulated by	22465675, 23178941	Uptake of the HIV-1 Tat protein is regulated by arrangement of the actin cytoskeleton in epithelial cells
ACTA2	155807	NP_057852.2	Vpr	downregulates	23874603,	A stable-isotope labeling by amino acids in cell culture coupled with mass spectrometry-based proteomics identifies downregulation of actin, alpha 2 (ACTA2) expression by HIV-1 Vpr in Vpr transduced macrophages
RAN	155971	NP_579894.2	Envelope surface glycoprotein gp120	complexes with	23125841,	Tandem affinity purification and mass spectrometry analysis identify GTP binding protein RAN (RanGTP), HIV-1 Gag, Gag/Pol, gp120, and Nef incorporated into staufen1 RNP complexes isolated from HIV-1-expressing cells
RAN	155030	NP_057850.1	Pr55(Gag)	complexes with	23125841,	Tandem affinity purification and mass spectrometry analysis identify GTP binding protein RAN (RanGTP), HIV-1 Gag, Gag/Pol, gp120, and Nef incorporated into staufen1 RNP complexes isolated from HIV-1-expressing cells
RAN	155030	NP_057850.1	Pr55(Gag)	regulated by	9562972,	Ran terminates the nuclear import of the Matrix protein of HIV-1 Gag by directly binding to karyopherin beta and disassembling the import complex
RAN	155030	NP_579880.1	capsid	inhibited by	23097435,	RanGTP inhibits the ability of TNPO3 to stimulate the uncoating of HIV-1 CA cores
RAN	155030	NP_579876.2	matrix	regulated by	9562972,	Ran terminates the nuclear import of HIV-1 Matrix by directly binding to karyopherin beta and disassembling the import complex

RAN	155348	NP_057849.4	Gag-Pol	complexes with	23125841,	Tandem affinity purification and mass spectrometry analysis identify GTP binding protein RAN (RanGTP), HIV-1 Gag, Gag/Pol, gp120, and Nef incorporated into staufen1 RNP complexes isolated from HIV-1-expressing cells
RAN	155348	NP_705928.1	integrase	inhibited by	23878195,	The binding between HIV-1 IN and TNPO3 is inhibited by RanGTP in a dose-dependent manner, leading to a TNPO3-RanGTP complex formation
RAN	156110	NP_057857.2	Nef	complexes with	23125841,	Tandem affinity purification and mass spectrometry analysis identify GTP binding protein RAN (RanGTP), HIV-1 Gag, Gag/Pol, gp120, and Nef incorporated into staufen1 RNP complexes isolated from HIV-1-expressing cells
RAN	155908	NP_057854.1	Rev	binds	25486595,	A dimeric CRM1-RanGTP complex binds a Rev-RRE complex to export the Rev-RRE complex from the nuclear to the cytoplasm in cells
RAN	155908	NP_057854.1	Rev	binds	9837918, 10518602, 18508616, 19149559, 21358275, 22355797, 22783232, 24530126, 25486594, 25486595, 25564443, 25723178	the GTP bound form of Ran (RanGTP) binds to a preformed Rev-CRM1 (exportin 1) complex to mediate nuclear export of HIV-1 mRNA
RAN	155908	NP_057854.1	Rev	binds	9837918, 18508616, 19149559, 22783232	binding of the GTP bound form of Ran (RanGTP) to a preformed Rev-CRM1 complex is linked to an interaction of RanGTP with the nuclear export signal (NES) of Rev (amino acids 75-83)
RAN	155908	NP_057854.1	Rev	enhanced by	12134013, 18508616	Rev-Rev interactions (multimerization) are enhanced by RanGTP
RAN	155908	NP_057854.1	Rev	interacts with	22174317,	HIV-1 Rev interacting protein, RAN, is identified by the in-vitro binding experiments involving cytosolic or nuclear extracts from HeLa cells
RAN	155871	NP_057853.1	Tat	interacts with	19454010,	Interaction of HIV-1 Tat with RAN in T-cells is identified by a proteomic strategy based on affinity chromatography
EIF5A	155348	NP_705926.1	retropepsin	cleaves	22944692,	Positional proteomics analysis identifies the cleavage of human eukaryotic translation initiation factor 5A (EIF5A) at amino acid residues 5-6, 96-97, 97-98, and 100-101 by the HIV-1 protease
EIF5A	156110	NP_057857.2	Nef	downregulates	25874870,	HIV-1 Nef downregulates the expression of eukaryotic translation initiation factor 5A (EIF5A) protein in Nef-transfected SupT1 cells
EIF5A	155908	NP_057854.1	Rev	binds	8253832,	eIF-5A interacts with the activation domain of Rev (amino acids 75-83)
EIF5A	155908	NP_057854.1	Rev	binds	9285100, 17578650, 19149558, 21360055	eIF-5A binds Rev Response Element (RRE) RNA, an interaction likely to be involved in its association with Rev during nuclear export
EIF5A	155908	NP_057854.1	Rev	binds	9465063, 16354571	eIF-5A interacts with the ribosomal protein L5 during its Rev nuclear export activity
EIF5A	155908	NP_057854.1	Rev	interacts with	10381392, 11238447, 19149558, 21360055	eIF-5A directly interacts with CRM1 and mediates its binding to Rev, an effect that is required for nuclear export of Rev
EIF5A	155908	NP_057854.1	Rev	interacts with	11238447,	eIF-5A interacts with nucleoporins CAN/nup214, nup153, nup98, and nup62 as well as nuclear actin during the nuclear export of Rev
EIF5A	155908	NP_057854.1	Rev	interacts with	7971969, 9285095, 16354571, 16515720, 17578650, 19149558	eIF-5A is a hypusine-containing protein required for Rev-mediated nuclear export of HIV-1 mRNA
TUBA1B	155971	NP_579894.2	Envelope surface glycoprotein gp120	induces acetylation of	15103018, 16148047	The binding of HIV-1 gp120 to CD4+-permissive cells increases the level of acetylated alpha-tubulin in a CD4-dependent manner; overexpression of Histone Deacetylase 6 (HDAC6) inhibits the acetylation of alpha-tubulin and prevents HIV-1-cell fusion
TUBA1B	155971	NP_057856.1	Envelope surface glycoprotein gp160, precursor	interacts with	17360745,	Treatment of cells with actin-depolymerizing agents or tubulin polymerization inhibitors largely reduces the percentage of cells with capped HIV-1 Gag and Env, indicating an intact actin and tubulin cytoskeleton is required for efficient assembly of HIV-1
TUBA1B	155030	NP_057850.1	Pr55(Gag)	interacts with	17360745,	Treatment of cells with actin-depolymerizing agents or tubulin polymerization inhibitors largely reduces the percentage of cells with capped HIV-1 Gag and Env, indicating an intact actin and tubulin cytoskeleton is required for efficient assembly of HIV-1
TUBA1B	155348	NP_705928.1	integrase	interacts with	21167302,	Co-immunoprecipitation shows interaction of HIV-1 IN with alpha-tubulin
TUBA1B	155348	NP_705926.1	retropepsin	cleaves	22944692,	Positional proteomics analysis identifies the cleavage of human tubulin, alpha 1b (TUBA1B) at amino acid residues 67-68 and 202-203 by the HIV-1 protease
TUBA1B	155908	NP_057854.1	Rev	depolymerizes	10908577,	Rev acts to depolymerize microtubules that are formed by tubulin, an effect that is observed during HIV-1 infection
TUBA1B	155908	NP_057854.1	Rev	interacts with	22174317,	HIV-1 Rev interacting protein, TUBA1B, is identified by the in-vitro binding experiments involving cytosolic or nuclear extracts from HeLa cells
TUBA1B	155871	NP_057853.1	Tat	binds	12486001, 15331610	HIV-1 Tat (amino acids 36-39) binds tubulin alpha/beta dimers and polymerized microtubules leading to the alteration of microtubule dynamics and activation of a mitochondria-dependent apoptotic pathway that is facilitated by the Bcl-2 relative Bim
TUBA1B	155871	NP_057853.1	Tat	enhances polymerization of	15691386, 15698476, 18613978	HIV-1 Tat (specifically, amino acids 38-72), enhances tubulin polymerization and triggers the mitochondrial pathway to induce T cell apoptosis as shown in vitro by the release of cytochrome c from isolated mitochondria
TUBA1B	155871	NP_057853.1	Tat	modulates	23826228, 25328666	HIV-1 Tat K29A, K50R, and K51R lysine mutations downregulate the proportion of soluble tubulin in cells, while the majority of other lysine mutations upregulate the percentage of soluble tubulin compared with the wild-type
TUBA1B			HIV-1 virus replication	enhanced by expression of human gene	19460752,	Knockdown of tubulin, alpha 1b (TUBA1B) by shRNA library screening inhibits HIV-1 replication in cultured Jurkat T-cells
LGALS3BP	155971	NP_579894.2	Envelope surface glycoprotein gp120	downregulates	24156545,	The expression of 90K/LGALS3BP downregulates the relative amounts of mature gp120/gp41, whereas it upregulates the relative levels of uncleaved gp160 precursor inhibiting incorporation of the viral gp120/gp41 glycoproteins into progeny virions
LGALS3BP	155971	NP_579894.2	Envelope surface glycoprotein gp120	inhibited by	24156545,	The two central protein-binding domains (residues 127-409) of 90K/LGALS3BP are required for inhibition of gp160 processing and incorporation of the viral gp120/gp41 glycoproteins into progeny virions
LGALS3BP	155971	NP_057856.1	Envelope surface glycoprotein gp160, precursor	inhibited by	24156545,	The two central protein-binding domains (residues 127-409) of 90K/LGALS3BP are required for inhibition of gp160 processing and incorporation of the viral gp120/gp41 glycoproteins into progeny virions
LGALS3BP	155971	NP_057856.1	Envelope surface glycoprotein gp160, precursor	upregulates	24156545,	The expression of 90K/LGALS3BP downregulates the relative amounts of mature gp120/gp41, whereas it upregulates the relative levels of uncleaved gp160 precursor inhibiting incorporation of the viral gp120/gp41 glycoproteins into progeny virions
LGALS3BP	155971	NP_579895.1	Envelope transmembrane glycoprotein gp41	downregulates	24156545,	The expression of 90K/LGALS3BP downregulates the relative amounts of mature gp120/gp41, whereas it upregulates the relative levels of uncleaved gp160 precursor inhibiting incorporation of the viral gp120/gp41 glycoproteins into progeny virions
LGALS3BP	155971	NP_579895.1	Envelope transmembrane glycoprotein gp41	inhibited by	23156545,	The two central protein-binding domains (residues 127-409) of 90K/LGALS3BP are required for inhibition of gp160 processing and incorporation of the viral gp120/gp41 glycoproteins into progeny virions
LGALS3BP	155030	NP_057850.1	Pr55(Gag)	binds	27604950,	HIV-1 Gag binds to LGALS3BP (M2BP)
LGALS3BP	155030	NP_057850.1	Pr55(Gag)	inhibited by	27604950,	HIV-1 Gag trafficking to the plasma membrane is inhibited by LGALS3BP (M2BP) and the inhibition by LGALS3BP is dependent upon vimentin
LGALS3BP	155348	NP_789740.1	Pol	interacts with	22190034,	HIV-1 Pol is identified to have a physical interaction with lectin, galactoside-binding, soluble, 3 binding protein (LGALS3BP) in human HEK293 and/or Jurkat cell lines by using affinity tagging and purification mass spectrometry analyses
LGALS3BP			HIV-1 virus replication	inhibited by expression of human gene	24156545,	Knockdown of 90K/LGALS3BP by siRNA enhances replication and infectivity of HIV-1 in TZM-bl cells and primary macrophages
LGALS3BP			HIV-1 virus replication	inhibited by expression of human gene	27604950,	HIV-1 replication is inhibited by LGALS3BP (M2BP) overexpression in 293HEK cells
ALCAM			HIV-1 virus replication	inhibited by expression of human gene	22082156,	Knockdown of activated leukocyte cell adhesion molecule (ALCAM) by siRNA enhances the early stages of HIV-1 replication in HeLa-CD4 cells infected with viral pseudotypes HIV89.6R and HIV8.2N
CFHR5			HIV-1 virus replication	enhanced by expression of human gene	18976975,	Knockdown of complement factor H-related 5 (CFHR5) by siRNA inhibits HIV-1 replication in HeLa P4/R5 cells
FETUB	155971	NP_057856.1	Envelope surface glycoprotein gp160, precursor	binds	1284814,	HIV-1 gp160 binds to the natural glycoprotein fetuin

HPSE	155871	NP_057853.1	Tat	interacts with	15264223,	Treatment of synaptosomes with heparanase and HIV-1 Tat increases Tat-induced oxidative stress, which indicates the requirement of Tat interaction with neuronal membranes to induce oxidative damage
CHEK1	155807	NP_057852.2	Vpr	activates	12738771, 16306615, 17210576, 19275579, 24158819, 24795708	HIV-1 Vpr activates ATR, resulting in phosphorylation of Chk1 and activation of the ATR-mediated DNA damage response
CHEK1	155807	NP_057852.2	Vpr	induces phosphorylation of	17210576, 19275579, 20609246	ATR-induced phosphorylation of Chk1-Ser(345) requires PP2A only when Vpr activates ATR
CHEK1	155807	NP_057852.2	Vpr	induces phosphorylation of	20609246, 24744753	Phosphorylation of Chk1 at position Ser345 is required for HIV-1 Vpr-induced G2/M arrest, possibly through signaling of DNA re-replication via Cdt1
NRP1	156110	NP_057857.2	Nef	upregulates	18443354,	HIV-1 Nef upregulates VEGFR2 and its co-receptor neuropilin-1 and downregulates the expression of semaphorin 3a in podocytes
NRP1			HIV-1 virus replication	enhanced by expression of human gene	19460752,	Knockdown of neuropilin 1 (NRP1) by shRNA library screening inhibits HIV-1 replication in cultured Jurkat T-cells
TGOLN2	155030	NP_057850.1	Pr55(Gag)	co-localizes with	21563830,	HIV-1 Gag co-localizes with Rab9 and TGN38 in endosomes and trans-Golgi compartments
TGOLN2	155945	NP_057855.1	Vpu	co-localizes with	20386718, 20926557, 21607084, 21900423	Vpu interferes with tetherin trafficking to the cell-surface and causes a relocalization of the cellular tetherin with a TGN marker TGN46 in the TGN
TGOLN2	155945	NP_057855.1	Vpu	co-localizes with	24910430,	HIV-1 Vpu co-localizes with the trans-golgi network protein TGN46 in CD4+ T cells
LDHA	155971	NP_579894.2	Envelope surface glycoprotein gp120	complexes with	23125841,	Tandem affinity purification and mass spectrometry analysis identify lactate dehydrogenase A (LDHA), HIV-1 Gag, Gag/Pol, gp120, and Nef incorporated into staufen1 RNP complexes isolated from HIV-1-expressing cells
LDHA	155971	NP_579894.2	Envelope surface glycoprotein gp120	induces release of	11125887,	HIV-1 gp120/41 (SFVenvBX08)-expressing microglia exhibit a 170% increase in lactate dehydrogenase (LDH) release
LDHA	155971	NP_579895.1	Envelope transmembrane glycoprotein gp41	induces release of	11582518,	A lentivirus lytic peptide 1 (LLP-1) corresponding to the carboxyl terminus of HIV-1 gp41 induces a significant lactate dehydrogenase (LDH, a marker of cell death) release from human neuronal and glial cell lines
LDHA	155030	NP_057850.1	Pr55(Gag)	complexes with	23125841,	Tandem affinity purification and mass spectrometry analysis identify lactate dehydrogenase A (LDHA), HIV-1 Gag, Gag/Pol, gp120, and Nef incorporated into staufen1 RNP complexes isolated from HIV-1-expressing cells
LDHA	155348	NP_057849.4	Gag-Pol	complexes with	23125841,	Tandem affinity purification and mass spectrometry analysis identify lactate dehydrogenase A (LDHA), HIV-1 Gag, Gag/Pol, gp120, and Nef incorporated into staufen1 RNP complexes isolated from HIV-1-expressing cells
LDHA	156110	NP_057857.2	Nef	complexes with	23125841,	Tandem affinity purification and mass spectrometry analysis identify lactate dehydrogenase A (LDHA), HIV-1 Gag, Gag/Pol, gp120, and Nef incorporated into staufen1 RNP complexes isolated from HIV-1-expressing cells
LDHA	155871	NP_057853.1	Tat	downregulates	15710247,	Upregulation of actin, heat shock protein 90 and mitochondrial single-stranded DNA binding protein, and downregulation of lactate dehydrogenase are identified in human astrocytes expressing Tat
LDHA	155871	NP_057853.1	Tat	upregulates	23025307,	HIV-1 Tat upregulates lactate dehydrogenase A (LDHA) expression in Jurkat T-cells
GSR	155348	NP_705926.1	retropepsin	cleaves	22944692,	Positional proteomics analysis identifies the cleavage of human glutathione reductase, mitochondria (GSR) at amino acid residues 67-68 by the HIV-1 protease
F13A1	155871	NP_057853.1	Tat	downregulates	24667918,	Microarray analysis indicates HIV-1 Tat-induced downregulation of coagulation factor XIII, A1 polypeptide (F13A1) in primary human brain microvascular endothelial cells
CA2			HIV-1 virus replication	enhanced by expression of human gene	18976975,	Knockdown of carbonic anhydrase II (CA2) by siRNA inhibits HIV-1 replication in HeLa P4/R5 cells
SERPINA3	155971	NP_579894.2	Envelope surface glycoprotein gp120	upregulates	23867815,	HIV-1 gp120-treated vaginal epithelial cells show upregulation of serpin peptidase inhibitor, clade A, member 3 (SERPINA3) expression as compared to untreated control
SERPINA3	155871	NP_057853.1	Tat	upregulates	24667918,	Microarray analysis indicates HIV-1 Tat-induced upregulation of serpin peptidase inhibitor, clade A, member 3 (SERPINA3) in primary human brain microvascular endothelial cells
SERPINA3	155971	NP_579894.2	Envelope surface glycoprotein gp120	upregulates	23867815,	HIV-1 gp120-treated vaginal epithelial cells show upregulation of serpin peptidase inhibitor, clade A, member 3 (SERPINA3) expression as compared to untreated control
SERPINA3	155871	NP_057853.1	Tat	upregulates	24667918,	Microarray analysis indicates HIV-1 Tat-induced upregulation of serpin peptidase inhibitor, clade A, member 3 (SERPINA3) in primary human brain microvascular endothelial cells
A2M	155971	NP_579895.1	Envelope transmembrane glycoprotein gp41	downregulates	23383108,	A synthetic peptide corresponding to the immunosuppressive domain (amino acids 574-592) of HIV-1 gp41 downregulates the expression of alpha-2-macroglobulin (A2M) in peptide-treated PBMCs
A2M	155348	NP_705926.1	retropepsin	cleaves	1724156, 7524416, 7690356	The cleavage site of alpha 2-Macroglobulin by HIV-1 protease is the Phe684-Tyr685 bond
A2M	155871	NP_057853.1	Tat	inhibits	11100124,	Binding of HIV-1 Tat to LRP inhibits neuronal binding, uptake and degradation of physiological ligands for LRP, including alpha2-macroglobulin, apolipoprotein E4, amyloid precursor and amyloid beta-protein
C3	155971	NP_579894.2	Envelope surface glycoprotein gp120	binds	7590866, 7642209, 7893437, 7911492	Complement proteins C4, C3d, C5b-9, and properdin bind to HIV-1 gp120-coated CD4+ T cells of healthy individuals when incubated in autologous serum
C3	155971	NP_579894.2	Envelope surface glycoprotein gp120	binds	7590866, 8630395	Amino acid residues 100-129, 161-190, 231-250, 301-328, 410-449, and 470-499 of HIV-1 gp120 are involved in its binding to C3
C3	155971	NP_579894.2	Envelope surface glycoprotein gp120	binds	8630395,	A synthetic peptide covering positions 233-251 of the HIV-1 gp120 protein binds to complement proteins C3, C4, C5, C9, and properdin
C3	155971	NP_579894.2	Envelope surface glycoprotein gp120	cleaves	8471312,	Complexes of recombinant HIV-1 gp120 with anti-HIV-1 antibodies cleave C3 and present generated C3 fragments on the cell surface
C3	155971	NP_579894.2	Envelope surface glycoprotein gp120	interacts with	7535292,	Inhibition of DAF or use of factor H depleted sera significantly increases C3 deposition on recombinant HIV-1 gp120 coated CD4 cells
C3	155971	NP_579894.2	Envelope surface glycoprotein gp120	interacts with	7893437,	Preincubation of HIV-1 gp41 with either factor H or properdin, and of HIV-1 gp120 with C3b or C4b affect the interaction between HIV-1 gp41 and gp120
C3	155971	NP_057856.1	Envelope surface glycoprotein gp160, precursor	upregulates	8471312, 9544576	Complement component 3 (C3) production is upregulated by HIV-1 gp160
C3	156110	NP_057857.2	Nef	upregulates	11884542, 19878567	HIV-1 induces the upregulation of complement factor C3 in astrocytes and neurons through signaling pathways that involve protein kinase C and adenylate cyclase activation, which is an effect that may contribute to the pathogenesis of AIDS in the brain
C3	155871	NP_057853.1	Tat	upregulates	24667918,	Microarray analysis indicates HIV-1 Tat-induced upregulation of complement component 3 (C3) in primary human brain microvascular endothelial cells
SPTA1	155348	NP_705926.1	retropepsin	cleaves	12119179,	A number of focal adhesion plaque proteins are specifically cleaved by HIV-1 protease, including fimbrin, focal adhesion plaque kinase (FAK), talin, and, to a lesser extent, filamin, spectrin and fibronectin
SPTA1	155348	NP_705926.1	retropepsin	cleaves	1991513, 8997639	HIV-1 protease cleaves alpha-spectrin in vitro at amino acid positions 1352, 1376, and 1697
SLC4A1			HIV-1 virus replication	enhanced by expression of human gene	19460752,	Knockdown of solute carrier family 4, anion exchanger, member 1 (erythrocyte membrane protein band 3, Diego blood group; SLC4A1) by shRNA library screening inhibits HIV-1 replication in cultured Jurkat T-cells
APOH	155030	NP_057850.1	Pr55(Gag)	binds	8989432,	Binding of apolipoprotein H to HIV-1 Gag protein p18 as well as to the Gag p55 polyprotein has been demonstrated in vitro
FN1	155971	NP_579894.2	Envelope surface glycoprotein gp120	binds	1875953, 8173552	Fibronectin, which is present in submandibular saliva, binds to HIV-1 gp120/160 and enhances the interaction of C1q with gp120/160
FN1	155971	NP_579894.2	Envelope surface glycoprotein gp120	binds	8952048, 9443108, 10706716	HIV-1 gp120 binds to fibronectin (FN) present on the surface of platelets; the specificity of this binding is confirmed by the inhibition obtained by pretreating platelets with anti-FN antibodies

FN1	155971	NP_579894.2	Envelope surface glycoprotein gp120	induces phosphorylation of	22241990,	HIV-1 gp120 induces phosphorylation of fibronectin and enhances a physical association between fibronectin and Robo4 in human lymphatic endothelial cells
FN1	155971	NP_057856.1	Envelope surface glycoprotein gp160, precursor	binds	1875953, 9443108	HIV-1 gp160 and gp120 specifically recognize the C-terminal heparin-binding domain of fibronectin (Fn) and this binding inhibits the interaction of gp160/gp120 with soluble CD4
FN1	155971	NP_579895.1	Envelope transmembrane glycoprotein gp41	binds	8173552,	Fibronectin (FN) binds to HIV-1 glycoproteins, including gp41 and gp120; preincubation with antibodies against FN abolishes this binding
FN1	155348	NP_705926.1	retropepsin	cleaves	1959621, 8997639, 12119179	The cell-associated protein fibronectin (A-chain) is specifically cleaved in vitro by HIV-1 protease
FN1	156110	NP_057857.2	Nef	upregulates	10451539, 11180285	Exogenous HIV-1 Nef upregulates fibronectin (FN) expression in MT4 and H9 T-cell lines
FN1	155871	NP_057853.1	Tat	competes with	7690138,	HIV-1 Tat competes with fibronectin for binding to integrins
FN1	155871	NP_057853.1	Tat	modulated by	9339851, 9626063	Fibronectin modulates the effects of HIV-1 Tat on endothelial cells and murine Kaposi's sarcoma-like cells
FN1	155871	NP_057853.1	Tat	upregulates	1409674, 8599839, 11311202	HIV-1 Tat upregulates fibronectin expression in salivary gland cell lines, thymic epithelial cells, and glioblastoma cells
FN1	155971	NP_579894.2	Envelope surface glycoprotein gp120	binds	1875953, 8173552	Fibronectin, which is present in submandibular saliva, binds to HIV-1 gp120/160 and enhances the interaction of C1q with gp120/160
FN1	155971	NP_579894.2	Envelope surface glycoprotein gp120	binds	8952048, 9443108, 10706716	HIV-1 gp120 binds to fibronectin (FN) present on the surface of platelets; the specificity of this binding is confirmed by the inhibition obtained by pretreating platelets with anti-FN antibodies
FN1	155971	NP_579894.2	Envelope surface glycoprotein gp120	induces phosphorylation of	22241990,	HIV-1 gp120 induces phosphorylation of fibronectin and enhances a physical association between fibronectin and Robo4 in human lymphatic endothelial cells
FN1	155971	NP_057856.1	Envelope surface glycoprotein gp160, precursor	binds	1875953, 9443108	HIV-1 gp160 and gp120 specifically recognize the C-terminal heparin-binding domain of fibronectin (Fn) and this binding inhibits the interaction of gp160/gp120 with soluble CD4
FN1	155971	NP_579895.1	Envelope transmembrane glycoprotein gp41	binds	8173552,	Fibronectin (FN) binds to HIV-1 glycoproteins, including gp41 and gp120; preincubation with antibodies against FN abolishes this binding
FN1	155348	NP_705926.1	retropepsin	cleaves	1959621, 8997639, 12119179	The cell-associated protein fibronectin (A-chain) is specifically cleaved in vitro by HIV-1 protease
FN1	156110	NP_057857.2	Nef	upregulates	10451539, 11180285	Exogenous HIV-1 Nef upregulates fibronectin (FN) expression in MT4 and H9 T-cell lines
FN1	155871	NP_057853.1	Tat	competes with	7690138,	HIV-1 Tat competes with fibronectin for binding to integrins
FN1	155871	NP_057853.1	Tat	modulated by	9339851, 9626063	Fibronectin modulates the effects of HIV-1 Tat on endothelial cells and murine Kaposi's sarcoma-like cells
FN1	155871	NP_057853.1	Tat	upregulates	1409674, 8599839, 11311202	HIV-1 Tat upregulates fibronectin expression in salivary gland cell lines, thymic epithelial cells, and glioblastoma cells
PPBP	155348	NP_705926.1	retropepsin	cleaves	10419831,	Connective tissue-activating peptide (CTAPIII) and neutrophil-activating peptide 2 (NAP/2) are generated by digestion of a ubiquitin-CTAPIII conjugate with yeast ubiquitin C-terminal hydrolase (YUH1) and HIV protease, respectively
PPBP	156110	NP_057857.2	Nef	incorporates	27211553,	HIV-1 Nef specifically incorporates CSF2, PPBP (NAP2), CCL5, TNF, FAS, CXCL1, IL12B, MIF and OSM into plasma extracellular vesicles from HIV-1 infected patient samples
PF4	155971	NP_579894.2	Envelope surface glycoprotein gp120	binds	22645343, 26847431	CXCL4 directly interacts with HIV-1 gp120 and the CXCL4-binding site is located within the gp120 outer domain (residues 350-455) proximal to the CD4-binding site
PF4	155971	NP_579894.2	Envelope surface glycoprotein gp120	binds	26847431,	HIV-1 JRFL Env (gp120) binds specifically to polystyrene-immobilized PF4 (CXCL4) and CCL5 (RANTES) as measured through ELISA
PF4			HIV-1 virus replication	enhanced by expression of human gene	26847431,	HIV-1 CH077 virus entry is enhanced by high concentrations of PF4 (CXCR4) in MAGI-R5, SupT1-R5, Jurkat-R5, and primary CD4+ T cells
PF4			HIV-1 virus replication	enhanced by expression of human gene	26847431,	In single-round infection assays, HIV-1 NL4-3 psuedotyped with Env proteins from subtypes B and C entry is enhanced by high concentrations of tetrameric PF4 (CXCR4) in MAGI-R5 cells
PF4			HIV-1 virus replication	inhibited by expression of human gene	26847431,	HIV-1 CH077 virus entry is inhibited by low concentrations of PF4 (CXCR4) in MAGI-R5, SupT1-R5, Jurkat-R5, and primary CD4+ T cells
PF4			HIV-1 virus replication	inhibited by expression of human gene	26847431,	In single-round infection assays, HIV-1 NL4-3 pseudotyped with Env proteins from subtypes B and C entry is enhanced by high concentrations of tetrameric PF4 (CXCR4) in MAGI-R5 cells
PF4	155971	NP_579894.2	Envelope surface glycoprotein gp120	binds	22645343, 26847431	CXCL4 directly interacts with HIV-1 gp120 and the CXCL4-binding site is located within the gp120 outer domain (residues 350-455) proximal to the CD4-binding site
PF4	155971	NP_579894.2	Envelope surface glycoprotein gp120	binds	26847431,	HIV-1 JRFL Env (gp120) binds specifically to polystyrene-immobilized PF4 (CXCL4) and CCL5 (RANTES) as measured through ELISA
PF4			HIV-1 virus replication	enhanced by expression of human gene	26847431,	HIV-1 CH077 virus entry is enhanced by high concentrations of PF4 (CXCR4) in MAGI-R5, SupT1-R5, Jurkat-R5, and primary CD4+ T cells
PF4			HIV-1 virus replication	enhanced by expression of human gene	26847431,	In single-round infection assays, HIV-1 NL4-3 psuedotyped with Env proteins from subtypes B and C entry is enhanced by high concentrations of tetrameric PF4 (CXCR4) in MAGI-R5 cells
PF4			HIV-1 virus replication	inhibited by expression of human gene	26847431,	HIV-1 CH077 virus entry is inhibited by low concentrations of PF4 (CXCR4) in MAGI-R5, SupT1-R5, Jurkat-R5, and primary CD4+ T cells
PF4			HIV-1 virus replication	inhibited by expression of human gene	26847431,	In single-round infection assays, HIV-1 NL4-3 pseudotyped with Env proteins from subtypes B and C entry is enhanced by high concentrations of tetrameric PF4 (CXCR4) in MAGI-R5 cells
TFRC	155971	NP_579894.2	Envelope surface glycoprotein gp120	downregulates	9341758,	Treatment of CD4+ T lymphocytes with HIV-1 gp120 before anti-CD3 stimulation impedes cell cycle progression as measured by reduced CD71 expression
TFRC	155971	NP_579894.2	Envelope surface glycoprotein gp120	upregulates	15784911,	CD4+ T cells infected with CCR5-tropic HIV-1 have significantly higher levels of activation-marker expression (e.g. CD25, CD71 and HLA-DR) than CD4+ T lymphocytes infected with CXCR4-tropic HIV-1
TFRC	155971	NP_057856.1	Envelope surface glycoprotein gp160, precursor	interacts with	23861967,	Immunoblot of sucrose fractions to lipid raft marker Flotillin-1 and non-lipid raft marker TfR shows that HIV-1 Gag and Env interact with both Flotillin-1 and TfR in cells
TFRC	155030	NP_057850.1	Pr55(Gag)	interacts with	23861967,	Immunoblot of sucrose fractions to lipid raft marker Flotillin-1 and non-lipid raft marker TfR shows that HIV-1 Gag and Env interact with both Flotillin-1 and TfR in cells
TFRC	156110	NP_057857.2	Nef	downregulates	10451539, 21922073	Treatment of uninfected and acutely or chronically infected MT-4 and H9 T-cells with exogenous HIV-1 Nef downregulates the expression of CD71
TFRC	156110	NP_057857.2	Nef	downregulates	25275127,	Both HIV-1 Nef and Vpu downregulate the cell surface expression of transferrin receptor (TFRC, TfR, p90, CD71)
TFRC	156110	NP_057857.2	Nef	modulates	15569681,	HIV-1 Nef modulates the trafficking of transferrin receptor (TfR), the prototypical recycling surface protein, with the dileucine motif (amino acids 164-5) in Nef being required for this alteration of TfR recycling
TFRC	155871	NP_057853.1	Tat	interacts with	25496916,	Transferrin receptor protein (TFRC) is identified to interact with HIV-1 Tat mutant Nullbasic in HeLa cells by LC MS/MS
TFRC	155459	NP_057851.1	Vif	interacts with	27375898,	HIV-1 Vif interacts with TFRC
TFRC	155807	NP_057852.2	Vpr	upregulates	23874603,	A stable-isotope labeling by amino acids in cell culture coupled with mass spectrometry-based proteomics identifies upregulation of transferrin receptor 1 (TFR1) expression by HIV-1 Vpr in Vpr transduced macrophages
TFRC	155945	NP_057855.1	Vpu	co-localizes with	20880565,	HIV-1 Vpu predominantly co-localizes to the detergent-soluble fractions with a non-raft protein TfR and partially partitions to the detergent-resistant membrane fractions with a raft protein flotillin 1
TFRC	155945	NP_057855.1	Vpu	downregulates	25275127,	Both HIV-1 Nef and Vpu downregulate the cell surface expression of transferrin receptor (TFRC, TfR, p90, CD71)
TF	155030	NP_579880.1	capsid	co-localizes with	26623655,	HIV-1 CA colocalizes with TF in follicular dendritic cells from lymph nodes of HIV infected patients on antiretroviral therapy
TF	155030	NP_579876.2	matrix	associates with	24830293,	HIV-1 MA associates with transferrin in recycling endosomes in human vaginal epithelial cells
TF	156110	NP_057857.2	Nef	co-localizes with	23372701,	HIV-1 Nef co-localizes with CTLA-4 in early and recycling endosomes with transferrin marker protein in HeLa cells

TF	155871	NP_057853.1	Tat	upregulates	24667918,	Microarray analysis indicates HIV-1 Tat-induced upregulation of transferrin (TF) in primary human brain microvascular endothelial cells
TF	155030	NP_579880.1	capsid	co-localizes with	26623655,	HIV-1 CA colocalizes with TF in follicular dendritic cells from lymph nodes of HIV infected patients on antiretroviral therapy
TF	155030	NP_579876.2	matrix	associates with	24830293,	HIV-1 MA associates with transferrin in recycling endosomes in human vaginal epithelial cells
TF	156110	NP_057857.2	Nef	co-localizes with	23372701,	HIV-1 Nef co-localizes with CTLA-4 in early and recycling endosomes with transferrin marker protein in HeLa cells
TF	155871	NP_057853.1	Tat	upregulates	24667918,	Microarray analysis indicates HIV-1 Tat-induced upregulation of transferrin (TF) in primary human brain microvascular endothelial cells
LTF	155971	NP_579894.2	Envelope surface glycoprotein gp120	binds	9781360, 11675140, 12103434, 14717698, 15222480, 15709021, 16261253, 16928883, 18183929, 21847071	Native lactoferrin (LF) and acylated LF from milk strongly bind to the V3 domain of the HIV-1 envelope protein gp120, resulting in inhibition of the virus-cell fusion and entry of the virus in CD4+ cells
LTF	155348	NP_705927.1	reverse transcriptase	inhibited by	25445609,	A peptide derived from human lactoferricin inhibits HIV-1 RT activity in a dose-dependent manner
LTF			HIV-1 virus replication	inhibited by expression of human gene	27182834,	HIV-1 is inhibited by TNC (tenascin C), MUC1, and LTF (lactoferrin) as shown through neutralization assays in TZM-bl cells
ANG			HIV-1 virus replication	incorporates expression of human gene	27211553,	HIV-1 infected clinical samples have plasma extracellular vesicles that contain elevated CCL1 (I309), IGFBP1, CCL5 (RANTES), GMCSF, ANG (Angiogenin), ADIPOQ (ACRP30), CSF3 (GCSF), CXCL1, ICAM1, IL2RA, IL6R, TNFRSF1A, and TIMP1 compared to healthy donors
VTN	155971	NP_579894.2	Envelope surface glycoprotein gp120	interacts with	7522689,	Interaction of S-protein (vitronectin) with HIV-1 gp120 and CD4 is mediated by heparin
VTN	155971	NP_579894.2	Envelope surface glycoprotein gp120	upregulates	23867815,	HIV-1 gp120-treated vaginal epithelial cells show upregulation of vitronectin (VTN) expression as compared to untreated control
VTN	155871	NP_057853.1	Tat	competes with	7682219, 7690138	HIV-1 Tat competes with vitronectin for binding to integrins
CAT	155971	NP_057856.1	Envelope surface glycoprotein gp160, precursor	inhibited by	14975589,	Free radical scavengers such as superoxide dismutase (SOD), dimethylthiourea (DMTU) and catalase attenuate morphine and gp160-induced human monocyte apoptosis
CAT	155871	NP_057853.1	Tat	inhibits	24480751,	Exposure to HIV-1 clade B Tat protein has a greater inhibition of GSS, GPx1, SOD1, and CAT expression compared with exposure to clade C Tat protein in monocyte-derived immature dendritic cells
CAT	155871	NP_057853.1	Tat	upregulates	22556393,	HIV-1 Tat increases catalase and glutathione peroxidase 1 (GPX1) activities in human cardiac myocyte
CAT	155871	NP_057853.1	Tat	upregulates	26895301,	HIV-1 Tat upregulates NQO1, CAT, SOD1, SOD2, and HMOX1 (HO1) mRNA levels in SH-SY5Y cells
ANXA1	155971	NP_579894.2	Envelope surface glycoprotein gp120	downregulates	17676665,	HIV-1 gp120 upregulates the expression of annexin A4 and V in umbilical cord blood mononuclear cells and T-cell lines but downregulates the expression of annexin A1 in umbilical cord blood mononuclear cells
SOD2	155971	NP_057856.1	Envelope surface glycoprotein gp160, precursor	inhibited by	14975589,	Free radical scavengers such as superoxide dismutase (SOD), dimethylthiourea (DMTU) and catalase attenuate morphine and gp160-induced human monocyte apoptosis
SOD2	155871	NP_057853.1	Tat	downregulates	10393559,	HIV-1 Tat inhibits Tip60 histone-acetyltransferase activity and abolishes Tip60-dependent transcriptional activity of the Mn-SOD promoter, resulting in downregulation of Mn-SOD
SOD2	155871	NP_057853.1	Tat	downregulates	15706661,	HIV-1 Tat downregulates SOD2 expression by interacting with Sp1 and Sp3 to increase the Sp3-containing complexes on the basal SOD2 promoter
SOD2	155871	NP_057853.1	Tat	downregulates	15869407,	HIV-1 Tat reduces the expression of MnSOD in several cell types
SOD2	155871	NP_057853.1	Tat	downregulates	7859743, 8395050, 9110146, 9882443	HIV-1 Tat downregulates the expression of Mn-SOD, an effect that potentiates TNF-induced NF-kappa B activation and that requires the C-terminus (amino acids 72-86) of Tat
SOD2	155871	NP_057853.1	Tat	enhances	15223067, 16792821	The membrane transduction efficiencies and biological activities of the SOD protein are enhanced by fusing with the Tat protein transduction domain (PTD) at both termini
SOD2	155871	NP_057853.1	Tat	inhibited by	18160848,	Cell-permeable SOD inhibits the activation of MAP kinases including ERK, JNK and p38 and the upregulation of ICAM-1 and VCAM-1 by HIV-1 Tat
SOD2	155871	NP_057853.1	Tat	inhibited by	18160848,	Treatment of astrocytes with cell-permeable superoxide dismutase (SOD) leads to a decrease in Tat-induced ROS generation and NF-kappaB activation
SOD2	155871	NP_057853.1	Tat	upregulates	24667918,	Microarray analysis indicates HIV-1 Tat-induced upregulation of mitochondrial superoxide dismutase 2 (SOD2; Mn-SOD) in primary human brain microvascular endothelial cells
SOD2	155871	NP_057853.1	Tat	upregulates	26895301,	HIV-1 Tat upregulates NQO1, CAT, SOD1, SOD2, and HMOX1 (HO1) mRNA levels in SH-SY5Y cells
SOD2	155459	NP_057851.1	Vif	upregulates	23333304,	HIV-1 Vif upregulates the expression of superoxide dismutase 2 (SOD2, mitochondrial) in Vif-expression T cells
VWF	155971	NP_579894.2	Envelope surface glycoprotein gp120	downregulates	21612582,	HIV-1 gp120 downregulates vWF expression in human mesenchymal stem cells
VWF	155871	NP_057853.1	Tat	downregulates	22095559,	HIV-1 Tat inhibits the differentiation of mesenchymal stem cells (MSCs) to endothelial cells by downregulating the expression of VEGF-induced endothelial markers such as Flt-1, KDR and vWF
VWF			HIV-1 virus replication	enhanced by expression of human gene	18854154,	Knockdown of von Willebrand factor (VWF) by siRNA inhibits the early stages of HIV-1 replication in 293T cells infected with VSV-G pseudotyped HIV-1
VWF	155971	NP_579894.2	Envelope surface glycoprotein gp120	downregulates	21612582,	HIV-1 gp120 downregulates vWF expression in human mesenchymal stem cells
VWF	155871	NP_057853.1	Tat	downregulates	22095559,	HIV-1 Tat inhibits the differentiation of mesenchymal stem cells (MSCs) to endothelial cells by downregulating the expression of VEGF-induced endothelial markers such as Flt-1, KDR and vWF
VWF			HIV-1 virus replication	enhanced by expression of human gene	18854154,	Knockdown of von Willebrand factor (VWF) by siRNA inhibits the early stages of HIV-1 replication in 293T cells infected with VSV-G pseudotyped HIV-1
GAPDH	155971	NP_579894.2	Envelope surface glycoprotein gp120	complexes with	23125841,	Tandem affinity purification and mass spectrometry analysis identify glyceraldehyde-3-phosphate dehydrogenase (GAPDH), HIV-1 Gag, Gag/Pol, gp120, and Nef incorporated into staufen1 RNP complexes isolated from HIV-1-expressing cells
GAPDH	155030	NP_057850.1	Pr55(Gag)	complexes with	23125841,	Tandem affinity purification and mass spectrometry analysis identify glyceraldehyde-3-phosphate dehydrogenase (GAPDH), HIV-1 Gag, Gag/Pol, gp120, and Nef incorporated into staufen1 RNP complexes isolated from HIV-1-expressing cells
GAPDH	155030	NP_057850.1	Pr55(Gag)	interacts with	23237566,	Immunoprecipitation assay shows that GAPDH directly interacts with HIV-1 Gag and Gag-Pol. Packaging of LysRS and tRNA-Lys3 into virions is negatively regulated by GAPDH, leading to decreased viral infectivity
GAPDH	155348	NP_057849.4	Gag-Pol	complexes with	23125841,	Tandem affinity purification and mass spectrometry analysis identify glyceraldehyde-3-phosphate dehydrogenase (GAPDH), HIV-1 Gag, Gag/Pol, gp120, and Nef incorporated into staufen1 RNP complexes isolated from HIV-1-expressing cells
GAPDH	155348	NP_057849.4	Gag-Pol	interacts with	23237566,	Immunoprecipitation assay shows that GAPDH directly interacts with HIV-1 Gag and Gag-Pol. Packaging of LysRS and tRNA-Lys3 into virions is negatively regulated by GAPDH, leading to decreased viral infectivity
GAPDH	156110	NP_057857.2	Nef	complexes with	23125841,	Tandem affinity purification and mass spectrometry analysis identify glyceraldehyde-3-phosphate dehydrogenase (GAPDH), HIV-1 Gag, Gag/Pol, gp120, and Nef incorporated into staufen1 RNP complexes isolated from HIV-1-expressing cells
GAPDH	155908	NP_057854.1	Rev	interacts with	22174317,	HIV-1 Rev interacting protein, glyceraldehyde-3-phosphate dehydrogenase (GAPDH), is identified by the in-vitro binding experiments involving cytosolic or nuclear extracts from HeLa cells. The interaction of Rev with GAPDH is increased by RRE
GAPDH	155871	NP_057853.1	Tat	interacts with	25496916,	Glyceraldehyde-3-phosphate dehydrogenase (GAPDH) is identified to interact with HIV-1 Tat mutant Nullbasic in HeLa cells by LC MS/MS
GAPDH	155807	NP_057852.2	Vpr	downregulates	23728617,	Treatment of human primary astrocytes with HIV-1 Vpr downregulates expression of mRNA GAPDH and GAPDH activity

GAPDH	155807	NP_057852.2	Vpr	downregulates	23874603,	A stable-isotope labeling by amino acids in cell culture coupled with mass spectrometry-based proteomics identifies downregulation of glyceraldehyde-3-phosphate dehydrogenase (GAPDH) expression by HIV-1 Vpr in Vpr transduced macrophages
GAPDH			HIV-1 virus replication	inhibited by expression of human gene	23237566,	Knockdown of GAPDH by siRNA enhances HIV-1 infectivity in TZM-bl cells
HLA-A	19424028	YP_009028572.1	Asp	binds	25809376,	Two ASP peptide sequences, ASP-YL9 (89YLYNSLLQL97) and ASP-TL10 (79TPNGSIFTTL88), show high binding affinity to HLA-A*02 and HLA-B*07 molecules, respectively
HLA-A	19424028	YP_009028572.1	Asp	inhibited by	20065064, 25701112	Antisense reading frame-derived cryptic epitopes from the gag, pol, and nef genes are inhibited by the predicted HLA-I alleles, and presented by HIV-1-infected CD8+ T-cells
HLA-A	19424028	YP_009028572.1	Asp	inhibited by	25589651,	Antisense reading frame-derived cryptic epitopes from the env gene are inhibited by the HLA-I alleles in CD8+ T-cells
HLA-A	155971	NP_579894.2	Envelope surface glycoprotein gp120	complexes with	2789433, 8671651, 8877415, 9120272, 10546855, 11932387	Conformational changes in HIV-1 gp120, including an enhanced expression of the V3 loop of gp120 and of epitopes that are exposed upon CD4 binding, are consistent with the formation of a multimolecular complex between HLA class I and gp120/160
HLA-A	155971	NP_579894.2	Envelope surface glycoprotein gp120	interacts with	7539755, 9263011, 12427289	Treatment of CD4+ T cells with HIV-1 gp120 significantly increases CD4 association with CD3, CD45RA, CD45RB, CD59, CD38, CD26 and HLA class I, and decreases that with CD45RC
HLA-A	155971	NP_057856.1	Envelope surface glycoprotein gp160, precursor	binds	17116886,	Epitope Env37-46 from HIV-1 gp160 binds strongly to HLA-A3 molecules and forms very stable complexes
HLA-A	155971	NP_057856.1	Envelope surface glycoprotein gp160, precursor	interacts with	1316930, 10799863, 20200278	HIV-1 gp160-derived peptide p18 presented by H-2Dd class I major histocompatibility complex molecules is processed by angiotensin-1 converting enzyme (ACE) prior to T cell stimulation by the peptide p18
HLA-A	155971	NP_579895.1	Envelope transmembrane glycoprotein gp41	upregulates	7913356, 8084338, 9373217	HIV-1 gp41 selectively enhances MHC class I, ICAM-1, IFN-alpha, IFN-beta, and IFN-omega expression in H9 cells
HLA-A	155971	NP_579895.1	Envelope transmembrane glycoprotein gp41	upregulates	8084338,	Soluble HIV-1 gp41 can selectively enhance MHC class I and II expression on human B cells, but does not increase expression of other cell surface antigens such as CD21 and CD54 (ICAM-1)
HLA-A	155971	NP_579895.1	Envelope transmembrane glycoprotein gp41	upregulates	8084338,	Soluble HIV-1 gp41 enhancement effects on MHC class I and II antigen expression can be inhibited by soluble gp41-binding proteins of 45, 49 and 62 kD from human B cells
HLA-A	155030	NP_057850.1	Pr55(Gag)	affects	27120610,	HIV-1 p6 Gag mutation affects HLA-A antigen presentation; p6 mutation of Glu residues to Alas impairs Gag processing & virus release and enhances Gag-membrane association with increased polyubiquitation & entry of Gag into the MHC-1 presentation pathway
HLA-A	155030	NP_057850.1	Pr55(Gag)	binds	17116886,	The HIV-1 Gag 20-28 epitope binds strongly to HLA-A3 molecules and forms very stable complexes
HLA-A	155030	NP_057850.1	Pr55(Gag)	enhances	18097038,	Targeting HIV-1 Gag into the defective ribosomal product pathway enhances MHC class I antigen presentation and CD8+ T cell activation
HLA-A	155030	NP_057850.1	Pr55(Gag)	enhances	25279819,	The S40F mutation in HIV-1 p6 enhances MHC-I antigen presentation of Gag
HLA-A	155030	NP_057850.1	Pr55(Gag)	interacts with	17878955, 24942586, 25165114, 25781986	Protective HLA alleles have a true preference for HIV-1 Gag protein, while non-protective HLA alleles preferentially interact with HIV-1 Nef
HLA-A	155030	NP_057850.1	Pr55(Gag)	interacts with	21482733,	The PTAP L-domains in the p6 domain of HIV-1 Gag regulates ubiquitination of Gag which controls MHC-I presentation and gag processing in the DRiP pathway.
HLA-A	155030	NP_057850.1	Pr55(Gag)	interacts with	22826228,	The degree of HIV-1 Nef-mediated HLA-A2 downregulation strongly influences recognition of virus-infected cells by the Gag-specific CD8+ cytotoxic T lymphocyte clone
HLA-A	155030	NP_057850.1	Pr55(Gag)	upregulates	21778700,	HIV-1 Gag virus-like particles efficiently activate human monocyte-derived dendritic cells (MDDC) and induce MDDC maturation with an associated increase in the surface expression of CD80, CD86 and MHC classes I and II
HLA-A	155030	NP_579880.1	capsid	interacts with	23061377,	HLA supertypes such as HLA B*07, HLA B*58, HLA A*02 and HLA A*03 are most successful in restricting the amino acid positions of epitope dense regions of HIV-1 Nef, CA, and MA with low entropy and hydrophobic property
HLA-A	155030	NP_579876.2	matrix	interacts with	23061377,	HLA supertypes such as HLA B*07, HLA B*58, HLA A*02 and HLA A*03 are most successful in restricting the amino acid positions of epitope dense regions of HIV-1 Nef, CA, and MA with low entropy and hydrophobic property
HLA-A	155030	NP_579883.1	p6	enhances	25279819,	The S40F mutation in HIV-1 p6 enhances MHC-I antigen presentation of Gag
HLA-A	155348	NP_789740.1	Pol	binds	17116886,	The HIV-1 Pol 325-333 epitope binds strongly to HLA-A3 molecules and forms very stable complexes
HLA-A	156110	NP_057857.2	Nef	binds	15569716, 15653685	HIV-1 Nef disrupts antigen presentation by binding to MHC-I (HLA-A2) hypophosphorylated cytoplasmic tails in the endoplasmic reticulum; this Nef-MHC-I complex migrates normally into the Golgi apparatus but subsequently fails to arrive at the cell surface
HLA-A	156110	NP_057857.2	Nef	binds	17116886,	The HIV-1 Nef 73-82 epitope binds strongly to HLA-A3 molecules and forms very stable complexes
HLA-A	156110	NP_057857.2	Nef	binds	9582271, 10366557, 10982373, 11463741, 12414957, 12836198, 14965316, 15078178, 16454711, 18057255, 18073204, 18296443, 18653452, 19149577, 22301137, 22705789, 22767237, 23170180, 23202450	Four glutamic acids from position 62 to 65 in the SH3 domain of HIV-1 Nef bind to the cytoplasmic tail at position 320Y of MHC-I, and are required for the Nef-mediated downregulation of MHC-I from the cell surface
HLA-A	156110	NP_057857.2	Nef	co-localizes with	15854903,	PxxP motifs in HIV-1 Nef induce the accumulation of CCR5 in a perinuclear compartment where both molecules co-localize with MHC-1
HLA-A	156110	NP_057857.2	Nef	complexes with	21917951, 25585010	Dominant active ARF1 (Q71L) potently stabilizes interactions among AP-1 mu1, HIV-1 Nef, and HLA-A2 and that the formation of a static complex sequesters necessary trafficking components
HLA-A	156110	NP_057857.2	Nef	complexes with	22705789, 22767237	Asp327 and Tyr320 of MHC-I, Asp123 of Nef, and Arg225, Arg393, Lys396, Arg211, and Arg246 of mu 1 are involved in a crucial three-way electrostatic network, which results in the Nef-MHC-I CD-mu 1 complex formation
HLA-A	156110	NP_057857.2	Nef	degrades	18725938, 20622010	MHC-I is found in the Rab7(+) vesicles and targeted for degradation via the activity of the Nef-interacting protein, beta-COP
HLA-A	156110	NP_057857.2	Nef	downregulates	10403641, 18155264, 23170180, 23202450	HIV-1 selectively downregulates HLA-A and HLA-B but does not significantly affect HLA-C or HLA-E, which allows HIV-infected cells to avoid NK cell-mediated lysis; this effect is likely mediated by the HIV-1 Nef protein
HLA-A	156110	NP_057857.2	Nef	downregulates	10684310, 15078178, 18073204, 22301137, 22705789, 22767237, 26319395, 26607225	A methionine residue at amino acid 20 in the alpha-helix domain of HIV-1 Nef is required for the ability of Nef to downregulate MHC-I expression but not for the downregulation of CD4
HLA-A	156110	NP_057857.2	Nef	downregulates	10707087, 18005690, 19149577, 20622010	HIV-1 Nef-induced downregulation of MHC-I expression and MHC-I targeting to the trans-Golgi network (TGN) require the binding of Nef to PACS-1, a molecule that controls the TGN localization of the cellular protein furin
HLA-A	156110	NP_057857.2	Nef	downregulates	11289809, 12526811, 18438604, 19149577, 20702582	HIV-1 Nef downregulates expression of MHC-I by blocking transport of MHC-I molecules to the cell surface through a mechanism that requires phosphoinositide 3-kinase (PI 3-kinase) activity
HLA-A	156110	NP_057857.2	Nef	downregulates	11438519,	HIV-1 Nef downregulates MHC-I in Jurkat cells in a concentration-dependent manner
HLA-A	156110	NP_057857.2	Nef	downregulates	11500821,	A dominant-negative mutant protein derived from Hck, (composed of the N-terminal region, SH2, and SH3 domains) interacts with HIV-1 Nef and inhibits Nef-induced downregulation of MHC class I

HLA-A	156110	NP_057857.2	Nef	downregulates	11578695, 16454711	Deletion of the 19 N-terminal amino acids including the myristoylation signal from HIV-1 Nef inhibits both MHC-I and CD4 downregulation while preserving most CTL, T-helper and B-cell epitopes
HLA-A	156110	NP_057857.2	Nef	downregulates	11602047,	Downregulation of major histocompatibility class I on human dendritic cells by HIV-1 Nef impairs antigen presentation to HIV-specific CD8+ T lymphocytes
HLA-A	156110	NP_057857.2	Nef	downregulates	12097566, 15611225	HIV-1 Nef-mediated downregulation of HLA class I suppresses the cytolytic activity of HIV-1-specific cytotoxic T-lymphocyte (CTL) clones
HLA-A	156110	NP_057857.2	Nef	downregulates	12482663, 12884192	HIV-1 Nef downregulates human MHC-I more efficiently than murine MHC-I molecules in HeLa cells, and Nef does not function efficiently in murine endothelial cells
HLA-A	156110	NP_057857.2	Nef	downregulates	14557639, 16354571, 16454711, 16684552	HIV-1 Nef alleles derived from perinatally infected children efficiently downregulate both CD4 and MHC-I in HeLa-CD4+ cells
HLA-A	156110	NP_057857.2	Nef	downregulates	15194762,	HIV-1 group N and group O Nef alleles only weakly downregulate CD4, CD28, and class I and II MHC molecules
HLA-A	156110	NP_057857.2	Nef	downregulates	15262497,	HIV-1 Nef has been observed to downregulate HLA-A2 on immature dendritic cells from two donors
HLA-A	156110	NP_057857.2	Nef	downregulates	15878340,	The HIV-1 Nef mutant NefAAAA, which cannot interact with the endosomal sorting protein PACS-1, increases the number of cells containing long and stable tubules, which allows the internalization of MHC-1 into the tubules from the cell surface
HLA-A	156110	NP_057857.2	Nef	downregulates	16000390, 16091223	Macrophage-tropic HIV-1 Nef downregulates expression of HLA-A2 on the surface of productively infected macrophages; point mutations in Nef at prolines P74 or P80 abrogate the downregulation of HLA-A2
HLA-A	156110	NP_057857.2	Nef	downregulates	16365153,	HIV-1 Nef induces drastic and moderate downregulation of CD4 and MHC-I in resting CD4(+) T lymphocytes, respectively, but markedly upregulates cell surface levels of the MHC-II invariant chain CD74
HLA-A	156110	NP_057857.2	Nef	downregulates	16847125,	Mutation of amino acid P78 in HIV-1 Nef affects downregulation of MHC-I molecules from the cell surface, but does not interfere with Nef binding to Src homology 3 (SH3) domains
HLA-A	156110	NP_057857.2	Nef	downregulates	17581864, 20622010, 25585010	Knocking down either AP-1 gamma, AP-1 mu1, or clathrin strongly inhibits Nef-induced downregulation of HLA-A2
HLA-A	156110	NP_057857.2	Nef	downregulates	18005690, 18296443, 25585010	Nef/Hck complex recruits and phosphorylates the tyrosine kinase ZAP-70, which binds class I PI3K to trigger MHC-I downregulation in primary CD4+ T cells
HLA-A	156110	NP_057857.2	Nef	downregulates	18005690, 25585010	In promonocytic cells, Nef/Hck recruits the ZAP-70 homolog Syk to downregulate MHC-I
HLA-A	156110	NP_057857.2	Nef	downregulates	18073204,	Mutating three amino acids (Y320, A324, and D327) in the cytoplasmic tail of HLA-A2 abrogates Nef-induced downregulation of HLA-A2 through a failuer to recruit the mu1 or gamma subunits of AP-1
HLA-A	156110	NP_057857.2	Nef	downregulates	18296443, 25585010	HIV-1 Nef-mediated downregulation of MHC-I requires Nef motif EEEE(65)-dependent binding to the sorting protein PACS-2, which targets Nef to the paranuclear region and enables Nef PXXP(75) to bind and activate a trans-Golgi network localized Src kinase
HLA-A	156110	NP_057857.2	Nef	downregulates	20622010, 21917951	ARF6(T27N/Q67L) and RAB11(Q67L) mutants induce significant reversal of HLA-I A2 downregulation by HIV-1 Nef through redistributing HLA-I A2 from the perinuclear vesicles to the peripheral punctate vesicles at the plasma membrane
HLA-A	156110	NP_057857.2	Nef	downregulates	21543478, 23170180, 23202450, 25585010	beta-COP as a cellular cofactor is required for HIV-1 Nef-mediated HLA-A2, CD4, and CD8 downregulation
HLA-A	156110	NP_057857.2	Nef	downregulates	22301137,	Double (W13A/V16R) and triple (W13A/V16R/M20A) substitution mutants of HIV-1 Nef fail to downregulate MHC-I
HLA-A	156110	NP_057857.2	Nef	downregulates	22537596,	The HIV-1 Nef highly conserved valine-glycine-phenylalanine amino acid triplet (VGF) motif, which links the acidic cluster and the proline-rich motif, is important for downregulation of CXCR4 and MHC-I
HLA-A	156110	NP_057857.2	Nef	downregulates	22553319,	HIV-1 Nef with A84D, Y135F, and G140R mutation impairs to its ability to downregulate MHC-I
HLA-A	156110	NP_057857.2	Nef	downregulates	22826228,	HLA-A2 molecules with HLA-A cytoplasmic domains are more downregulated by HIV-1 Nef than those with HLA-B domains. There is no downregulation of HLA-A2 with HLA-C cytoplasmic domains by Nef
HLA-A	156110	NP_057857.2	Nef	downregulates	23289738,	HIV-1 Nef clones, isolated from plasma of elite controllers (EC) and chronic progressors (CP), show significantly lower HLA class I downregulation activity in EC than that in CP
HLA-A	156110	NP_057857.2	Nef	downregulates	24041011, 25193656	HIV-1 Nef clones obtained from chronic patients infected with HIV-1 subtypes A, B, C or D show a functional hierarchy of subtype B > A/D > C for Nef-mediated HLA class I downregulation
HLA-A	156110	NP_057857.2	Nef	downregulates	24965469,	HIV-1 Nef clones from acute controllers display a lesser ability to downregulate CD4 and HLA class I from the cell surface, and a reduced ability to enhance virion infectivity compared to those from acute progressors
HLA-A	156110	NP_057857.2	Nef	downregulates	26439863,	HIV-1 Nef downregulates cell (CEMT4) surface expression of HLA-A
HLA-A	156110	NP_057857.2	Nef	downregulates	26607225,	HIV-1 NL4-3 and SK68 Nef downregulates HLA-A (HLA-A*02), which is dependent upon amino acids M20 and S88
HLA-A	156110	NP_057857.2	Nef	downregulates	26656785,	HIV-1 (SF2) Nef downregulates MHC-I (HLA-A/B/C); downregulation is dependent upon a proline-rich SH3 binding domain in Nef
HLA-A	156110	NP_057857.2	Nef	downregulates	26700863,	HIV-1 NL4-3 Nef downregulates HLA-A/B/C, which moderately requires the CPG-motif in Nef
HLA-A	156110	NP_057857.2	Nef	downregulates	26787826,	HIV-1 NL4-3 and subtype B Nef downregulates HLA-A more than HLA-B, which discerned by amino acid 202 in Nef

HLA-A	156110	NP_057857.2	Nef	downregulates	8612235, 12734410, 15638726, 16091223, 16272310, 16979207, 16987968, 17077296, 17581864, 17586321, 17632197, 17632570, 18005680, 18005690, 18073204, 18155264, 18296443, 18438604, 18473783, 18541215, 18653452, 18725938, 18808677, 19091857, 19149577, 19449444, 19555986, 19643141, 19770068, 20012528, 20380698, 20594957, 20702582, 21068258, 21165790, 21209113, 21482738, 21543478, 21762823, 21849975, 21861776, 21917951, 21922073, 21994772, 22103831, 22103833, 22103834, 22175768, 22301137, 22301152, 22537596, 22553319, 22613796, 22651890, 22826228, 22844345, 22980333, 23170180, 23202450, 23289738, 23490051, 23847689, 23853598, 23986795, 24023945, 24041011, 24058696, 24158818, 24172637, 24192765, 24400003, 24495362, 24748005, 24789790, 24904546, 24965469, 25193656, 25275127, 25423108, 25525794, 25827531,	HIV-1 Nef downregulates the expression of MHC-I at the surface of lymphoid, monocytic and epithelial cells, causing MHC-I molecules to be rapidly internalized, accumulated in endosomal vesicles and degraded
HLA-A	156110	NP_057857.2	Nef	downregulates	9052838, 9586638, 18438604, 18808677, 19149577, 19847956, 20622010, 22705789, 22767237, 23170180, 23202450	Interaction of HIV-1 Nef with the mu subunit of AP adaptor complexes requires the recognition of tyrosine-based sorting signals, which likely facilitates the connection between MHC I and the clathrin-dependent sorting machinery during MHC I downregulation
HLA-A	156110	NP_057857.2	Nef	downregulates	9123874,	Downregulation of MHC-I by HIV-1 Nef decreases the incorporation of MHC-I molecules into virions, but does not decrease virion infectivity
HLA-A	156110	NP_057857.2	Nef	inhibits	11289809, 12584329, 20622010, 23170180, 23202450, 23678182, 25915798	Expression of HIV-1 Nef in human T cells inhibits HLA-A2 transport to the cell surface
HLA-A	156110	NP_057857.2	Nef	inhibits	15569716, 20622010, 23678182	The ability of HIV-1 Nef to disrupt MHC-I trafficking and inhibit antigen presentation is regulated by the expression of the mu1 subunit of adaptor protein (AP) AP-1A, a cellular protein complex implicated in TGN to endolysosomal pathways
HLA-A	156110	NP_057857.2	Nef	inhibits	15596859, 23170180, 23202450	The N-terminal alpha helix (17-26), polyproline (72-78), acidic (62-65), and oligomerization (123) domains of HIV-1 Nef are required for Nef-mediated disruption of the transport of HLA-A2 to the cell surface and for Nef to coprecipitate with HLA-A2
HLA-A	156110	NP_057857.2	Nef	interacts with	17581864,	Knocking down AP-2 enhances Nef activity by causing increased delivery of HLA-A2 to a prelysosomal compartment
HLA-A	156110	NP_057857.2	Nef	interacts with	17878955, 25165114	Protective HLA alleles have a true preference for HIV-1 Gag protein, while non-protective HLA alleles preferentially interact with HIV-1 Nef
HLA-A	156110	NP_057857.2	Nef	interacts with	18057255, 18073204	HIV-1 Nef acidic (Glu62-65) and polyproline domains (Pro75/78) stabilize the interaction between the HLA-A2/Nef fusion protein and AP-1 mu1
HLA-A	156110	NP_057857.2	Nef	interacts with	23061377, 24886641	HLA supertypes such as HLA B*07, HLA B*58, HLA A*02 and HLA A*03 are most successful in restricting the amino acid positions of epitope dense regions of HIV-1 Nef, CA, and MA with low entropy and hydrophobic property
HLA-A	156110	NP_057857.2	Nef	interacts with	26061722,	HIV-1 Nef interacts with HLA-A (MHC1) and this interaction occurs partially within RAB5+ early endosomes
HLA-A	156110	NP_057857.2	Nef	modulates	11396948, 14557639, 22613796, 23490051	Different levels of MHC-I modulation are induced by different HIV-1 Nef proteins derived from HIV-1 infected adults and children
HLA-A	156110	NP_057857.2	Nef	modulates	9971776, 12502873, 15078178, 17170457, 18005690, 18073204	Two distinct regions of HIV-1 Nef modulate MHC-I cell surface expression: an N-terminal alpha-helix (residues 17-26) and a proline-rich motif (residues 75-78)
HLA-A	156110	NP_057857.2	Nef	relocalizes	20622010,	HIV-1 Nef sequesters HLA-I A2 and colocalizes with CD63 and LAMP1 markers in late endosomes and lysosomes
HLA-A	155871	NP_057853.1	Tat	downregulates	17604883,	Four mutations (C27S, K51T, R55L, and G79A) on HIV-1 Tat result in the loss of the deleterious effects of Tat on the expression of MHC I, IL-2, and CD25 genes compared with wild-type Tat in Jurkat cells
HLA-A	155871	NP_057853.1	Tat	downregulates	7621073, 8493575, 9751712, 9840288	HIV-1 Tat represses the MHC class I gene promoter by binding to and repressing TAFII250, a component of the general transcription factor TFIID, suggesting a mechanism for HIV-1 to downregulate MHC class I expression and avoid immune surveillance
HLA-A	155871	NP_057853.1	Tat	upregulates	11751963, 14595379	HIV-1 Tat upregulates MHC class I in monocyte-derived dendritic cells and CD8(+) T cells, thereby driving T cell-mediated immune responses
HLA-A	155807	NP_057852.2	Vpr	upregulates	23874603,	A stable-isotope labeling by amino acids in cell culture coupled with mass spectrometry-based proteomics identifies upregulation of HLA-A (A-68 alpha chain) expression by HIV-1 Vpr in Vpr transduced macrophages
HLA-A	155945	NP_057855.1	Vpu	downregulates	9104816, 18672082, 20012522, 24400003, 25275127, 25620704	Using antibodies specific to MHC class I A, B, and C molecules (clone W6/32), HIV-1 Vpu protein has been shown to downregulate the expression of MHC class I molecules on the surface of HIV-1 infected cells
HLA-A	155945	NP_057855.1	Vpu	regulated by	22503975,	HLA class I-associated immune responses have minor effects on Vpu variability, suggesting that Vpu conformation and function are preserved through many possible combinations of primary and secondary polymorphisms
HLA-A			HIV-1 virus replication	downregulates expression of human gene	26439863,	HIV-1 infection (VSV-G pseudotyped) of CEMT4 T cells downregulates plasma membrane expression of HLA-A
HLA-A			HIV-1 virus replication	downregulates expression of human gene	26497177,	Capsid expressing (p24+) cells from pleural fluid of HIV-1/TB coinfected patients or in vitro infected PBMC (NL4-3 or NLAD8) downregulate HLA-A/B/C and BST2 (Tetherin) concomitantly with CD4 downregulation
ITGB1	155971	NP_579894.2	Envelope surface glycoprotein gp120	inhibits	22241990,	SLIT2 inhibits HIV-1 gp120-induced lymphatic hyperpermeability by blocking the interaction between Robo4 and integrin alpha5beta1 in human lymphatic endothelial cells

ITGB1	155971	NP_579894.2	Envelope surface glycoprotein gp120	interacts with	23152803,	HIV-1 Tat complexes with gp120 to induce entry of VLPs expressing R5- or X4-tropic Env into MDDCs, which involves alpha5beta1, alpha5beta3, and alpha5beta5 integrins
ITGB1	155971	NP_057856.1	Envelope surface glycoprotein gp160, precursor	interacts with	27375898,	HIV-1 gp160 interacts with ITGB1; predicted interaction to be relevant to viral egress at plasma membrane/extracellular matrix
ITGB1	156110	NP_057857.2	Nef	interacts with	23284715,	The yeast two-hybrid screen and the coimmunoprecipitation analysis identify the HIV-1 Nef interacting human protein integrin, beta 1 (ITGB1) in cells
ITGB1	155871	NP_057853.1	Tat	activates	22423313,	The KKR spatial domain (Lys12, Lys41, and Arg78) in HIV-1 Tat contributes to Tat-mediated cell adhesion via integrin beta 1 activation in a cell surface HSPG-dependent manner
ITGB1	155871	NP_057853.1	Tat	binds	2202737, 7539135, 7690138, 9517988, 10397733, 21951552	The arginine-glycine-aspartic acid (RGD) sequence present at the carboxy-terminal of HIV-1 Tat mediates vascular cell and monocyte migration and invasion by binding to the alpha-5-beta-1 and alpha-v-beta-3 integrins
ITGB1	155871	NP_057853.1	Tat	cooperates with	20661303,	HIV-1 Tat-mediated inhibition of autophagy in bystander macrophages/monocytic cells requires CXCR4, VEGFR1, and beta-integrins
ITGB1	155871	NP_057853.1	Tat	interacts with	17868650,	Sulfated polymannuroguluronate, a novel anti-AIDS drug candidate, greatly arrests Tat-driven KDR phosphorylation and blocks the interaction between Tat and integrin beta1, thus inhibiting the phosphorylation of the kinases FAK, paxillin and MAPKs
ITGB1	155871	NP_057853.1	Tat	interacts with	23152803,	HIV-1 Tat complexes with gp120 to induce entry of VLPs expressing R5- or X4-tropic Env into MDDCs, which involves alpha5beta1, alpha5beta3, and alpha5beta5 integrins
ITGB1	155871	NP_057853.1	Tat	interacts with	24742657,	Treatment with cannabinoids inhibits HIV-1 Tat-enhanced attachment of U937 cells to collagen IV, laminin, or ECM1 proteins, which is linked to the cannabinoid receptor type 2 and the modulation of beta1-integrin and actin distribution
ITGB1	155871	NP_057853.1	Tat	interacts with	25313583,	HIV-1 Tat-induced inhibition of IFN-gamma release is regulated by the interaction of Tat-RGD domain with alpha5beta1 and alpha5beta3 integrins in CD8+ T cells
ITGB1	155871	NP_057853.1	Tat	interacts with	7539135, 7690138, 10397733, 17868650	HIV-1 Tat induces angiogenesis and cooperates in the development of AIDS-associated Kaposi sarcoma as a result of interactions with integrins alpha-5-beta-1 and alpha-v-beta 3
ITGB1	155871	NP_057853.1	Tat	interacts with	8757349,	Interaction of HIV-1 Tat with alpha 5, beta 1, and alpha v subunits of surface integrin receptors mediates activation of CD4+ T cells
ITGB1	155871	NP_057853.1	Tat	interacts with	8757599,	HIV-1 Tat induced monocyte invasion is inhibited by anti-beta integrin Ab or tissue inhibitor of metalloproteinase (TIMP), indicating an interaction with beta integrins and TIMP
ITGB1	155871	NP_057853.1	Tat	interacts with	9916748,	IFN-gamma interacts with HIV-1 Tat to induce endothelial cells to proliferate and invade the extracellular matrix by upregulating the receptors for Tat (integrins alpha-5-beta-1 and alpha-v-beta-3), suggesting Tat and IFN-gamma play major roles in AIDS-KS
ITGB1	155807	NP_057852.2	Vpr	upregulates	23874603,	A stable-isotope labeling by amino acids in cell culture coupled with mass spectrometry-based proteomics identifies upregulation of integrin, beta 1 (ITGB1, fibronectin receptor, CD29) expression by HIV-1 Vpr in Vpr transduced macrophages
S100A9			HIV-1 virus replication	decreases expression of human gene	26220577,	Clinical HIV-1 infection decreases S100A9 in blood plasma of patients with symptomatic neurocognitive disorders
ENO1	155971	NP_579894.2	Envelope surface glycoprotein gp120	complexes with	23125841,	Tandem affinity purification and mass spectrometry analysis identify alpha unit of enolase 1 (ENO1), HIV-1 Gag, Gag/Pol, gp120, and Nef incorporated into staufen1 RNP complexes isolated from HIV-1-expressing cells
ENO1	155030	NP_057850.1	Pr55(Gag)	complexes with	23125841,	Tandem affinity purification and mass spectrometry analysis identify alpha unit of enolase 1 (ENO1), HIV-1 Gag, Gag/Pol, gp120, and Nef incorporated into staufen1 RNP complexes isolated from HIV-1-expressing cells
ENO1	155348	NP_057849.4	Gag-Pol	complexes with	23125841,	Tandem affinity purification and mass spectrometry analysis identify alpha unit of enolase 1 (ENO1), HIV-1 Gag, Gag/Pol, gp120, and Nef incorporated into staufen1 RNP complexes isolated from HIV-1-expressing cells
ENO1	155348	NP_705926.1	retropepsin	cleaves	22944692,	Positional proteomics analysis identifies the cleavage of human enolase 1, alpha (ENO1) at amino acid residues 115-116 by the HIV-1 protease
ENO1	156110	NP_057857.2	Nef	complexes with	23125841,	Tandem affinity purification and mass spectrometry analysis identify alpha unit of enolase 1 (ENO1), HIV-1 Gag, Gag/Pol, gp120, and Nef incorporated into staufen1 RNP complexes isolated from HIV-1-expressing cells
ENO1	156110	NP_057857.2	Nef	downregulates	25874870,	HIV-1 Nef downregulates the expression of enolase 1 (ENO1) protein in Nef-transfected SupT1 cells
ENO1	155871	NP_057853.1	Tat	inhibits	9093905,	MBP-1 suppresses transcription from the HIV-1 LTR promoter, an effect that is inhibited by HIV-1 Tat, suggesting an important role for MBP-1 in the regulation of HIV-1 replication in infected cells
P4HB	155971	NP_579894.2	Envelope surface glycoprotein gp120	cleaved by	12218051, 12218052, 23206338	Protein-disulfide isomerase (PDI) cleaves disulfide bonds in recombinant HIV-1 envelope glycoprotein gp120, and gp120 bound to the surface receptor CD4 undergoes a disulfide reduction that is prevented by PDI inhibitors
P4HB	155971	NP_579894.2	Envelope surface glycoprotein gp120	interacts with	20458450,	The disulfide cross-linking interaction between gp120 and PDI is enhanced by CD4 protein
P4HB	155971	NP_579894.2	Envelope surface glycoprotein gp120	interacts with	23206338,	PDI is predominantly involved in HIV-1 entry and infection of the T cell line PM-1 and PHA-stimulated primary T lymphocytes, suggesting the preferential use of PDI relevant to the HIV-1 entry and establishment of virus reservoirs in resting CD4+ cells
P4HB	155971	NP_057856.1	Envelope surface glycoprotein gp160, precursor	interacts with	22190034,	HIV-1 gp160 is identified to have a physical interaction with prolyl 4-hydroxylase, beta polypeptide (P4HB) in human HEK293 and/or Jurkat cell lines by using affinity tagging and purification mass spectrometry analyses
P4HB	155971	NP_057856.1	Envelope surface glycoprotein gp160, precursor	processed by	17301129,	Treatment of trimeric HIV-1 rgp140 with protein disulfide isomerase yields monomers by disruption of the intermolecular disulfide bonds
P4HB	155908	NP_057854.1	Rev	interacts with	22174317,	HIV-1 Rev interacting protein, prolyl 4-hydroxylase, beta polypeptide (P4HB), is identified by the in-vitro binding experiments involving cytosolic or nuclear extracts from HeLa cells. The interaction of Rev with P4HB is increased by RRE
P4HB	155807	NP_057852.2	Vpr	upregulates	23874603,	A stable-isotope labeling by amino acids in cell culture coupled with mass spectrometry-based proteomics identifies upregulation of prolyl 4-hydroxylase, beta polypeptide (P4HB, PDIA1) expression by HIV-1 Vpr in Vpr transduced macrophages
PRSS1	155971	NP_579894.2	Envelope surface glycoprotein gp120	cleaved by	1763044,	Cleavage of HIV-1 gp120 with trypsin at residue 432 destroys CD4 binding
PFN1	155807	NP_057852.2	Vpr	downregulates	23874603,	A stable-isotope labeling by amino acids in cell culture coupled with mass spectrometry-based proteomics identifies downregulation of profilin 1 (PFN1) expression by HIV-1 Vpr in Vpr transduced macrophages
THBS1	155971	NP_579894.2	Envelope surface glycoprotein gp120	binds	9419208,	Interaction of TSP1 with HIV-1 gp120 involves CSVTCG sequences in the type 1 properdin-like repeats of TSP1 and amino acids 281-300, 311-330, and 361-380 in the C2-V3-C3 domains of gp120
THBS1	155971	NP_579894.2	Envelope surface glycoprotein gp120	binds	9419208,	TSP1 inhibits HIV-1 infection of peripheral blood mononuclear cells and transformed T and promonocytic cell lines by its binding to HIV-1 gp120
THBS1	155871	NP_057853.1	Tat	binds	11023976,	Thrombospondin-1 (TSP) binds to HIV-1 Tat, an interaction that can be inhibited by heparin which can bind to both TSP and Tat
THBS1	155871	NP_057853.1	Tat	inhibited by	10398144,	Thrombospondin-1 (TSP) prevents endothelial cell motility induced by HIV-1 Tat and inhibits angiogenic activity exerted by Tat in the Matrigel sponge model, suggesting downregulation of TSP may be permissive for development of KS-associated angiogenesis
THBS1	155871	NP_057853.1	Tat	inhibited by	11023976,	Thrombospondin-1 inhibits cell internalization and HIV-1 LTR transactivating activity of extracellular HIV-1 Tat, cell interaction and mitogenic activity of extracellular Tat, as well as the autocrine loop of stimulation exerted by endogenous Tat
THBS1	155807	NP_057852.2	Vpr	stimulates	12444143,	HIV-1 Vpr potentiates the stimulation of thrombospondin 1 by glucocorticoids via the glucocorticoid receptor pathway
MMP2	155971	NP_579894.2	Envelope surface glycoprotein gp120	activates	17678975, 22591362	Matrix metalloproteinases MMP2 and MMP9 participate in the maturation process of cleavage and activation of IL-1beta based on the intracerebroventricular injection of the HIV-1 envelope glycoprotein gp120
MMP2	155971	NP_579894.2	Envelope surface glycoprotein gp120	upregulates	15955449, 22448134, 27605665	HIV-1 gp120 interaction with human mannose receptor (hMR) results in increased production of matrix metalloproteinase-2 (MMP-2) in astrocytes

MMP2	155971	NP_579894.2	Envelope surface glycoprotein gp120	upregulates	27605665,	HIV-1 JRFL Env (gp120) upregulates MMP2 in ARPE-19 and primary human RPE cells and is dependent upon DC-SIGN
MMP2	155971	NP_579895.1	Envelope transmembrane glycoprotein gp41	activates	9699154,	Treatment of human glial and neuronal cells with an HIV-1 gp41 peptide (amino acids 583-599) markedly increases the activity of matrix metalloproteinase 2 (MMP-2)
MMP2	156110	NP_057857.2	Nef	activates	19455469,	Overexpression of HIV-1 Nef and ALK effect gelatinase activation in astrocytic glioma cells
MMP2	155871	NP_057853.1	Tat	induces release of	14982725,	HIV-1 Tat and methamphetamine enhance the release of MMP-1, MMP-2, and uPA from human brain cells
MMP2	155871	NP_057853.1	Tat	upregulates	11220743, 22591362	HIV-1 Tat from AIDS patients with HIV-associated dementia upregulates MMP-2 and MMP-7 release and activation, leading to neurotoxicity
MMP2	155871	NP_057853.1	Tat	upregulates	7935812, 11598182	HIV-1 Tat and bFGF synergize to upregulate MMP-2 (type-IV collagenase) secretion and activation in endothelial cells and also synergize in inducing angiogenic Kaposi's sarcoma-like lesions in mice as a result of enhanced MMP-2 expression
MMP2	155945	NP_057855.1	Vpu	upregulates	24551192,	HIV-1 Vpu-expressing U937 monocytes coculture with LX2 stellate cells to upregulate expression of profibrogenic markers COL-1, PCT, SMA-1, VEGF, and MMP2, which is inhibited by MIF treatment
CD14	156110	NP_057857.2	Nef	downregulates	22808111,	CD14 is significantly downregulated from the surface of HIV-1 Nef-expressing THP-1 monocytes compared to that from the surface of control cells
CD14	156110	NP_057857.2	Nef	upregulates	12414752,	HIV-1 Nef enhances membrane-bound (m) CD14 expression on monocytes but does not induce the release of soluble CD14 into the culture supernatants of PBMC; the upregulation of mCD14 expression does not involve endogenously produced IL-10
FCGR3A	155971	NP_579894.2	Envelope surface glycoprotein gp120	binds	21832933,	IgG2 and IgG4 bind more poorly to enzymatically deglycosylated recombinant gp120 (rgp120) than to unchanged rgp120
FCGR3A	155971	NP_579894.2	Envelope surface glycoprotein gp120	downregulates	22496218,	NK cells that respond with IFN-gamma and TNF-alpha cytokine production to HIV-1 gp120 peptides have reduced CD16 and NKp46 expression and have increased levels of CD57
FCGR3A	156110	NP_057857.2	Nef	antagonizes	25980612,	Production of infectious triple deletion vpr/vpu/nef HIV-1 mutant is suppressed, indicating that Nef may antagonize restriction activity of FCGR3A against production of infectious wild-type HIV-1
FCGR3A	155871	NP_057853.1	Tat	inhibits	9743356,	HIV-1 Tat inhibits the rise in intracellular free calcium concentration in Natural Killer (NK) cells upon cross-linking of the adhesion molecule CD11a and the activation molecule CD16, indicating Tat is involved in the impairment of NK cell function
FCGR3A	155871	NP_057853.1	Tat	upregulates	25250834,	HIV-1 Tat upregulates the expression of CCR2, CD16, and TLR4 in monocyte-derived macrophages
FCGR3A	155807	NP_057852.2	Vpr	antagonizes	25980612,	Production of infectious triple deletion vpr/vpu/nef HIV-1 mutant is suppressed, indicating that Vpu may antagonize restriction activity of FCGR3A against production of infectious wild-type HIV-1
FCGR3A	155945	NP_057855.1	Vpu	antagonizes	25980612,	Production of infectious triple deletion vpr/vpu/nef HIV-1 mutant is suppressed, indicating that Vpu may antagonize restriction activity of FCGR3A against production of infectious wild-type HIV-1
FCGR3A	155945	NP_057855.1	Vpu	interacts with	24623433, 25396265	The ability of HIV-1 Vpu to antagonize tetherin is important for the antibody opsonization of HIV-infected cells, which in turn increases FCGRIII (CD16) signaling
FCGR3A			HIV-1 virus replication	affected by expression of human gene	26613093,	HIV-1 is vertically transmitted more readily by mothers who are heterozygous for high-affinity and low-affinity FCGR3A
GSTP1	155807	NP_057852.2	Vpr	upregulates	23874603,	A stable-isotope labeling by amino acids in cell culture coupled with mass spectrometry-based proteomics identifies upregulation of glutathione S-transferase pi 1 (GSTP1) expression by HIV-1 Vpr in Vpr transduced macrophages
C4B	155971	NP_579894.2	Envelope surface glycoprotein gp120	binds	7590866,	Amino acid residues 410-449 of HIV-1 gp120 are involved in its binding to C4b
C4B	155971	NP_579894.2	Envelope surface glycoprotein gp120	binds	7642209, 7893437, 7911492	Complement proteins C4, C3d, C5b-9, and properdin bind to HIV-1 gp120-coated CD4+ T cells of healthy individuals when incubated in autologous serum
C4B	155971	NP_579894.2	Envelope surface glycoprotein gp120	binds	8630395,	A synthetic peptide covering positions 233-251 of the HIV-1 gp120 protein binds to complement proteins C3, C4, C5, C9, and properdin
C4B	155971	NP_579894.2	Envelope surface glycoprotein gp120	interacts with	7893437,	Preincubation of HIV-1 gp41 with either factor H or properdin, and of HIV-1 gp120 with C3b or C4b affect the interaction between HIV-1 gp41 and gp120
SAA1	155971	NP_579894.2	Envelope surface glycoprotein gp120	upregulates	23867815,	HIV-1 gp120-treated vaginal epithelial cells show upregulation of serum amyloid A1 (SAA1) expression as compared to untreated control
PTPRF			HIV-1 virus replication	downregulates expression of human gene	26439863,	HIV-1 infection (VSV-G pseudotyped) of CEMT4 T cells downregulates plasma membrane expression of PTPRF
HSPA5	155971	NP_579894.2	Envelope surface glycoprotein gp120	complexes with	23125841,	Tandem affinity purification and mass spectrometry analysis identify heat shock 70kDa protein 5 (HSPA5; 78kDa glucose-regulated protein), HIV-1 Gag, Gag/Pol, gp120, and Nef incorporated into staufen1 RNP complexes isolated from HIV-1-expressing cells
HSPA5	155971	NP_579894.2	Envelope surface glycoprotein gp120	inhibited by	12832005,	Over expression of hsp70 with a herpes viral amplicon vector protects cultured hippocampal rat neurons from gp120 neurotoxicity
HSPA5	155971	NP_579894.2	Envelope surface glycoprotein gp120	upregulates	26740125,	HIV-1 Env gp120 upregulates HSPA5 (GRP78/BiP) in SVGA cells and human fetal astrocytes
HSPA5	155971	NP_579894.2	Envelope surface glycoprotein gp120	upregulates	7906708,	The exposure of permissive CD4+ cells to HIV-1 gp120 increases the synthesis and nuclear translocation of 70kDa heat shock protein
HSPA5	155971	NP_057856.1	Envelope surface glycoprotein gp160, precursor	interacts with	1900540, 10514465	Newly synthesized HIV-1 gp160 interacts with GRP78-BiP in pulse-chase experiments; the interaction sites of gp160 with BiP include residues 115-132, 484-490, 602-616, 676-690, and 776-807
HSPA5	155971	NP_057856.1	Envelope surface glycoprotein gp160, precursor	interacts with	27375898,	HIV-1 gp160 interacts with HSPA5; predicted interaction to be within the endoplasmic reticulum and function as chaperone for endoplasmic reticulum-associated degradation
HSPA5	155030	NP_057850.1	Pr55(Gag)	complexes with	23125841,	Tandem affinity purification and mass spectrometry analysis identify heat shock 70kDa protein 5 (HSPA5; 78kDa glucose-regulated protein), HIV-1 Gag, Gag/Pol, gp120, and Nef incorporated into staufen1 RNP complexes isolated from HIV-1-expressing cells
HSPA5	155030	NP_057850.1	Pr55(Gag)	incorporates	11932435,	Hsp70 co-sediments with HIV-1 capsid protein in sucrose density gradients, providing evidence that it is specifically incorporated into HIV-1 virions through an interaction with HIV-1 Gag proteins
HSPA5	155030	NP_057850.1	Pr55(Gag)	incorporates	11932435, 21738476	Hsp70 is incorporated into HIV-1 virions through an interaction with HIV-1 Gag
HSPA5	155030	NP_579876.2	matrix	stimulated by	10964507,	Hsp70 facilitates nuclear import of HIV-1 preintegration complexes by stimulating the binding of HIV-1 Matrix to karyopherin alpha
HSPA5	155348	NP_057849.4	Gag-Pol	complexes with	23125841,	Tandem affinity purification and mass spectrometry analysis identify heat shock 70kDa protein 5 (HSPA5; 78kDa glucose-regulated protein), HIV-1 Gag, Gag/Pol, gp120, and Nef incorporated into staufen1 RNP complexes isolated from HIV-1-expressing cells
HSPA5	156110	NP_057857.2	Nef	complexes with	23125841,	Tandem affinity purification and mass spectrometry analysis identify heat shock 70kDa protein 5 (HSPA5; 78kDa glucose-regulated protein), HIV-1 Gag, Gag/Pol, gp120, and Nef incorporated into staufen1 RNP complexes isolated from HIV-1-expressing cells
HSPA5	156110	NP_057857.2	Nef	interacts with	21763498,	Heat shock proteins Hsp40 and Hsp70 interact with HIV-1 Nef and form a complex in cells
HSPA5	155871	NP_057853.1	Tat	activates	20457808,	Exposure of human umbilical vein endothelial cells to HIV-1 Tat causes broad activation of the unfolded-protein response in ER with phosphorylation of PERK, eIF2alpha, and JNK and induction of Grp78/BiP
HSPA5	155871	NP_057853.1	Tat	regulated by	10617616,	Hsp70 and Hsp90 and Cdc37 regulate the stabilization and folding of CDK9 as well as the assembly of an active CDK9/cyclin T1 complex responsible for P-TEFb-mediated HIV-1 Tat transactivation
HSPA5	155807	NP_057852.2	Vpr	competes with	10964507, 19275587	HIV-1 Vpr competes with Hsp70 for binding to karyopherin alpha
HSPA5	155807	NP_057852.2	Vpr	interacts with	21763498,	HIV-1 Vpr is required for the inhibitory effect of Hsp70 on viral gene expression and replication
HSPA5	155807	NP_057852.2	Vpr	upregulates	22438978,	HIV-1 Vpr significantly increases expression level of GRP78 in the endoplasmic reticulum
HSPA5	155807	NP_057852.2	Vpr	upregulates	23874603,	A stable-isotope labeling by amino acids in cell culture coupled with mass spectrometry-based proteomics identifies upregulation of heat shock 70kDa protein 5 (HSPA5, GRP78) expression by HIV-1 Vpr in Vpr transduced macrophages

CDH1	155971	NP_579894.2	Envelope surface glycoprotein gp120	inhibits	24586397,	HIV-1 gp120 inhibits the expression of E-cadherin and changes its localization from cell membrane to cytoplasm in polarized oral epithelial cells
CDH1	155871	NP_057853.1	Tat	induces phosphorylation of	24965120,	HIV-1 Tat C induces phosphorylation of adherens junction proteins VE-cadherin and beta-catenin in human brain microvascular endothelial cells
CDH1	155871	NP_057853.1	Tat	inhibits	24586397,	HIV-1 Tat inhibits the expression of E-cadherin and changes its localization from cell membrane to cytoplasm in polarized oral epithelial cells
CDH1	155871	NP_057853.1	Tat	regulates	24965120,	HIV-1 Tat C treated human brain microvascular endothelial cells result in downregulation and dissociation of VE-PTP and SHP2 from VE-cadherin
CDH1	155807	NP_057852.2	Vpr	upregulates	10713718,	HIV-1 Vpr-expressing Jurkat T cell clones showed upregulated expression of cadherin, suggesting a role of Vpr in modulating the cell adhesion process
CDH1	155945	NP_057855.1	Vpu	interacts with	18256147,	HIV-1 Vpu reduces the beta-catenin interaction with E-cadherin and the Vpu-mediated dissociation of beta-catenin with E-cadherin enhances particle release
RNH1	155971	NP_057856.1	Envelope surface glycoprotein gp160, precursor	interacts with	27375898,	HIV-1 gp160 interacts with RNH1
RNH1	155030	NP_579881.1	nucleocapsid	interacts with	22190034,	HIV-1 NC is identified to have a physical interaction with ribonuclease/angiogenin inhibitor 1 (RNH1) in human HEK293 and/or Jurkat cell lines by using affinity tagging and purification mass spectrometry analyses
RNH1	155348	NP_705926.1	retropepsin	cleaves	22944692,	Positional proteomics analysis identifies the cleavage of human ribonuclease/angiogenin inhibitor 1 (RNH1) at amino acid residues 445-446 by the HIV-1 protease
RNH1			HIV-1 virus replication	enhanced by expression of human gene	18854154,	Knockdown of ribonuclease/angiogenin inhibitor 1 (RNH1) by siRNA inhibits the early stages of HIV-1 replication in 293T cells infected with VSV-G pseudotyped HIV-1
SELL	155971	NP_579894.2	Envelope surface glycoprotein gp120	downregulates	10449768, 14576059, 22842622	CD4 ligation by HIV-1 gp120 induces metalloproteinase-dependent L-selectin downregulation in primary resting CD4+ T cells
SELL	155971	NP_579894.2	Envelope surface glycoprotein gp120	downregulates	14576059,	L-selectin downregulation induced by HIV-1 gp120 is completely reversed by AMD3100 (a CXCR4 antagonist), but not SDF-1 alpha
SELL	156110	NP_057857.2	Nef	downregulates	25275127, 25822027	Both HIV-1 Nef and Vpu downregulate the cell surface expression of selectin L (CD62L)
SELL	156110	NP_057857.2	Nef	downregulates	25822027,	HIV-1 Nef mutants L37Q, 62EEEE/AAAA65, P72A/P75A, P78L, and F191A partially impair to downregulate CD62L in Jurkat cells
SELL	155871	NP_057853.1	Tat	upregulates	24667918,	Microarray analysis indicates HIV-1 Tat-induced upregulation of selectin L (SELL) in primary human brain microvascular endothelial cells
SELL	155945	NP_057855.1	Vpu	downregulates	25275127, 25822027	Both HIV-1 Nef and Vpu downregulate the cell surface expression of selectin L (CD62L)
SELL	155945	NP_057855.1	Vpu	inhibits	25822027,	HIV-1 Vpu inhibits the transport of newly synthesized CD62L molecules toward the cell surface of Jurkat cells
PKM	155971	NP_579894.2	Envelope surface glycoprotein gp120	complexes with	23125841,	Tandem affinity purification and mass spectrometry analysis identify pyruvate kinase of muscle (PKM), HIV-1 Gag, Gag/Pol, gp120, and Nef incorporated into staufen1 RNP complexes isolated from HIV-1-expressing cells
PKM	155971	NP_057856.1	Envelope surface glycoprotein gp160, precursor	interacts with	22190034,	HIV-1 gp160 is identified to have a physical interaction with pyruvate kinase, muscle (PKM2) in human HEK293 and/or Jurkat cell lines by using affinity tagging and purification mass spectrometry analyses
PKM	155030	NP_057850.1	Pr55(Gag)	complexes with	23125841,	Tandem affinity purification and mass spectrometry analysis identify pyruvate kinase of muscle (PKM), HIV-1 Gag, Gag/Pol, gp120, and Nef incorporated into staufen1 RNP complexes isolated from HIV-1-expressing cells
PKM	155348	NP_057849.4	Gag-Pol	complexes with	23125841,	Tandem affinity purification and mass spectrometry analysis identify pyruvate kinase of muscle (PKM), HIV-1 Gag, Gag/Pol, gp120, and Nef incorporated into staufen1 RNP complexes isolated from HIV-1-expressing cells
PKM	155348	NP_705926.1	retropepsin	cleaves	22944692,	Positional proteomics analysis identifies the cleavage of human pyruvate kinase, muscle (PKM) at amino acid residues 306-307 and 359-361 by the HIV-1 protease
PKM	156110	NP_057857.2	Nef	complexes with	23125841,	Tandem affinity purification and mass spectrometry analysis identify pyruvate kinase of muscle (PKM), HIV-1 Gag, Gag/Pol, gp120, and Nef incorporated into staufen1 RNP complexes isolated from HIV-1-expressing cells
PKM	155871	NP_057853.1	Tat	upregulates	23166591, 23364796	Expression of HIV-1 Tat upregulates the abundance of pyruvate kinase, muscle (PKM) in the nucleoli of Jurkat T-cells
PKM	155807	NP_057852.2	Vpr	upregulates	23874603,	A stable-isotope labeling by amino acids in cell culture coupled with mass spectrometry-based proteomics identifies upregulation of pyruvate kinase, muscle (PKM) expression by HIV-1 Vpr in Vpr transduced macrophages
HSP90B1	155971	NP_057856.1	Envelope surface glycoprotein gp160, precursor	interacts with	27375898,	HIV-1 gp160 interacts with HSP90B1; predicted interaction to be within the endoplasmic reticulum and function as chaperone for endoplasmic reticulum-associated degradation
HSP90B1	156110	NP_057857.2	Nef	cooperates with	24204260,	Microvesicles, which contaminate purified HIV-1 inocula due to similar size and density, contain viral protein Nef and cellular proteins HSP90alpha and HSP90beta that are capable of potent stimulation of dendritic cells maturation and ICAM-1 expression
HSP90B1	156110	NP_057857.2	Nef	requires	25496667,	Genome-wide shRNA screening identifies HSP90B1, which is required for HIV-1 Nef-induced downregulation of CD4 in HeLa CD4+ cells
HSP90B1	155908	NP_057854.1	Rev	interacts with	22174317,	HIV-1 Rev interacting protein, heat shock protein 90kDa beta (HSP90B1), is identified by the in-vitro binding experiments involving cytosolic or nuclear extracts from HeLa cells. The interaction of Rev with HSP90B1 is increased by RRE
NME1	155348	NP_705927.1	reverse transcriptase	complexes with	25766862,	HIV-1 RT forms a complex with NDPKA in vitro
NME1			HIV-1 virus replication	enhanced by expression of human gene	19266025,	SiRNA-mediated knockdown of one of the components of the SET complex, NM23-H1, inhibits HIV-1 infection with significantly reduced levels of integrated HIV-1 DNA and viral production in HeLa-CD4 cells
PECAM1	155871	NP_057853.1	Tat	upregulates	23301033,	HIV-1 Tat enhances vIL-6-induced angiogenesis and tumorigenesis of fibroblasts and human endothelial cells, which correlates with upregulation of CD31, CD34, SMA, VEGF, b-FGF, and cyclin D1 expression
PECAM1	155807	NP_057852.2	Vpr	upregulates	23874603,	A stable-isotope labeling by amino acids in cell culture coupled with mass spectrometry-based proteomics identifies upregulation of platelet/endothelial cell adhesion molecule 1 (PECAM1, PECA1) expression by HIV-1 Vpr in Vpr transduced macrophages
VCL	155030	NP_579876.2	matrix	interacts with	22017400,	HIV-1 MA co-localizes with beta2 integrin, alphaM and alphaX integrins in the intracellular thick electron-dense membrane compartments, which contain talin, vinculin and paxillin that connect the integrin complexes to the actin cytoskeleton
VCL	156110	NP_057857.2	Nef	co-localizes with	25527710, 25745180	HIV-1 Nef co-localizes with vinvulin inside podosomes in human monocyte-derived macrophages
VCL	156110	NP_057857.2	Nef	polarizes	14597672,	HIV-1 Nef induces polarization of vinculin, a molecule important for dendritic cell (DC) adhesion, motility, and maturation, at one pole of DCs corresponding to the substrate-adhering portion or leading edge of the cell
LBP	155871	NP_057853.1	Tat	upregulates	24667918,	Microarray analysis indicates HIV-1 Tat-induced upregulation of lipopolysaccharide binding protein (LBP) in primary human brain microvascular endothelial cells
ATF6	155871	NP_057853.1	Tat	induces	25409632,	HIV-1 Tat induces endoplasmic reticulum (ER) stress response proteins CASP12 (Caspase 12), DDIT3 (CHOP), ROS1, ERN-1 (p-IRE1), EIF2AK3 (p-PERK), and ATF6 in human brain microvascular endothelial cells (HBMECs)
CR2	155971	NP_579894.2	Envelope surface glycoprotein gp120	binds	8474169, 21161615	HIV-1 particles (gp120/gp41) bind to MT-2 (CD4+ CR2+) and Raji-3 (CD4- CR2+) cells but not to CEM (CD4+ CR2-) cells, suggesting that the virus binds to CR2 independently of CD4
CR2	155971	NP_057856.1	Envelope surface glycoprotein gp160, precursor	interacts with	15603708, 21161615	The activation of mitogen-activated protein kinases (MAPKs, including ERK, JNK, and p38MAPK) is induced by incubation of HIV-1 gp160 with CD4+complement receptor type 2 (CR2)+ cells
CR2	155030	NP_579880.1	capsid	localized by	26623655,	HIV-1 CA p24 is localized to recycling endosomes by CR2 (CD21) and soluble CD21 frees virus from follicular dendritic cells from lymph nodes of HIV infected patients
CR2	155459	NP_057851.1	Vif	downregulates	23333304,	HIV-1 Vif downregulates the expression of complement component receptor 2 (CR2) in Vif-expression T cells

CR2			HIV-1 virus replication	enhanced by expression of human gene	18976975,	Knockdown of complement component receptor 2 (CR2) by siRNA inhibits HIV-1 replication in HeLa P4/R5 cells
CR2			HIV-1 virus replication	inhibited by expression of human gene	26623655,	HIV-1 transmission to CD4+ T cells from follicular dendritic cells from lymph nodes of infected patients is inhibited by soluble CD21
FLNA	155971	NP_579894.2	Envelope surface glycoprotein gp120	binds	19366992,	HIV-1 gp120 promotes filamin binding to both CD4 and CXCR4
FLNA	155971	NP_579894.2	Envelope surface glycoprotein gp120	complexes with	23125841,	Tandem affinity purification and mass spectrometry analysis identify filamin A (FLNA), HIV-1 Gag, Gag/Pol, gp120, and Nef incorporated into staufen1 RNP complexes isolated from HIV-1-expressing cells
FLNA	155971	NP_579894.2	Envelope surface glycoprotein gp120	interacts with	17572668,	Filamin-A-dependent activation of the RhoA-ROCK-LIMK-cofilin pathway is a major event in HIV-1 gp120-induced receptor clustering
FLNA	155030	NP_057850.1	Pr55(Gag)	binds	21705339, 22004035	HIV-1 Gag binds to filamin A through the CA domain, which leads to facilitate Gag trafficking to the plasma membrane
FLNA	155030	NP_057850.1	Pr55(Gag)	co-localizes with	21705339, 22004035	HIV-1 Gag co-localizes with filamin A in mammalian cells. Depletion of filamin A inhibits HIV-1 particle release and induces Gag accumulation at the LE/MVB compartment
FLNA	155030	NP_057850.1	Pr55(Gag)	complexes with	23125841,	Tandem affinity purification and mass spectrometry analysis identify filamin A (FLNA), HIV-1 Gag, Gag/Pol, gp120, and Nef incorporated into staufen1 RNP complexes isolated from HIV-1-expressing cells
FLNA	155030	NP_057850.1	Pr55(Gag)	incorporates	25631074,	Cellular biotinylated filamin A, alpha (FLNA) protein is incorporated into HIV-1 Gag virus-like particles
FLNA	155348	NP_057849.4	Gag-Pol	complexes with	23125841,	Tandem affinity purification and mass spectrometry analysis identify filamin A (FLNA), HIV-1 Gag, Gag/Pol, gp120, and Nef incorporated into staufen1 RNP complexes isolated from HIV-1-expressing cells
FLNA	155348	NP_705926.1	retropepsin	cleaves	12119179,	A number of focal adhesion plaque proteins are specifically cleaved by HIV-1 protease, including fimbrin, focal adhesion plaque kinase (FAK), talin, and, to a lesser extent, filamin, spectrin and fibronectin
FLNA	156110	NP_057857.2	Nef	complexes with	23125841,	Tandem affinity purification and mass spectrometry analysis identify filamin A (FLNA), HIV-1 Gag, Gag/Pol, gp120, and Nef incorporated into staufen1 RNP complexes isolated from HIV-1-expressing cells
FLNA	155871	NP_057853.1	Tat	interacts with	25496916,	Filamin A, alpha (FLNA) is identified to interact with HIV-1 Tat mutant Nullbasic in HeLa cells by LC MS/MS
FLNA	155871	NP_057853.1	Tat	upregulates	23166591,	Expression of HIV-1 Tat upregulates the abundance of filamin A, alpha (FLNA) in the nucleoli of Jurkat T-cells
FLNA	155807	NP_057852.2	Vpr	upregulates	27114546,	HIV-1 Vpr upregulates FLNA in HeLa cells within 12 hours of exposure
HNRNPA2B1	155971	NP_579894.2	Envelope surface glycoprotein gp120	complexes with	23125841,	Tandem affinity purification and mass spectrometry analysis identify heterogeneous nuclear ribonucleoprotein A2/B1 (HNRNPA2B1), HIV-1 Gag, Gag/Pol, gp120, and Nef incorporated into staufen1 RNP complexes isolated from HIV-1-expressing cells
HNRNPA2B1	155971	NP_579894.2	Envelope surface glycoprotein gp120	regulated by	22187150,	Depletion of hnRNP A1 and A2 increase expression of viral structural proteins Gag, Env, and gp120
HNRNPA2B1	155971	NP_057856.1	Envelope surface glycoprotein gp160, precursor	regulated by	22187150,	Depletion of hnRNP A1 and A2 increase expression of viral structural proteins Gag, Env, and gp120
HNRNPA2B1	155030	NP_057850.1	Pr55(Gag)	complexes with	23125841, 24690621	Tandem affinity purification and mass spectrometry analysis identify heterogeneous nuclear ribonucleoprotein A2/B1 (HNRNPA2B1), HIV-1 Gag, Gag/Pol, gp120, and Nef incorporated into staufen1 RNP complexes isolated from HIV-1-expressing cells
HNRNPA2B1	155030	NP_057850.1	Pr55(Gag)	regulated by	22187150,	Depletion of hnRNP A1 and A2 increase expression of viral structural proteins Gag, Env, and gp120
HNRNPA2B1	155348	NP_057849.4	Gag-Pol	complexes with	23125841,	Tandem affinity purification and mass spectrometry analysis identify heterogeneous nuclear ribonucleoprotein A2/B1 (HNRNPA2B1), HIV-1 Gag, Gag/Pol, gp120, and Nef incorporated into staufen1 RNP complexes isolated from HIV-1-expressing cells
HNRNPA2B1	155348	NP_705928.1	integrase	binds	20016921,	Using acetylated HIV-1 IN as bait in yeast two-hybrid screening identifies translation regulatory and RNA binding proteins eIF3h, eEF1A-1, and hnRNPA2 as IN-binding partners
HNRNPA2B1	155348	NP_705926.1	retropepsin	cleaves	22944692,	Positional proteomics analysis identifies the cleavage of human heterogeneous nuclear ribonucleoprotein A2/B1 (HNRNPA2B1) at amino acid residues 135-136 by the HIV-1 protease
HNRNPA2B1	156110	NP_057857.2	Nef	complexes with	23125841,	Tandem affinity purification and mass spectrometry analysis identify heterogeneous nuclear ribonucleoprotein A2/B1 (HNRNPA2B1), HIV-1 Gag, Gag/Pol, gp120, and Nef incorporated into staufen1 RNP complexes isolated from HIV-1-expressing cells
HNRNPA2B1	155908	NP_057854.1	Rev	interacts with	22174317,	HIV-1 Rev interacting protein, heterogeneous nuclear ribonucleoproteins A2/B1 (HNRNPA2B1), is identified by the in-vitro binding experiments involving cytosolic or nuclear extracts from HeLa. The interaction of Rev with HNRNPA2B1 is increased by RRE
HNRNPA2B1	155871	NP_057853.1	Tat	downregulates	23166591,	Expression of HIV-1 Tat downregulates the abundance of heterogeneous nuclear ribonucleoprotein A2/B1 (HNRNPA2B1) in the nucleoli of Jurkat T-cells
HNRNPA2B1	155459	NP_057851.1	Vif	regulated by	23255806,	Proteins hnRNP F, hnRNP H, and hnRNP A2/B1 bind to the intronic G run (G12-1: AGGGA) to regulate the levels of vif mRNA and Vif protein expression
HNRNPA2B1	155459	NP_057851.1	Vif	regulated by	25169827,	Proteins hnRNP F, hnRNP H, and hnRNP A2/B1 bind to the intronic G run (G13-2: AGGGA) to regulate the levels of vif and vpr mRNA, and the amounts of Vif and Vpr protein expression
HNRNPA2B1	155807	NP_057852.2	Vpr	regulated by	25169827,	Proteins hnRNP F, hnRNP H, and hnRNP A2/B1 bind to the intronic G run (G13-2: AGGGA) to regulate the levels of vif and vpr mRNA, and the amounts of Vif and Vpr protein expression
CFL1	155971	NP_579894.2	Envelope surface glycoprotein gp120	activates	18775311, 18808680, 20842205, 24778234	HIV-1 gp120-CXCR4 signaling triggers cofilin activation and actin reorganization, which are important for a post entry process leading to viral nuclear localization
CFL1	155971	NP_579894.2	Envelope surface glycoprotein gp120	induces phosphorylation of	23294842,	The N-terminal leucine-rich repeat fragment of Slit2 inhibits HIV-1 gp120-induced phosphorylation of both LIMK1 and cofilin
CFL1	155971	NP_579894.2	Envelope surface glycoprotein gp120	induces phosphorylation of	24778234,	CCR5 expression inhibits HIV-1 gp120-induced LIMK1 activation and cofilin phosphorylation in CD4/CXCR4 expressing 293T cells
CFL1	155971	NP_579894.2	Envelope surface glycoprotein gp120	interacts with	17572668, 18808680	Filamin-A-dependent activation of the RhoA-ROCK-LIMK-cofilin pathway is a major event in HIV-1 gp120-induced receptor clustering
CFL1	155030	NP_057850.1	Pr55(Gag)	incorporates	8892894,	The cytoskeletal proteins ezrin, moesin, and cofilin are incorporated into HIV-1 particles, presumably through their interaction with actin which binds to the nucleocapsid domain of HIV-1 Gag
CFL1	156110	NP_057857.2	Nef	induces phosphorylation of	19683683, 20147394	HIV-1 Nef inactivates cofilin by inducing its hyperphosphorylation via association with PAK2 activity
CFL1	156110	NP_057857.2	Nef	induces phosphorylation of	21923909,	HIV-1 Nef-induced LIMK1 activation and CFL1 phosphorylation are required for Nef-mediated inhibition of retinoid receptor function
CFL1	156110	NP_057857.2	Nef	induces phosphorylation of	22537596,	The HIV-1 Nef highly conserved valine-glycine-phenylalanine amino acid triplet (VGF) motif is important for Nef-PAK2 association and cofillin hyper-phosphorylation
CFL1	155871	NP_057853.1	Tat	downregulates	16526095,	In Jurkat cells expressing HIV-1 Tat, decreased expression levels are found for basic cytoskeletal proteins such as actin, beta-tubulin, annexin, cofilin, gelsolin, and Rac/Rho-GDI complex
CALR	155971	NP_579894.2	Envelope surface glycoprotein gp120	interacts with	22190034,	HIV-1 gp120 is identified to have a physical interaction with calreticulin (CALR) in human HEK293 and/or Jurkat cell lines by using affinity tagging and purification mass spectrometry analyses
CALR	155971	NP_057856.1	Envelope surface glycoprotein gp160, precursor	binds	8550632,	The ubiquitous eukaryotic protein calreticulin binds to newly synthesized HIV-1 gp160, suggesting that calreticulin might act as a chaperone
CALR	155971	NP_057856.1	Envelope surface glycoprotein gp160, precursor	interacts with	22190034,	HIV-1 gp160 is identified to have a physical interaction with calreticulin (CALR) in human HEK293 and/or Jurkat cell lines by using affinity tagging and purification mass spectrometry analyses
CALR	155908	NP_057854.1	Rev	interacts with	22174317,	HIV-1 Rev interacting protein, calreticulin (CALR), is identified by the in-vitro binding experiments involving cytosolic or nuclear extracts from HeLa cells. The interaction of Rev with CALR is increased by RRE

CALR	155807	NP_057852.2	Vpr	upregulates	23874603,	A stable-isotope labeling by amino acids in cell culture coupled with mass spectrometry-based proteomics identifies upregulation of calreticulin (CALR) expression by HIV-1 Vpr in Vpr transduced macrophages
PSMB4	155348	NP_705928.1	integrase	degraded by	10893419,	Proteasomal degradation of HIV-1 integrase in mammalian cells occurs by the N-end rule pathway
PSMB4	156110	NP_057857.2	Nef	binds	9344905,	Amino acids 34-143 of HIV-1 Nef mediate its binding to amino acids 73-249 of the HsN3 proteasomal subunit
PSMB4	156110	NP_057857.2	Nef	downregulates	9344905, 23559827	HIV-1 Nef was found to markedly downregulate intracellular levels of both a co-expressed HsN3 and the endogenous simian homologue, suggesting Nef may alter proteasome function in infected cells
PSMB4	155871	NP_057853.1	Tat	enhances	9079628,	HIV-1 Tat slightly enhances the activity of the purified 26 S proteasome
PSMB4	155871	NP_057853.1	Tat	inhibits	14550573,	HIV-1 Tat binds to the alpha2, alpha4, alpha6, alpha7, beta1, beta2, beta3, beta5, beta6, beta7, LMP7/beta5i, and MECL1/beta2i subunits of the proteasome 20 S core structure and can inhibit cellular proteasome function
PSMB4	155871	NP_057853.1	Tat	inhibits	9079628, 12419264, 14550573	HIV-1 Tat inhibits the peptidase activity of the 20 S proteasome and interferes with the formation of the 20 S proteasome-11 S regulator complex
PSMB4	155871	NP_057853.1	Tat	interacts with	12419264,	Amino acids Lys51, Arg52, and Asp67 of HIV-1 Tat represent the proteasome binding site of Tat, and Tat amino acids 37-72 are necessary for proteasomal interaction and suppression of 11 S regulator-mediated antigen presentation
PSMB4	155459	NP_057851.1	Vif	interacts with	9811770, 9846577, 12167863, 12719574, 12750511, 12808465, 12808466, 12809610, 12830140, 12840737, 12859895, 12914693, 12920286, 12970355, 14527406, 14528300, 14528301, 14557625, 14564014, 14614829	HIV-1 Vif binds to the cellular cytidine deaminase APOBEC3G and targets it for degradation through an interaction with the proteasome, thereby inhibiting APOBEC3G mediated restriction of HIV-1 replication
GRN	155871	NP_057853.1	Tat	binds	10079180,	The cysteine rich region of HIV-1 Tat (amino acids 21-37) mediates the binding of Tat to granulin amino acids 206-337 (granulin regions B+A) suggesting a role for granulin growth factors as biologically important extracellular Tat co-factors
GRN	155871	NP_057853.1	Tat	inhibited by	12588988, 15653695, 20054825	Granulin forms stable complexes with cyclin T1 and HIV-1 Tat and inhibits Tat transactivation of the viral LTR promoter
TKT	155348	NP_705926.1	retropepsin	cleaves	22944692,	Positional proteomics analysis identifies the cleavage of human transketolase (TKT) at amino acid residues178-179 by the HIV-1 protease
TKT	155807	NP_057852.2	Vpr	downregulates	23874603,	A stable-isotope labeling by amino acids in cell culture coupled with mass spectrometry-based proteomics identifies downregulation of transketolase (TKT) expression by HIV-1 Vpr in Vpr transduced macrophages
PDIA3	155971	NP_579894.2	Envelope surface glycoprotein gp120	cleaved by	12218051, 12218052, 15644496, 16182193, 22230366	Protein-disulfide isomerase (PDI) cleaves disulfide bonds in recombinant HIV-1 envelope glycoprotein gp120, and gp120 bound to the surface receptor CD4 undergoes a disulfide reduction that is prevented by PDI inhibitors
PDIA3	155971	NP_579894.2	Envelope surface glycoprotein gp120	co-localizes with	24825317,	HIV-1 gp120/MBL complex co-localizes with the ER marker ERp57 and the Golgi marker p230 at subcellular perinuclear compartments in neuronal cells
PDIA3	155971	NP_579894.2	Envelope surface glycoprotein gp120	interacts with	20458450,	The disulfide cross-linking interaction between gp120 and PDI is enhanced by CD4 protein
PDIA3	155971	NP_579894.2	Envelope surface glycoprotein gp120	interacts with	23206338,	PDI is predominantly involved in HIV-1 entry and infection of the T cell line PM-1 and PHA-stimulated primary T lymphocytes, suggesting the preferential use of PDI relevant to the HIV-1 entry and establishment of virus reservoirs in resting CD4+ cells
PDIA3	155971	NP_057856.1	Envelope surface glycoprotein gp160, precursor	interacts with	22190034,	HIV-1 gp160 is identified to have a physical interaction with protein disulfide isomerase family A, member 3 (PDIA3) in human HEK293 and/or Jurkat cell lines by using affinity tagging and purification mass spectrometry analyses
PDIA3	155971	NP_057856.1	Envelope surface glycoprotein gp160, precursor	interacts with	27375898,	HIV-1 gp160 interacts with PDIA3; predicted interaction to be within the endoplasmic reticulum and function as a thioredoxin reductase
PDIA3	155971	NP_057856.1	Envelope surface glycoprotein gp160, precursor	processed by	17301129,	Treatment of trimeric HIV-1 rgp140 with protein disulfide isomerase yields monomers by disruption of the intermolecular disulfide bonds
PDIA3	155971	NP_579895.1	Envelope transmembrane glycoprotein gp41	interacts with	22190034,	HIV-1 gp41 is identified to have a physical interaction with protein disulfide isomerase family A, member 3 (PDIA3) in human HEK293 and/or Jurkat cell lines by using affinity tagging and purification mass spectrometry analyses
PDIA3	155348	NP_705926.1	retropepsin	cleaves	22944692,	Positional proteomics analysis identifies the cleavage of human protein disulfide isomerase family A, member 3 (PDIA3) at amino acid residues 27-28 by the HIV-1 protease
PDIA3	155807	NP_057852.2	Vpr	downregulates	23874603,	A stable-isotope labeling by amino acids in cell culture coupled with mass spectrometry-based proteomics identifies downregulation of protein disulfide isomerase A3 (PDIA3) expression by HIV-1 Vpr in Vpr transduced macrophages
PDIA3			HIV-1 virus replication	enhanced by expression of human gene	18976975,	Knockdown of protein disulfide isomerase family A, member 3 (PDIA3) by siRNA inhibits HIV-1 replication in HeLa P4/R5 cells
PPIF	155030	NP_579880.1	capsid	interacts with	25505242,	The interaction of HIV-1 CA with human cellular peptidylprolyl isomerase F protein (PPIF, cyclophilin F) is identified by yeast two-hybrid screen
PRDX2	155348	NP_705926.1	retropepsin	cleaves	22944692,	Positional proteomics analysis identifies the cleavage of human peroxiredoxin 2 (PRDX2) at amino acid residues 47-48 by the HIV-1 protease
MAN1A1	155971	NP_579894.2	Envelope surface glycoprotein gp120	processed by	12560567,	Specific alterations of the N-linked carbohydrates on HIV-1 gp120 and gp41 by glucosidases and mannosidase inhibitors can enhance mannose-binding lectin (MBL)-mediated neutralization of virus by strengthening the interaction of HIV-1 with MBL
MAN1A1	155971	NP_579894.2	Envelope surface glycoprotein gp120	processed by	2283726, 2355006, 2406237, 2542563, 2649653, 2829950, 8218172, 8892864	HIV-1 gp120 N-linked oligosaccharides are processed by manosidase I and II in the Golgi complex
MAN1A1	155971	NP_057856.1	Envelope surface glycoprotein gp160, precursor	processed by	2187500, 2541446, 2542563, 2649653, 2829950, 8673525, 9109416, 11530211, 18215327, 18314154, 18330979	Oligosaccharide side-chains of HIV-1 gp160 are processed by glycosidase I and II, mannosidase I and II, acetylglucosaminyl transferase I and II, and fucosyl, galactosyl and sialyl transferases in both the endoplasmic reticulum and golgi apparatus
MAN1A1	155971	NP_579895.1	Envelope transmembrane glycoprotein gp41	processed by	1736542, 2829950, 3099781, 3264072, 8093218	Mannose-containing, N-linked oligosaccharide side-chains of HIV-1 gp41 are involved in the initial stage of infection by HIV-1; glycosylation inhibitors block virus-cell and cell-cell fusion and release of the virions
MAN1A1			HIV-1 virus replication	enhanced by expression of human gene	18854154,	Knockdown of mannosidase, alpha, class 1A, member 1 (MAN1A1) by siRNA inhibits the early stages of HIV-1 replication in 293T cells infected with VSV-G pseudotyped HIV-1
RDX	155971	NP_579894.2	Envelope surface glycoprotein gp120	relocalizes	15818415,	Treatment of CD4+ T cells with HIV-1 gp120 induces CD95-mediated apoptosis, CD95/ERM protein (ezrin, radixin, moesin) colocalization and stable ezrin phosphorylation
RDX	155030	NP_057850.1	Pr55(Gag)	co-localizes with	24760896,	HIV-1 Gag co-localizes with ezrin-radixin-moesin proteins at polarized HIV-1 assembly sites in human T cells
RDX	155807	NP_057852.2	Vpr	downregulates	17349711,	HIV-1 Vpr-induced downregulation of sodium hydrogen exchanger, isoform 1 (NHE1), in Vpr(+) virus infected cells leads to acidification of cells, loss of ezrin, radixin and moesin (ERM) protein complex and decrease of AKT phosphorylation
MYH9	155971	NP_579894.2	Envelope surface glycoprotein gp120	upregulates	24162774,	HIV-1 gp120 upregulates the expression of myosin, heavy chain 9, non-muscle (MYH9) in human B cells
MYH9	155030	NP_057850.1	Pr55(Gag)	interacts with	25010285,	Interaction of HIV-1 Gag with myosin, heavy chain 9, non-muscle (MYH9) is identified in a series of six affinity purification/mass spectrometry screens
MYH9	155348	NP_705926.1	retropepsin	cleaves	8424456,	HIV-1 protease cleaves human myosin heavy chain in vitro

MYH9	156110	NP_057857.2	Nef	downregulates	24949636,	MYH9 is downregulated by HIV-1 infection, which indicates that MYH9 downregulation is likely part of the Nef-mediated signaling cascade that includes RhoA downregulation
MYH9	155871	NP_057853.1	Tat	interacts with	23732912,	The centripetal and lateral movements of the HIV-1 Tat protein transduction domain are linked to the integrity of myosin II-based actin contraction in HeLa cells
MYH9	155871	NP_057853.1	Tat	interacts with	25496916,	Myosin, heavy chain 9, non-muscle (MYH9, NMHC-IIA) is identified to interact with HIV-1 Tat mutant Nullbasic in HeLa cells by LC MS/MS
MYH9	155871	NP_057853.1	Tat	upregulates	23166591,	Expression of HIV-1 Tat upregulates the abundance of myosin, heavy chain 9, non-muscle (MYH9) in the nucleoli of Jurkat T-cells
MYH9	155807	NP_057852.2	Vpr	upregulates	23874603,	A stable-isotope labeling by amino acids in cell culture coupled with mass spectrometry-based proteomics identifies upregulation of myosin, heavy chain 9 (MYH9, non-muscle) expression by HIV-1 Vpr in Vpr transduced macrophages
MDH2	155348	NP_705926.1	retropepsin	cleaves	22944692,	Positional proteomics analysis identifies the cleavage of human malate dehydrogenase 2, NAD, mitochondrial (MDH2) at amino acid residues 94-95 by the HIV-1 protease
MDH2	155807	NP_057852.2	Vpr	downregulates	23874603,	A stable-isotope labeling by amino acids in cell culture coupled with mass spectrometry-based proteomics identifies downregulation of malate dehydrogenase 2 (MDH2) expression by HIV-1 Vpr in Vpr transduced macrophages
PAPOLA	155871	NP_057853.1	Tat	regulates	8491200,	The poly(A) site in the HIV-1 5'-LTR is occluded in a Tat-dependent manner, suggesting a role for Tat in regulating this nucleotide signal
PAPOLA	155807	NP_057852.2	Vpr	decreases phosphorylation of	11878934,	HIV-1 Vpr dephosphorylates poly(A) polymerase (PAP), presumably through inhibition of the p34cdc2/cyclin B kinase complex, leading to enhanced PAP activity
ARHGDIB	155807	NP_057852.2	Vpr	modulates	19655254,	The proteomic assay from Vpr-expressing HTLV-1 transformed cells reveals apoptosis related protein changes, such as CASP3 activity indicator proteins (vimentin and Rho GDP-dissociation inhibitor 2)
RAD23A	155807	NP_057852.2	Vpr	binds	11426943, 19458171, 20012529	Overexpression of HHR23A causes apoptosis of cells, suggesting that Vpr binding to HHR23A may be involved in Vpr-induced apoptosis
RAD23A	155807	NP_057852.2	Vpr	binds	24318982,	NMR chemical shift analysis demonstrates that HIV-1 Vpr binds hHR23A through the contact surfaces on the XPCB (residues 232-286) and UBA2 (residues 316-363) domains of hHR23A
RAD23A	155807	NP_057852.2	Vpr	binds	24318982,	Residues Q249, L255, L259, N266, and N285 in XPCB domain and residues R326, G331, F332, E334, L336, F342, K346, E348, N349, A351, N353, and Q358 in UBA2 domain of hHR23A are involved in the binding to HIV-1 Vpr
RAD23A	155807	NP_057852.2	Vpr	binds	9371639, 9636371, 9846873, 11087358, 11259200, 12079361, 16120388, 18514189, 19458171, 20012529, 20614012, 21318276	Binding of HIV-1 Vpr (amino acids 25-77) to the UBA(2) domain of RAD23A (HHR23A) (amino acids 319-363) affects the cell cycle arrest induced by Vpr
RAD23A	155807	NP_057852.2	Vpr	complexes with	24318982,	A di-Ub(K48)-hHR23A-Vpr ternary complex is formed with Lys-48-linked di-ubiquitin binding to the UBA1 domain in the Vpr-hHR23A complex
RAD23A	155807	NP_057852.2	Vpr	cooperates with	20614012,	HIV-1 Vpr promotes cellular protein polyubiquitination via hHR23A. Depletion of hHR23A significantly reduces HIV-1 replication in a Vpr-dependent manner
RAD23A	155807	NP_057852.2	Vpr	inhibited by	9371639, 9636371, 11259200, 20012529	Two reports indicate overexpression of HHR23A can inhibit HIV-1 Vpr-induced cell cycle arrest, while a third report indicates Vpr binding to HHR23A does not correlate with the ability of Vpr to induce cell cycle arrest
RAD23A	155807	NP_057852.2	Vpr	inhibits	11196199, 19458171, 20012529	Co-expression of HIV-1 Vpr with HHR23A neutralizes inhibitory effects of HHR23A on p53 transcriptional activity
RAD23A			HIV-1 virus replication	enhanced by expression of human gene	18976975,	Knockdown of RAD23 homolog A by siRNA inhibits HIV-1 replication in HeLa P4/R5 cells
RAB10	155971	NP_579894.2	Envelope surface glycoprotein gp120	complexes with	23125841,	Tandem affinity purification and mass spectrometry analysis identify ras-related protein Rab-10 (RAB10), HIV-1 Gag, Gag/Pol, gp120, and Nef incorporated into staufen1 RNP complexes isolated from HIV-1-expressing cells
RAB10	155971	NP_057856.1	Envelope surface glycoprotein gp160, precursor	interacts with	27375898,	HIV-1 gp160 interacts with RAB10; predicted interaction to be relevant to vesicular transport/membrane trafficking
RAB10	155030	NP_057850.1	Pr55(Gag)	complexes with	23125841,	Tandem affinity purification and mass spectrometry analysis identify ras-related protein Rab-10 (RAB10), HIV-1 Gag, Gag/Pol, gp120, and Nef incorporated into staufen1 RNP complexes isolated from HIV-1-expressing cells
RAB10	155348	NP_057849.4	Gag-Pol	complexes with	23125841,	Tandem affinity purification and mass spectrometry analysis identify ras-related protein Rab-10 (RAB10), HIV-1 Gag, Gag/Pol, gp120, and Nef incorporated into staufen1 RNP complexes isolated from HIV-1-expressing cells
RAB10	156110	NP_057857.2	Nef	complexes with	23125841,	Tandem affinity purification and mass spectrometry analysis identify ras-related protein Rab-10 (RAB10), HIV-1 Gag, Gag/Pol, gp120, and Nef incorporated into staufen1 RNP complexes isolated from HIV-1-expressing cells
RAP1B	155030	NP_579880.1	capsid	downregulated by	24586238,	Prostaglandin E2-mediated HIV-1 inhibition requires the EPAC/RAP/RhoA signaling pathway by downregulation of HIV-1 CA production
RAP1B			HIV-1 virus replication	enhanced by expression of human gene	18187620,	Knockdown of RAP1B, member of RAS oncogene family (RAP1B) by siRNA inhibits HIV-1 replication in HeLa-derived TZM-bl cells
B2M	156110	NP_057857.2	Nef	downregulates	25275127,	Both HIV-1 Nef and Vpu downregulate the cell surface expression of beta-2-microglobulin (B2M)
B2M	155871	NP_057853.1	Tat	downregulates	9751712, 10199391, 21085635	HIV-1 Tat represses transcription of the beta 2-microglobulin (B2M) promoter, thereby downregulating B2M expression, suggesting a mechanism by which HIV-1 could prevent cell surface expression of the MHC class I complex and avoid immune detection
B2M	155945	NP_057855.1	Vpu	downregulates	25275127,	Both HIV-1 Nef and Vpu downregulate the cell surface expression of beta-2-microglobulin (B2M)
ACTA2	155971	NP_579894.2	Envelope surface glycoprotein gp120	induces reorganization of	18775311, 22640593	HIV-1 gp120-CXCR4 signaling triggers cofilin activation and actin reorganization, which are important for a post entry process leading to viral nuclear localization
ACTA2	155971	NP_579894.2	Envelope surface glycoprotein gp120	induces reorganization of	22535526,	Syntenin-1 is recruited toward HIV-1 gp120/gp41-driven virus/cell and cell/cell contacts, associates with CD4, limits HIV-1-induced cell fusion and viral entry, and modulates gp120/gp41-triggered actin polymerization and PIP2 accumulation
ACTA2	155971	NP_579894.2	Envelope surface glycoprotein gp120	induces reorganization of	23294842,	The N-terminal leucine-rich repeat fragment of Slit2 inhibits HIV-1 gp120-induced actin polymerization in T cells
ACTA2	155971	NP_579894.2	Envelope surface glycoprotein gp120	induces reorganization of	23575248,	Gelsolin overexpression impairs HIV-1 gp120-induced cortical F-actin reorganization and capping and gp120-mediated CD4-CCR5 and CD4-CXCR4 redistribution in permissive lymphocytes
ACTA2	155971	NP_579894.2	Envelope surface glycoprotein gp120	interacts with	18443296,	Inducible T-cell kinase (ITK) affects viral entry and gp120-induced actin reorganization
ACTA2	155971	NP_579894.2	Envelope surface glycoprotein gp120	upregulates	22479424,	HIV-1 X4-tropic gp120 upregulates alpha-SMA (ACTA2) and collagen I alpha 1 expression via the ERK1/2 pathway in a CXCR4-dependent manner in activated human hepatic stellate cells
ACTA2	155971	NP_057856.1	Envelope surface glycoprotein gp160, precursor	interacts with	17360745, 17504171	Treatment of cells with actin-depolymerizing agents or tubulin polymerization inhibitors largely reduces the percentage of cells with capped HIV-1 Gag and Env, indicating an intact actin and tubulin cytoskeleton is required for efficient assembly of HIV-1
ACTA2	155971	NP_579895.1	Envelope transmembrane glycoprotein gp41	induces reorganization of	22535526,	Syntenin-1 is recruited toward HIV-1 gp120/gp41-driven virus/cell and cell/cell contacts, associates with CD4, limits HIV-1-induced cell fusion and viral entry, and modulates gp120/gp41-triggered actin polymerization and PIP2 accumulation
ACTA2	155971	NP_579895.1	Envelope transmembrane glycoprotein gp41	inhibits	10556093,	The interaction of the long cytoplasmic tail of HIV-1 gp41 with the carboxy-terminal regulatory domain of p115-RhoGEF inhibits p115-mediated actin stress fiber formation and activation of serum response factor (SRF)
ACTA2	155030	NP_057850.1	Pr55(Gag)	co-localizes with	23260110,	HIV-1 Gag, ITK, and F-actin are located in overlapping and discrete regions of T cell-T cell contact sites
ACTA2	155030	NP_057850.1	Pr55(Gag)	interacts with	17360745, 17504171	Treatment of cells with actin-depolymerizing agents or tubulin polymerization inhibitors largely reduces the percentage of cells with capped HIV-1 Gag and Env, indicating an intact actin and tubulin cytoskeleton is required for efficient assembly of HIV-1

ACTA2	155030	NP_057850.1	Pr55(Gag)	interacts with	23260110,	Tec kinase chemical inhibitors diminish the recruitment of ITK to the plasma membrane perturbing HIV-1 Gag-ITK co-localization, disrupting F-actin polymerization, and inhibiting HIV-1 release and replication
ACTA2	155030	NP_057850.1	Pr55(Gag)	requires	19883584, 21917091, 22004035, 22989508	HIV-1 Gag assembly and budding occur through an actin-driven mechanism
ACTA2	155030	NP_579876.2	matrix	interacts with	9841925, 17411366, 19639585	The localization of the HIV-1 reverse transcription complex to actin microfilaments is mediated by the interaction of a reverse transcription complex component (HIV-1 Matrix) with actin, but not vimentin (intermediate filaments) or tubulin (microtubules)
ACTA2	155030	NP_579881.1	nucleocapsid	binds	23017337,	HIV-1 NC-like aggregates are associated with dsDNA synthesis by HIV-1 RT and appear to efficiently bind to F-actin filaments, a property that may be involved in targeting complexes to the nuclear envelope
ACTA2	155030	NP_579881.1	nucleocapsid	binds	8661406, 8892894, 9971772, 10049817, 10074138, 11709093, 12009869	Mature HIV-1 Nucleocapsid, as well as the nucleocapsid domain of the HIV-1 Gag polyprotein, binds filamentous actin resulting in incorporation of actin into virus particles and enhancement of cell motility
ACTA2	155348	NP_705926.1	retropepsin	cleaves	1540415,	Actin, one of the most abundant proteins of the cell, is hydrolyzed by the human immunodeficiency virus type 1 (HIV-1) protease during acute infection of cultured human T lymphocytes
ACTA2	155348	NP_705926.1	retropepsin	cleaves	1540415, 1907279, 1991513, 8997639	HIV-1 protease cleaves actin in vitro at amino acid residues 66-67, 94-95, and 126-127
ACTA2	155348	NP_705927.1	reverse transcriptase	co-localizes with	9841925,	The localization of the HIV-1 reverse transcription complex to actin microfilaments is mediated by the interaction of a reverse transcription complex component (HIV-1 Matrix) with actin, but not vimentin (intermediate filaments) or tubulin (microtubules)
ACTA2	155348	NP_705927.1	reverse transcriptase	interacts with	23017337,	HIV-1 NC-like aggregates are associated with dsDNA synthesis by HIV-1 RT and appear to efficiently bind to F-actin filaments, a property that may be involved in targeting complexes to the nuclear envelope
ACTA2	156110	NP_057857.2	Nef	co-localizes with	22721673,	HIV-1 Nef co-localizes with F-actin and reorganizes F-actin assembly in the cortical regions of human podocyte
ACTA2	156110	NP_057857.2	Nef	downregulates	23071112,	HIV-1 Nef inhibits CXCL12 induced chemotaxis in Jurkat cells, monocytes, and PBMCs, which leads to marked downregulation of F-actin accumulation in cells
ACTA2	156110	NP_057857.2	Nef	inhibits	20147394,	HIV-1 Nef requires a PAK2 recruitment motif (F195/191I) for inhibition of actin remodeling and induction of cofilin hyperphosphorylation
ACTA2	156110	NP_057857.2	Nef	inhibits	21923909,	HIV-1 Nef induces loss of F-actin assembly and inhibits retinoid receptor-mediated transcription
ACTA2	156110	NP_057857.2	Nef	relocalizes	27560372,	HIV-1 NA7 and SF2 Nefs relocalizes ACTA1 and ACTB (F-actin); dependent upon the C-terminal aspartic acids in Nef
ACTA2	155871	NP_057853.1	Tat	downregulates	16526095,	In Jurkat cells expressing HIV-1 Tat, decreased expression levels are found for basic cytoskeletal proteins such as actin, beta-tubulin, annexin, cofilin, gelsolin, and Rac/Rho-GDI complex
ACTA2	155871	NP_057853.1	Tat	downregulates	23811015, 23875777	Treatment of primary hippocampal neurons with HIV-1 Tat produces a significant early reduction in F-actin labeled puncta. The cysteine rich domain (residues 22-37) of Tat is required for Tat-mediated reduction of F-actin labeled puncta
ACTA2	155871	NP_057853.1	Tat	induces rearrangement of	14694110,	HIV-1 Tat induces actin cytoskeletal rearrangements through p21-activated kinase 1 (PAK1) and downstream activation of the endothelial NADPH oxidase, an effect that is lost by introduction of mutations into the Tat cysteine-rich or basic domains
ACTA2	155871	NP_057853.1	Tat	interacts with	24742657,	Treatment with cannabinoids inhibits HIV-1 Tat-enhanced attachment of U937 cells to collagen IV, laminin, or ECM1 proteins, which is linked to the cannabinoid receptor type 2 and the modulation of beta1-integrin and actin distribution
ACTA2	155871	NP_057853.1	Tat	regulated by	22465675, 23178941	Uptake of the HIV-1 Tat protein is regulated by arrangement of the actin cytoskeleton in epithelial cells
ACTA2	155807	NP_057852.2	Vpr	downregulates	23874603,	A stable-isotope labeling by amino acids in cell culture coupled with mass spectrometry-based proteomics identifies downregulation of actin, alpha 2 (ACTA2) expression by HIV-1 Vpr in Vpr transduced macrophages
TUBA1B	155971	NP_579894.2	Envelope surface glycoprotein gp120	induces acetylation of	15103018, 16148047	The binding of HIV-1 gp120 to CD4+-permissive cells increases the level of acetylated alpha-tubulin in a CD4-dependent manner; overexpression of Histone Deacetylase 6 (HDAC6) inhibits the acetylation of alpha-tubulin and prevents HIV-1-cell fusion
TUBA1B	155971	NP_057856.1	Envelope surface glycoprotein gp160, precursor	interacts with	17360745,	Treatment of cells with actin-depolymerizing agents or tubulin polymerization inhibitors largely reduces the percentage of cells with capped HIV-1 Gag and Env, indicating an intact actin and tubulin cytoskeleton is required for efficient assembly of HIV-1
TUBA1B	155030	NP_057850.1	Pr55(Gag)	interacts with	17360745,	Treatment of cells with actin-depolymerizing agents or tubulin polymerization inhibitors largely reduces the percentage of cells with capped HIV-1 Gag and Env, indicating an intact actin and tubulin cytoskeleton is required for efficient assembly of HIV-1
TUBA1B	155348	NP_705928.1	integrase	interacts with	21167302,	Co-immunoprecipitation shows interaction of HIV-1 IN with alpha-tubulin
TUBA1B	155348	NP_705926.1	retropepsin	cleaves	22944692,	Positional proteomics analysis identifies the cleavage of human tubulin, alpha 1b (TUBA1B) at amino acid residues 67-68 and 202-203 by the HIV-1 protease
TUBA1B	155908	NP_057854.1	Rev	depolymerizes	10908577,	Rev acts to depolymerize microtubules that are formed by tubulin, an effect that is observed during HIV-1 infection
TUBA1B	155908	NP_057854.1	Rev	interacts with	22174317,	HIV-1 Rev interacting protein, TUBA1B, is identified by the in-vitro binding experiments involving cytosolic or nuclear extracts from HeLa cells
TUBA1B	155871	NP_057853.1	Tat	binds	12486001, 15331610	HIV-1 Tat (amino acids 36-39) binds tubulin alpha/beta dimers and polymerized microtubules leading to the alteration of microtubule dynamics and activation of a mitochondria-dependent apoptotic pathway that is facilitated by the Bcl-2 relative Bim
TUBA1B	155871	NP_057853.1	Tat	enhances polymerization of	15691386, 15698476, 18613978	HIV-1 Tat (specifically, amino acids 38-72), enhances tubulin polymerization and triggers the mitochondrial pathway to induce T cell apoptosis as shown in vitro by the release of cytochrome c from isolated mitochondria
TUBA1B	155871	NP_057853.1	Tat	modulates	23826228, 25328666	HIV-1 Tat K29A, K50R, and K51R lysine mutations downregulate the proportion of soluble tubulin in cells, while the majority of other lysine mutations upregulate the percentage of soluble tubulin compared with the wild-type
TUBA1B			HIV-1 virus replication	enhanced by expression of human gene	19460752,	Knockdown of tubulin, alpha 1b (TUBA1B) by shRNA library screening inhibits HIV-1 replication in cultured Jurkat T-cells
HBB			HIV-1 virus replication	downregulates expression of human gene	26439863,	HIV-1 infection (VSV-G pseudotyped) of CEMT4 T cells downregulates plasma membrane expression of HBB
HBB			HIV-1 virus replication	downregulates expression of human gene	26439863,	HIV-1 infection (VSV-G pseudotyped) of CEMT4 T cells downregulates plasma membrane expression of HBB
HBG1	155871	NP_057853.1	Tat	inhibits	8911578,	HIV-1 Tat inhibits the butyric acid-induced gamma-globin gene expression in human hematopoietic progenitor K562 cells
LGALS3BP	155971	NP_579894.2	Envelope surface glycoprotein gp120	downregulates	24156545,	The expression of 90K/LGALS3BP downregulates the relative amounts of mature gp120/gp41, whereas it upregulates the relative levels of uncleaved gp160 precursor inhibiting incorporation of the viral gp120/gp41 glycoproteins into progeny virions
LGALS3BP	155971	NP_579894.2	Envelope surface glycoprotein gp120	inhibited by	24156545,	The two central protein-binding domains (residues 127-409) of 90K/LGALS3BP are required for inhibition of gp160 processing and incorporation of the viral gp120/gp41 glycoproteins into progeny virions
LGALS3BP	155971	NP_057856.1	Envelope surface glycoprotein gp160, precursor	inhibited by	24156545,	The two central protein-binding domains (residues 127-409) of 90K/LGALS3BP are required for inhibition of gp160 processing and incorporation of the viral gp120/gp41 glycoproteins into progeny virions
LGALS3BP	155971	NP_057856.1	Envelope surface glycoprotein gp160, precursor	upregulates	24156545,	The expression of 90K/LGALS3BP downregulates the relative amounts of mature gp120/gp41, whereas it upregulates the relative levels of uncleaved gp160 precursor inhibiting incorporation of the viral gp120/gp41 glycoproteins into progeny virions
LGALS3BP	155971	NP_579895.1	Envelope transmembrane glycoprotein gp41	downregulates	24156545,	The expression of 90K/LGALS3BP downregulates the relative amounts of mature gp120/gp41, whereas it upregulates the relative levels of uncleaved gp160 precursor inhibiting incorporation of the viral gp120/gp41 glycoproteins into progeny virions

LGALS3BP	155971	NP_579895.1	Envelope transmembrane glycoprotein gp41	inhibited by	23156545,	The two central protein-binding domains (residues 127-409) of 90K/LGALS3BP are required for inhibition of gp160 processing and incorporation of the viral gp120/gp41 glycoproteins into progeny virions
LGALS3BP	155030	NP_057850.1	Pr55(Gag)	binds	27604950,	HIV-1 Gag binds to LGALS3BP (M2BP)
LGALS3BP	155030	NP_057850.1	Pr55(Gag)	inhibited by	27604950,	HIV-1 Gag trafficking to the plasma membrane is inhibited by LGALS3BP (M2BP) and the inhibition by LGALS3BP is dependent upon vimentin
LGALS3BP	155348	NP_789740.1	Pol	interacts with	22190034,	HIV-1 Pol is identified to have a physical interaction with lectin, galactoside-binding, soluble, 3 binding protein (LGALS3BP) in human HEK293 and/or Jurkat cell lines by using affinity tagging and purification mass spectrometry analyses
LGALS3BP			HIV-1 virus replication	inhibited by expression of human gene	24156545,	Knockdown of 90K/LGALS3BP by siRNA enhances replication and infectivity of HIV-1 in TZM-bl cells and primary macrophages
LGALS3BP			HIV-1 virus replication	inhibited by expression of human gene	27604950,	HIV-1 replication is inhibited by LGALS3BP (M2BP) overexpression in 293HEK cells
PTPRJ	155871	NP_057853.1	Tat	upregulates	20139419,	The expression of protein tyrosine phosphatase, receptor type, J (PTPRJ; CD148) gene is upregulated in both Jurkat-Tat101 and Jurkat-Tat72 cells
PTPRJ			HIV-1 virus replication	enhanced by expression of human gene	18854154,	Knockdown of protein tyrosine phosphatase, receptor type J (PTPRJ) by siRNA inhibits the early stages of HIV-1 replication in 293T cells infected with VSV-G pseudotyped HIV-1
POSTN	155871	NP_057853.1	Tat	downregulates	24667918,	Microarray analysis indicates HIV-1 Tat-induced downregulation of periostin, osteoblast specific factor (POSTN) in primary human brain microvascular endothelial cells
KHSRP	155971	NP_579894.2	Envelope surface glycoprotein gp120	complexes with	23125841,	Tandem affinity purification and mass spectrometry analysis identify KH-type splicing regulatory protein (KHSRP; FUBP2), HIV-1 Gag, Gag/Pol, gp120, and Nef incorporated into staufen1 RNP complexes isolated from HIV-1-expressing cells
KHSRP	155030	NP_057850.1	Pr55(Gag)	complexes with	23125841,	Tandem affinity purification and mass spectrometry analysis identify KH-type splicing regulatory protein (KHSRP; FUBP2), HIV-1 Gag, Gag/Pol, gp120, and Nef incorporated into staufen1 RNP complexes isolated from HIV-1-expressing cells
KHSRP	155348	NP_057849.4	Gag-Pol	complexes with	23125841,	Tandem affinity purification and mass spectrometry analysis identify KH-type splicing regulatory protein (KHSRP; FUBP2), HIV-1 Gag, Gag/Pol, gp120, and Nef incorporated into staufen1 RNP complexes isolated from HIV-1-expressing cells
KHSRP	156110	NP_057857.2	Nef	complexes with	23125841,	Tandem affinity purification and mass spectrometry analysis identify KH-type splicing regulatory protein (KHSRP; FUBP2), HIV-1 Gag, Gag/Pol, gp120, and Nef incorporated into staufen1 RNP complexes isolated from HIV-1-expressing cells
KHSRP	155807	NP_057852.2	Vpr	upregulates	23874603,	A stable-isotope labeling by amino acids in cell culture coupled with mass spectrometry-based proteomics identifies upregulation of KH-type splicing regulatory protein (KHSRP, FUBP2) expression by HIV-1 Vpr in Vpr transduced macrophages
CFHR5			HIV-1 virus replication	enhanced by expression of human gene	18976975,	Knockdown of complement factor H-related 5 (CFHR5) by siRNA inhibits HIV-1 replication in HeLa P4/R5 cells
FETUB	155971	NP_057856.1	Envelope surface glycoprotein gp160, precursor	binds	1284814,	HIV-1 gp160 binds to the natural glycoprotein fetuin
HPSE	155871	NP_057853.1	Tat	interacts with	15264223,	Treatment of synaptosomes with heparanase and HIV-1 Tat increases Tat-induced oxidative stress, which indicates the requirement of Tat interaction with neuronal membranes to induce oxidative damage
TLN1	155030	NP_579876.2	matrix	interacts with	22017400,	HIV-1 MA co-localizes with beta2 integrin, alphaM and alphaX integrins in the intracellular thick electron-dense membrane compartments, which contain talin, vinculin and paxillin that connect the integrin complexes to the actin cytoskeleton
TLN1	155348	NP_705926.1	retropepsin	cleaves	12119179,	A number of focal adhesion plaque proteins are specifically cleaved by HIV-1 protease, including fimbrin, focal adhesion plaque kinase (FAK), talin, and, to a lesser extent, filamin, spectrin and fibronectin
