# Supplementary material for: Dynamics of the blood plasma proteome during hyperacute HIV-1 infection
Source: Nat Commun. 2024 Dec 5;15:10593. doi: 10.1038/s41467-024-54848-0 (PMC11618498; doi:10.1038/s41467-024-54848-0)
Supplement: Supplementary file 7 — Reporting Summary [file 41467_2024_54848_MOESM7_ESM.pdf]

nature portfolio

Corresponding author(s): JOAKIM ESBJÖRNSSON (NCOMMS-24-19732A)

Last updated by author(s): Oct 23, 2024

Reporting Summary

Nature Portfolio wishes to improve the reproducibility of the work that we publish. This form provides structure for consistency and transparency in reporting. For further information on Nature Portfolio policies, see our Editorial Policies and the Editorial Policy Checklist.

Statistics

For all statistical analyses, confirm that the following items are present in the figure legend, table legend, main text, or Methods section.

n/a	Confirmed	
[ ]	[x]	The exact sample size (*n*) for each experimental group/condition, given as a discrete number and unit of measurement
[ ]	[x]	A statement on whether measurements were taken from distinct samples or whether the same sample was measured repeatedly
[ ]	[x]	The statistical test(s) used AND whether they are one- or two-sided<br>*Only common tests should be described solely by name; describe more complex techniques in the Methods section.*
[ ]	[x]	A description of all covariates tested
[ ]	[x]	A description of any assumptions or corrections, such as tests of normality and adjustment for multiple comparisons
[ ]	[x]	A full description of the statistical parameters including central tendency (e.g. means) or other basic estimates (e.g. regression coefficient) AND variation (e.g. standard deviation) or associated estimates of uncertainty (e.g. confidence intervals)
[ ]	[x]	For null hypothesis testing, the test statistic (e.g. *F*, *t*, *r*) with confidence intervals, effect sizes, degrees of freedom and *P* value noted<br>*Give P values as exact values whenever suitable.*
[x]	[ ]	For Bayesian analysis, information on the choice of priors and Markov chain Monte Carlo settings
[x]	[ ]	For hierarchical and complex designs, identification of the appropriate level for tests and full reporting of outcomes
[x]	[ ]	Estimates of effect sizes (e.g. Cohen's *d*, Pearson's *r*), indicating how they were calculated

Our web collection on statistics for biologists contains articles on many of the points above.

Software and code

Policy information about availability of computer code

Data collection

Commercial Software:
a. Xcalibur (Thermo Fisher Scientific): This was used for mass spectrometry data acquisition and interpretation. Version 4.1 was used in this study.

b. Instrumentation: Dionex Ultimate 3000 RSLCnano UPLC coupled to an Exploris 480 mass spectrometer with FAIMS (Thermo Fischer Scientific)
i. Column setting: trap column (PN 164535), anal column (ES802A)
ii. LC-MS/MS analysis (DIA): DIA-26 variable windows + FAIMS with 2 CVs (-45V & -60V), 90 min NL gradient
iii. nLC – non-linear gradient, 1 min at 5% B, in 75 min up to 25% B, in 9 min up to 32% B, in 6 min up to 45% B, in 2 min up to 95% B, 5 min at 95% B and 12 min equilibration at 5% B
iv. full MS - resolution: 120.000, normalized AGC target: 300%, maxIT: 45 ms, 380-1100 m/z, profile
v. DIA – 26 windows with variable width, resolution: 30.000, normalized AGC target: 1000%, maxIT: auto, NCE: 32, centroid
vi. Method file: C:\\Xcalibur\methods\MR\Tune_vs3\FAIMS_DIA_90minNLG_26vv_vs1.meth
vii. Injection: equal sample amount (1ug peptide on column)
viii. LC-MS/MS analysis (DDA): DDA-max speed (1.7s + 1.3s cycle time) FAIMS with 2 CVs (-45V & -60V), 90 min NL gradient
ix. nLC – non-linear gradient, 1 min at 5% B, in 75 min up to 25% B, in 9 min up to 32% B, in 6 min up to 45% B, in 2 min up to 95% B, 5 min at 95% B and 12 min equilibration at 5% B
x. full MS - resolution: 120.000, normalized AGC target: 300%, maxIT: 45 ms, 350-1400 m/z, profile
xi. MS/MS – cycle time (1.7/1.3s), isolation window: 1.6 m/z, resolution: 30.000, normalized AGC target: 100%, maxIT: 45 auto, intensity threshold: 2e4, NCE: 30, 45 s dynamic exclusion, centroid

	xii. Method file: C:\\Xcalibur\methods\MR\Tune_vs3 \FAIMS_2CVs_DDA_3scycle_90minNLG_45sDE_2e4_120k_45ms_MS2_30k_auto_NCE30.meth<br>xiii. Injection: ca. 1ug peptide on column
Data analysis	1. Commercial Software<br>a. Spectronaut (Biognosys): Used for analysing data-independent acquisition (DIA) proteomics experiments, peptide identification and statistical analysis. Version 15.1 was utilised in this study.<br><br>2. Open-source Software<br>a. MaxLFQ algorithm in the IQ R package was used to estimate relative protein abundances from precursor ion or peptide quantification. Version 1.8 was utilised in this study.<br><br>3. Custom Code<br>a. R Scripts: Bespoke scripts were developed in-house to preprocess raw mass spectrometry data, perform quality control checks, and extract relevant features for downstream analysis. These scripts were written in R (version 4.0) programming language, leveraging libraries such as Bioconductor. Custom R scripts were also used for statistical analysis, data visualization, and machine learning models. All the libraries used for quality control and their respective versions can be cloned from a Github repository; https://github.com/NBISweden/SMS-4964-19-hiv.git . For other downstream analysis, the code is available on https://github.com/NBISweden/SMS-5800-HIV.git. Currently available at https://lu.box.com/s/dcdu1v1iw6gyvy0bp6jsya59e1tm5fp6.

For manuscripts utilizing custom algorithms or software that are central to the research but not yet described in published literature, software must be made available to editors and reviewers. We strongly encourage code deposition in a community repository (e.g. GitHub). See the Nature Portfolio guidelines for submitting code & software for further information.

Data

Policy information about availability of data

All manuscripts must include a data availability statement. This statement should provide the following information, where applicable:

- Accession codes, unique identifiers, or web links for publicly available datasets
- A description of any restrictions on data availability
- For clinical datasets or third party data, please ensure that the statement adheres to our policy

Proteomics data supporting findings in this study have been deposited to the ProteomeXchange Consortium via PRoteomics IDEntifications (PRIDE) partner repository with the dataset identifier PXD042850 (https://www.ebi.ac.uk/pride/archive/projects/PXD042850) .

Research involving human participants, their data, or biological material

Policy information about studies with human participants or human data. See also policy information about sex, gender (identity/presentation), and sexual orientation and race, ethnicity and racism.

Reporting on sex and gender	These findings apply for both males and females. Sex information was collected in our study using self-report. Additionally, we have taken measures to ensure that data analysis considers both biological sex and gender-related variables, where applicable.<br><br>In the source data of our proteomics study examining plasma proteomes in acute HIV-1 infection, sex and gender data have been disaggregated where this information has been collected. Participants were asked to self-report their sex assigned at birth and gender identity. Consent was obtained from all participants for the sharing of individual-level data. All data presented here have been anonymized to ensure participant confidentiality and privacy. Overall numbers for sex and gender disaggregation in the reporting summary are as follows:<br>Total number of participants: 54<br>Number of participants identifying as male: 34<br>Number of participants identifying as female: 20<br><br>Justification for lack of sex and gender-based analysis:<br>It is important to note that due to the specific focus of our study on plasma proteomics and the nature of the data available, there may be limitations in conducting comprehensive sex-based analyses. The demographics of our study population may also have impacted our ability to conduct this kind of analyses. In our case, one of the cohorts from South Africa was predominantly females, while the East African cohort was predominantly males. These differences were also considered as co-variates in the models that were used in data analysis. Therefore, our sample size and study design may not have provided sufficient statistical power or granularity to perform robust sex-specific analyses.
Reporting on race, ethnicity, or other socially relevant groupings	We did not utilize any socially constructed or socially relevant categorization variables. We recognize the importance of considering such variables in biomedical research, as they can provide valuable insights into disparities and inequalities in health outcomes. However, for the specific focus of our study on plasma proteomics in acute HIV-1 infection, our primary aim was to investigate molecular mechanisms and identify potential biomarkers associated with disease progression.<br><br>We did not incorporate variables such as race, ethnicity, socioeconomic status, or other socially constructed categories into our analysis. While we acknowledge the significance of these variables in understanding broader health disparities, they were not relevant to the specific research questions addressed in our manuscript.
Population characteristics	The human research participants exhibited diverse covariate-relevant population characteristics, which were essential for understanding disease progression. These characteristics included:

	Age: Participants ranged in age from 18 to 52 years, with a median age of 25 years. Age as covariate was considered in the risk analysis for disease progression.<br>Past diagnosis and treatment categories: Participants' medical histories were assessed to identify past diagnoses, previous treatments, and disease management strategies. This included information on prior HIV diagnoses, antiretroviral therapy regimens. Inormation on treatment start dates was used to exclude several data points in risk analyses. Participants underwent thorough clinical evaluations to determine current HIV-1 status, disease stage, and treatment status. This included assessments of viral load, CD4+ T-cell count, HIV-1 subtype, and current antiretroviral therapy regimen.
Recruitment	Clinical settings: Participants were recruited from HIV clinics, infectious disease clinics, and other healthcare facilities specializing in HIV care. Healthcare providers identified eligible individuals based on specific inclusion criteria and referred them to the research team for potential participation.<br><br>Community outreach: We conducted community outreach efforts to raise awareness about the study and engage potential participants from the broader community affected by HIV-1. This included distributing informational materials at community events, partnering with local organizations serving HIV-1 affected populations<br><br>Participant registries: We collaborated with existing participant registries and research networks specializing in HIV/AIDS to identify eligible individuals who may be interested in participating in our study. These registries maintained comprehensive databases of individuals living with HIV-1 and provided a valuable resource for recruitment purposes.
Ethics oversight	Prior to enrollment, all volunteers underwent an informed consent procedure and written informed consent was documented. Ethical approvals in each country were obtained from the Kenya Medical Research Institute Ethical Review Committee, the Kenyatta National Hospital Ethical Review Committee of the University of Nairobi, the Rwanda National Ethics Committee, the Uganda Virus Research Institute Science and Ethics Committee, the Uganda National Council of Science and Technology, the University of Cape Town Health Science Research and Ethics Committee, the University of Zambia Research Ethics Committee, the Bio-Medical Research Ethics Committee at the University of KwaZulu Natal, and the Emory University Institutional Review Board. The study in south Africa was approved by biomedical research ethics committee of the University of KwaZulu-Natal and the institutional review board of Massachusetts General Hospital.

Note that full information on the approval of the study protocol must also be provided in the manuscript.

Field-specific reporting

Please select the one below that is the best fit for your research. If you are not sure, read the appropriate sections before making your selection.

☒ Life sciences ☐ Behavioural & social sciences ☐ Ecological, evolutionary & environmental sciences

For a reference copy of the document with all sections, see nature.com/documents/nr-reporting-summary-flat.pdf

Life sciences study design

All studies must disclose on these points even when the disclosure is negative.

Sample size	The sample size was determined based on specific inclusion criteria and the availability of participant samples at key time points. The following criteria were used to select participants for inclusion in the study:<br><br>Availability of plasma samples collected at two weeks after infection: All individuals who had plasma samples available for collection at two weeks after HIV-1 infection were eligible for inclusion in the study. This time point was chosen to capture early changes in plasma proteomes following acute HIV-1 infection and to establish baseline measurements for subsequent analyses.<br><br>Availability of pre-infection samples: In addition to samples collected at two weeks after infection, participants were required to have pre-infection plasma samples available for analysis. Pre-infection samples served as baseline measurements to compare against proteomic profiles observed at two weeks after infection, allowing for the identification of early biomarkers or changes in protein expression associated with HIV-1 acquisition.<br><br>Availability of one-month post-infection samples: Participants were also required to have plasma samples collected at one month post-infection. This time point provided additional insight into the dynamics of plasma proteomes during the early stages of HIV-1 infection and allowed for longitudinal analyses of protein expression patterns over time.<br><br>The decision to include all individuals meeting these criteria, rather than predetermining a specific sample size through statistical methods, was based on several factors:<br>-Limited availability of samples: Due to the challenges associated with sample collection and participant recruitment in the context of acute HIV-1 infection, our study leveraged available samples from individuals who met the specified inclusion criteria. As such, the sample size was determined by the number of eligible participants with available samples meeting these criteria.<br>-Importance of longitudinal analyses: Given the longitudinal nature of the study and the need to assess changes in plasma proteomes over time, including samples collected at multiple time points (i.e., pre-infection, two weeks after infection, one month post-infection) was deemed essential. This approach allowed for within-subject comparisons and increased statistical power to detect meaningful differences in protein expression patterns over time.<br><br>Feasibility and practical considerations: Given the logistical constraints associated with sample collection and processing in the context of acute HIV-1 infection, it was not feasible to predetermine a specific sample size a priori. Instead, our study utilized all available samples meeting the specified inclusion criteria to maximize the use of existing resources and ensure the robustness of our analyses.

While the sample size in our study was not determined through formal statistical methods, the inclusion of samples meeting stringent criteria for time point availability (i.e., pre-infection, two weeks after infection, one month post-infection) ensured a comprehensive and representative dataset for investigating plasma proteomes in acute HIV-1 infection. By leveraging available samples meeting these criteria, we aimed to maximize the scientific rigor and validity of our study findings.

Data exclusions

The decision to not exclude any data was based on several factors:
Inclusion criteria: Data were collected and included in the analyses based on predefined inclusion criteria, including the availability of plasma samples collected at specific time points (i.e., pre-infection, two weeks after infection, one month post-infection). These criteria were established a priori to ensure consistency and rigor in participant selection and data collection.

Data quality control: Prior to analysis, data underwent rigorous quality control procedures to assess data completeness, accuracy, and reliability. Any discrepancies or inconsistencies identified during quality control checks were addressed through data validation and verification processes, rather than data exclusion.

Replication

We implemented several measures to verify the reproducibility of our experimental findings. These measures included:
1. Independent replication of experiments: Where feasible, key experiments and analyses were independently replicated by multiple researchers within our team to confirm the consistency and reliability of our findings. This involved repeating experiments using identical protocols, reagents, and conditions to assess the reproducibility of results across different experimental runs.

2. Internal validation procedures: We conducted internal validation procedures, such as cross-validation and bootstrapping analyses, to assess the robustness of our findings and identify any potential sources of variability or bias. These validation procedures helped ensure that our results were not overly influenced by random variation or confounding factors.

3. Open data and code sharing: We adhered to principles of open science by making our data, analytical code, and protocols freely available to the research community. The analysis was also performed with reroduciblity in mind. This transparency allowed other researchers to independently verify our findings and reproduce our analyses using the same data and methods.

4. Methodological transparency: Detailed documentation of experimental protocols, analytical workflows, and data analysis procedures was maintained to ensure methodological transparency and facilitate reproducibility. This included providing step-by-step descriptions of sample processing, data acquisition, and data analysis, as well as sharing code and software tools used in the study.

Randomization

In our study on plasma proteomes in acute HIV-1 infection, the allocation of participants into experimental groups was based on specific criteria related to the timing of sample collection relative to HIV-1 infection and the availability of pre-infection and post-infection samples. Given the nature of the study design and the focus on longitudinal analysis of plasma proteomes, random allocation of participants into experimental groups was not applicable.

Allocation criteria was as follows:

1.Timing of sample collection: Participants were allocated into experimental groups based on the timing of sample collection relative to HIV-1 infection. Specifically, individuals with available plasma samples collected at two weeks after infection, pre-infection, and one month post-infection were included in the study. This allocation ensured that samples were representative of different stages of acute HIV-1 infection, allowing for longitudinal analysis of protein expression patterns over time.

2. Availability of pre-infection and post-infection samples: In addition to the timing of sample collection, allocation into experimental groups was contingent upon the availability of pre-infection and post-infection samples from each participant. Participants with complete sets of plasma samples spanning pre-infection, two weeks after infection, and one month post-infection were included in the study, enabling comprehensive longitudinal analysis of plasma proteomes.

Control of covariates:
While random allocation of participants into experimental groups was not feasible due to the longitudinal nature of the study and the specific criteria for sample collection, we implemented rigorous control measures to account for potential confounding variables and covariates. This included careful matching of participants based on key demographic and clinical characteristics (e.g., age, sex, CD4 cell count, viral load) to minimize bias and ensure comparability between experimental groups. Additionally, statistical techniques such as regression analysis were employed to control for potential confounders and covariates in the analysis of experimental outcomes.

Blinding

Blinding of investigators to group allocation during data collection and/or analysis was not applicable. This is because the allocation of participants into experimental groups was based on specific criteria related to the timing of sample collection relative to HIV-1 infection and the availability of pre-infection and post-infection samples, rather than randomized allocation.

Explanations:
1. Non-randomized allocation: Participants were allocated into experimental groups based on predetermined criteria related to the timing of sample collection and the availability of pre-infection and post-infection samples. This non-randomized allocation method precluded the need for blinding of investigators, as group allocation was determined by objective criteria rather than random assignment.

2. Objective measurement: The primary outcome of interest in our study was changes in plasma proteomes over time in response to acute HIV-1 infection. This outcome was assessed using objective biochemical assays and mass spectrometry-based proteomic analysis, which do not require subjective interpretation or judgment by investigators. As such, blinding of investigators to group allocation was not necessary for data collection or analysis.

3. Study design: Given the longitudinal nature of the study and the focus on characterizing changes in plasma proteomes over time, blinding of investigators to group allocation was not feasible or relevant. The study design and objectives necessitated a comprehensive assessment of proteomic profiles at different stages of acute HIV-1 infection, which required full awareness of sample allocation and timing.

It is worth to mention that the samples were randomised before quantification on the mass spectrometer and the scientist running the samples couldnot decode the time of collection or patient ID based on the labID

Reporting for specific materials, systems and methods

We require information from authors about some types of materials, experimental systems and methods used in many studies. Here, indicate whether each material, system or method listed is relevant to your study. If you are not sure if a list item applies to your research, read the appropriate section before selecting a response.

Materials & experimental systems

n/a	Involved in the study
☒	☐ Antibodies
☒	☐ Eukaryotic cell lines
☒	☐ Palaeontology and archaeology
☒	☐ Animals and other organisms
☒	☐ Clinical data
☒	☐ Dual use research of concern
☒	☐ Plants

Methods

n/a	Involved in the study
☒	☐ ChIP-seq
☒	☐ Flow cytometry
☒	☐ MRI-based neuroimaging

Plants

Seed stocks

Report on the source of all seed stocks or other plant material used. If applicable, state the seed stock centre and catalogue number. If plant specimens were collected from the field, describe the collection location, date and sampling procedures.

Novel plant genotypes

Describe the methods by which all novel plant genotypes were produced. This includes those generated by transgenic approaches, gene editing, chemical/radiation-based mutagenesis and hybridization. For transgenic lines, describe the transformation method, the number of independent lines analyzed and the generation upon which experiments were performed. For gene-edited lines, describe the editor used, the endogenous sequence targeted for editing, the targeting guide RNA sequence (if applicable) and how the editor was applied.

Authentication

Describe any authentication procedures for each seed stock used or novel genotype generated. Describe any experiments used to assess the effect of a mutation and, where applicable, how potential secondary effects (e.g. second site T-DNA insertions, mosiacism, off-target gene editing) were examined.
